# The association of regional cerebral blood flow and glucose metabolism in normative ageing and insulin resistance

**DOI:** 10.1038/s41598-024-65396-4

**Published:** 2024-06-25

**Authors:** Hamish A. Deery, Emma Liang, Robert Di Paolo, Katharina Voigt, Gerard Murray, M. Navyaan Siddiqui, Gary F. Egan, Chris Moran, Sharna D. Jamadar

**Affiliations:** 1 School of Psychological Sciences, Monash University, Wellington Rd, Melbourne, 3800 Australia; 2 Monash Biomedical Imaging, Monash University, 770 Blackburn Rd, Melbourne, 3800 Australia; 3 School of Public Health and Preventive Medicine, Monash University, 553 St Kilda Rd, Melbourne, VIC 3004 Australia

**Keywords:** Cerebral blood flow, Cerebral glucose metabolism, Healthy ageing, Insulin sensitivity, Insulin resistance, Neuronal physiology, Molecular neuroscience

## Abstract

Rising rates of insulin resistance and an ageing population are set to exact an increasing toll on individuals and society. Here we examine the contribution of age and insulin resistance to the association of cerebral blood flow and glucose metabolism; both critical process in the supply of energy for the brain. Thirty-four younger (20–42 years) and 41 older (66–86 years) healthy adults underwent a simultaneous resting state MR/PET scan, including arterial spin labelling. Rates of cerebral blood flow and glucose metabolism were derived using a functional atlas of 100 brain regions. Older adults had lower cerebral blood flow than younger adults in 95 regions, reducing to 36 regions after controlling for cortical atrophy and blood pressure. Lower cerebral blood flow was also associated with worse working memory and slower reaction time in tasks requiring cognitive flexibility and response inhibition. Younger and older insulin sensitive adults showed small, negative correlations between relatively high rates of regional cerebral blood flow and glucose metabolism. This pattern was inverted in insulin resistant older adults, who showed hypoperfusion and hypometabolism across the cortex, and a positive correlation. In insulin resistant younger adults, the association showed inversion to positive correlations, although not to the extent seen in older adults. Our findings suggest that the normal course of ageing and insulin resistance alter the rates of and associations between cerebral blood flow and glucose metabolism. They underscore the criticality of insulin sensitivity to brain health across the adult lifespan.

## Introduction

One of society’s most pressing challenges is dealing with the health, social and economic costs of non-communicable or “lifestyle diseases”^1,2^. Historically, conditions such as obesity, insulin resistance and type 2 diabetes, have been associated with older age and economic development, but they are increasingly prevalent in younger adults and low- and middle-income countries^3^. People with these conditions are at increased risk for a range of other health issues, including cognitive decline^4^, making their prevention and management priorities for governments and health authorities around the world^2^.

Due to the limited ability of the brain to store energy, it is susceptible to factors that influence real-time energy supply. One such factor is insulin. Although the brain is not insulin-dependent in terms of glucose uptake via GLUT4, insulin crosses the blood brain barrier by a saturable mechanism and via the cerebrospinal fluid (^5^; also see^6^ for review). Insulin receptors are present in most brain regions and their signalling contributes to many physiological and behavioural processes, such as feeding, learning and memory (see^7^ for review).

Some people are more resistant to the action of insulin than others. Lifestyle conditions and associated behaviours, such as low physical activity and poor diet, are associated with increased resistance to insulin’s action^8^. Clinical and sub-clinical levels of insulin resistance are associated with changes to the brain^9^. For example, insulin resistance is a signature of Alzheimer’s disease and type 2 diabetes and is associated with deleterious changes to the brain’s structure, vasculature and metabolism (for reviews see^4,10^). Increased grey matter atrophy and cognitive decline have been found with higher levels of insulin resistance, even in the absence of comorbidities^11,12^. Elevated glycaemia in otherwise healthy individuals in their 20–40 s can cause grey matter atrophy, reduced white matter integrity and impaired cognition^13–15^.

In the human brain, a tight coupling is required between neuronal activity and cerebral blood flow (CBF) for the delivery of oxygenated blood, glucose and other nutrients to neurons^16^. While differences in the absolute rates of CBF and glucose metabolism have been well studied in ageing and disease, less is known about their coupling. For example, older adults have lower absolute CBF and cerebral metabolic rates of glucose (CMR_GLC_) than younger adults^17–19^, particularly in the frontal and temporal regions^18,20^. Lower rates of CBF have been associated with increased glycemia, insulin resistance and adiposity in otherwise healthy middle aged and older adults^21–26^. Longitudinal declines in CBF have been associated with reduced processing speed in older adults^27^. The links between insulin resistance and reduced CMR_GLC_ are also well established (see^9^, for review). In recent work, we demonstrated that older adults had lower CMR_GLC_ than younger adults, but insulin resistance significantly reduced CMR_GLC_ in younger but not older adults^28^.

Insulin mediates peripheral vascular functions (e.g., vasodilation) via its receptors and signalling pathways, and is associated with vascular complications in insulin resistance (see^29^ for review of mechanism). However, insulin’s role in the brain vasculature is less well understood. The administration of intranasal insulin has a vasoactive effect in people with type 2 diabetics^30^. Insulin appears to influence CMR_GLC_, and CBF coupling (see^31^ for a review), possibly via astrocytic receptors^32^. In terms of age differences in the CBF and CMR_GCL_ association, Bentourkia et al. reported that older people had lower CBF and CMR_GLC_ than younger people, but the correlation between CBF and CMR_GLC_ was similar in both groups^33^. In contrast, Henriksen et al. found an association between CBF and CMR_GLC_ at the regional but not the whole brain level in older adults^34^.

In the current study we examined the rates of and associations between cerebral blood flow and glucose metabolism in healthy older and younger adults and explored the contribution of insulin resistance and age to these associations. We also examine the association of CBF and cognition. We hypothesised that older age and higher insulin resistance are associated with lower CBF, and the effects of older age and insulin resistance are mediated by cortical thickness and blood pressure. We hypothesised that lower CBF is associated with worse cognitive performance. We also hypothesised that the relationship between CBF and CMR_GLC_ is stronger in younger than older adults. We further hypothesised that CBF and CMR_GLC_ associations are strongest in those with the least insulin resistance and weakest in those with the most insulin resistance. Finally, we hypothesised that there are spatial differences in the associations of CBF and CMR_GLC_ for older versus younger adults and for levels of insulin resistance.

## Results

### Demographic factors

There was no statistically significant difference in sex, years of education or body mass index between the older and younger adult groups (Table 1). However, older adults had lower whole brain cortical thickness and higher systolic blood pressure (*p* < 0.001) and resting heart rate (*p* < 0.05) compared to younger adults. Nine younger and 26 older participants would meet the definition for hypertension from the measurements taken, i.e., a systolic blood pressure of 140 mmHg or higher, a diastolic blood pressure 90 mmHg or higher, or both^35^. Because of the known impact of hypertension on the brain, we included diastolic and systolic blood pressure as covariates in our analyses.

**Table 1:** Demographics comparison of younger and older groups and four sub-groups based on age category and HOMA-IR median split.

	Age category: younger vs older			4 groups: age category and HOMA-IR median split				
	Younger (N = 34)	Older (N = 41)	*p*^1^	Younger insulin sensitive (n = 14)	Younger insulin resistant (n = 20)	Older insulin sensitive (n = 25)	Older insulin resistant (n = 16)	*p*^1^
Age (years)	28.2 (6.3)	75.7 (5.8)	< 0.001	29.6 (6.1)	27.2 (6.3)	75.3 (6)	76.3 (5.5)	< 0.001
Sex—Number of females (%)	19 (56%)	18 (44%)	0.302	9 (64%)	10 (50%)	14 (56%)	4 (25%)	0.139
Years of education	18.2 (2.9)	16.9 (3.7)	0.125	19.2 (2.8)	17.4 (2.7)	16.1 (2.9)	17.8 (4.5)	0.088
Insulin (mU/L)	4.8 (3.2)	4.1 (2.8)	0.280	2.2 (1.0)	6.6 (2.9)	2.3 (0.7)	6.7 (2.6)	< 0.001
Blood glucose (mmol/L)	4.7 (0.4)	5.2 (0.6)	< 0.001	4.6 (0.4)	4.8 (0.3)	4.8 (0.4)	5.6 (0.4)	< 0.001
HOMA-IR	1.0 (0.7)	1.0 (0.8)	0.821	0.4 (0.2)	1.4 (0.6)	0.5 (0.1)	1.7 (0.7)	< 0.001
HOMA-IR2	0.61 (0.41)	0.54 (0.38)	0.431	0.2 (0.1)	0.8 (0.3)	0.3 (0.1)	0.9 (0.3)	< 0.001
Systolic blood pressure (mmHg)	120.1 (17.8)	148.3 (24.6)	< 0.001	113.3 (16.3)	124.8 (17.7)	151.0 (28.0)	144.0 (18.0)	< 0.001
Diastolic blood pressure (mmHg)	79.6 (13.7)	83.3 (12.1)	0.218	73.9 (10.2)	83.6 (14.6)	86.8 (12.5)	77.8 (9.2)	0.010
Resting heart rate (BPM)	81.8 (17.2)	73.8 (13)	0.026	75.2 (16.8)	86.3 (16.2)	75.7 (13.0)	70.8 (12.6)	0.015
Body mass index (BMI)	24.2 (4.8)	25.7 (3.5)	0.127	22.7 (3.1)	25.2 (5.5)	24.7 (3.6)	27.2 (2.7)	0.029
Cortical thickness (mm)	2.52 (0.08)	2.36 (0.11)	< 0.001	2.5 (0.07)	2.5 (0.07)	2.3 (0.09)	2.3 (0.13)	< 0.001

Continuous variables are mean (standard deviation); categorical variables are %.

^1^*p* values are based on ANOVA for continuous and Chi-square for categorical variables.

Significant differences were found between four groups based on age category and insulin resistance levels (HOMA-IR median split) on all demographics except sex and years of education (see Table 1). For the insulin sensitive younger and older groups, the mean HOMA-IR was 0.4 and 0.5, respectively; whereas for insulin resistant younger and older groups it was 1.4 and 1.7, respectively. A clinical diagnosis of insulin resistance is not usually made from HOMA-IR; however, thresholds have been proposed in the literature ranging from 1.8 to 2.5^36^. Four and five participants in the younger and older insulin resistant groups would meet a threshold of 1.8 HOMA-IR, respectively; and three and four participants a HOMA-IR threshold of 2.5. A higher percentage of older adults than younger adults in both the high and low HOMA-IR groups would also meet the threshold for hypertension.

Because of the known impact of the other demographic factors on the brain, we ran additional GLMs with them as covariates to test the association of CBF and CMR_GLC_ (see Supplement, section 4).

### The effect of age, cortical thickness and blood pressure on regional CBF

**Figure 1:**
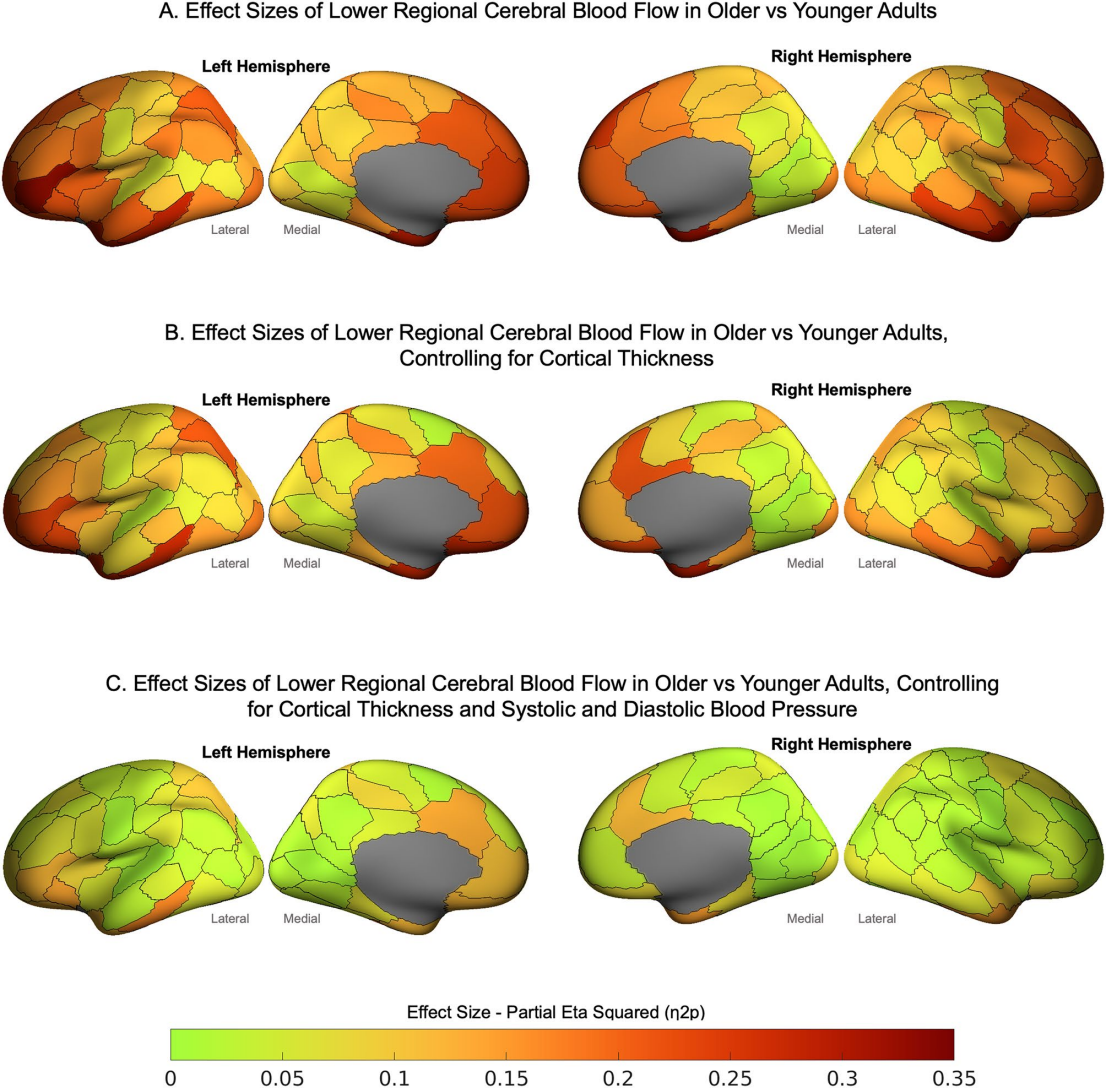
Effect sizes from general linear models showing lower regional cerebral blood flow in older vs younger adults. Cerebral blood flow is significantly lower in older than younger adults in (**A**) 95 regions, (**B**) 85 regions when cortical thickness was added as a covariate, and (C) 36 regions when cortical thickness and systolic and diastolic blood pressure were included as covariates. See Table S2 for details of GLMs.

The mean regional CBF for older and younger adults and the results of the GLMs comparing younger and older adults are reported in the Supplement (Figure S1, Tables S1 and S2). Older age was associated with lower CBF in 95 regions (p-FDR < 0.05; Fig. 1A). Older adults had lower CBF than younger adults in 85 regions (p-FDR < 0.05) when cortical thickness was added to the models (Fig. 1B). When cortical thickness and systolic and diastolic blood pressure were included, older adults had lower CBF in 36 regions (Fig. 1C). The largest age differences in CBF were in the bilateral temporal poles (0.282–0.317), temporal cortices (0.246), ventral, orbital, lateral, medial and dorsal prefrontal cortices (0.203–0.327), superior parietal lobule (0.210), and insula (0.201). Adding cortical thickness had a minimal impact on both the magnitude of the effect sizes and the regions showing the largest age differences in CBF (Table S3). However, the effect sizes reduced by approximately 40–60% in those same regions when systolic and diastolic blood pressure were also added to the models.

### The effect of age and insulin resistance on regional CBF

**Figure 2:**
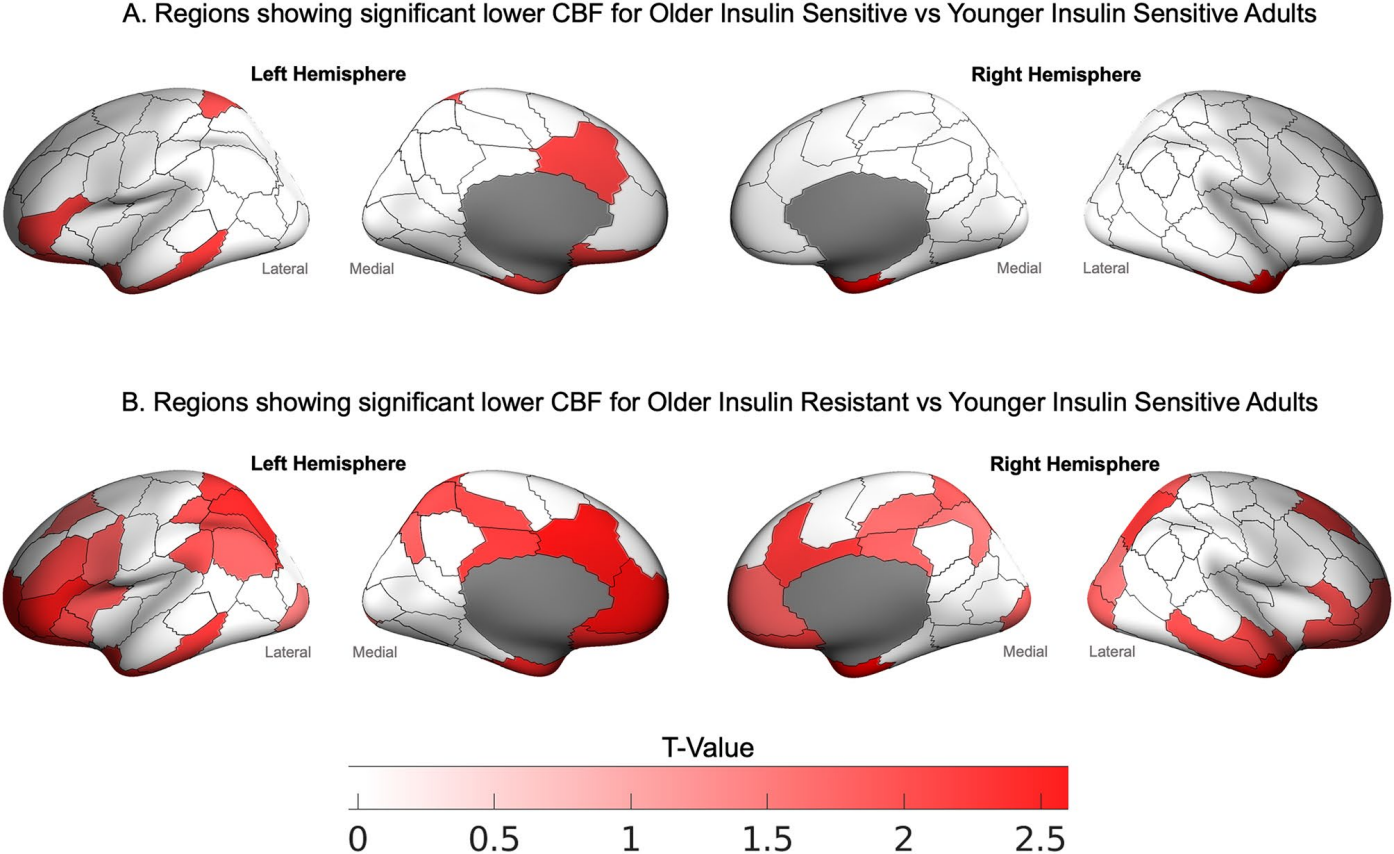
Regions showing significantly lower CBF for (**A**) Older Insulin Sensitive and (**B**) Older Insulin Resistant vs Younger Insulin Sensitive adults. T-value are plotted from the post-hoc contrasts from the GLMs among the four sub-groups. Regions showing statistically significant differences are colored red; non-significant differences are white. See Table S4 for the details of the GLMs. The mean and standard deviation of regional CBF for each group are presented in Figure S2 and Table S3.

The mean regional CBF for the four groups based on age category (younger and older) and a HOMA-IR median split (low and high HOMA-IR) and the GLMs comparing the four groups are reported in the Supplement (Figure S2 and Table S4). A significant difference was found in CBF in 40 regions between the four groups (Table S5). Post-hoc contrast showed that older insulin resistant adults had lower CBF than younger insulin sensitive adults (Fig. 2). This pattern was found in regions within all networks, except the somatomotor network. The effect size was the largest in the posterior cingulate (0.265–0.185), precuneus (0.156), temporal cortex (0.114), and lateral prefrontal cortex (0.119–0.186) in the control network; the post central (0.112–0.114) and superior parietal lobule (0.171) in the dorsal attention network; the medial and dorsal prefrontal cortices (0.110–0.209) and ventral prefrontal cortices (0.132–0.186) in the default network; the insula (0.115), parietal medial (0.152) and medial posterior prefrontal cortices (0.174–0.207) in the salience ventral attention; and the orbital frontal (0.134–0.167) and temporal poles (0.189–0.212) in the limbic network.

When the four groups were derived from age group and a HOMA-IR2 median split (younger low/high HOMA-IR2, older low/high HOMA-IR2), we found minimal to no differences in results to those based on HOMA-IR (see Supplement Table S5). This was expected as HOMA-IR2 models increases in the insulin secretion curve for plasma glucose concentrations above 10 mmol/L^37^; a threshold that than none of the participants reached. Hence, the remainder of the results are based on analyses of HOMA-IR.

### The relationship between CBF and cognition, age and HOMA-IR

**Figure 3:**
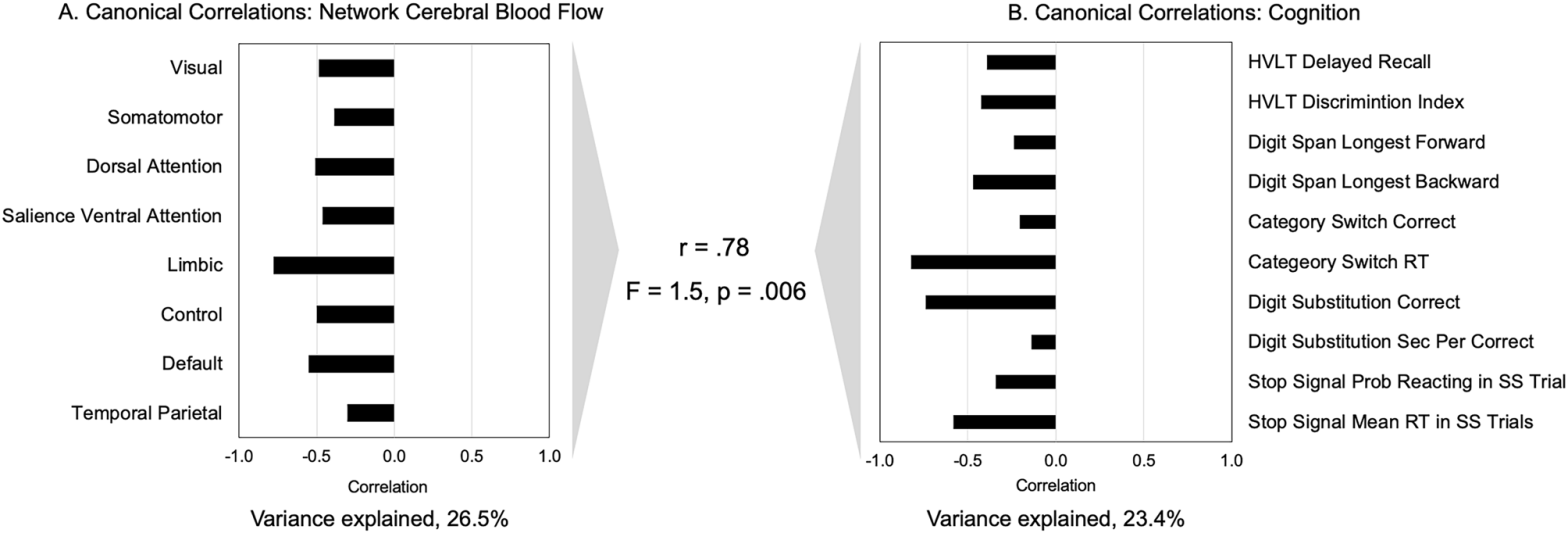
Canonical correlations between network CBF and cognitive measures. r-value is the canonical correlation between the linear combinations of the network CBF values and cognition variables that maximally covary across subjects. F-statistic is Wilk’s test of the null hypothesis that the canonical correlation and all smaller ones are equal to zero and was significant for one canonical variate. The correlations on each variable set represent the strength of the association between the variable and the canonical variate. Variance explained is the percentage of variance explained by the variables in their variate.

The canonical correlation analysis of network CBF and cognition identified one significant canonical mode (Fig. 3). The linear combinations of the network CBF and the cognition measures were significantly correlated with each other, with a correlation coefficient of 0.78 (*p* = 0.006). Lower CBF across all of the networks correlated with worse performance on the cognitive tasks. Limbic, default, control, attention and visual network CBF in particular were associated with lower reaction time in the category switch and stop signal tasks, as well as worse performance in working memory (digit span backwards) and verbal learning and memory (HVLT) tasks.

The canonical correlation was 0.63 between cognition and network CBF after controlling for age group, whole brain cortical thickness and blood pressure, but this association was not significant (F = 1,2, *p* = 0.195).

### The effect of age and HOMA-IR on the coupling of CBF and CMR_GLC_

**Figure 4:**
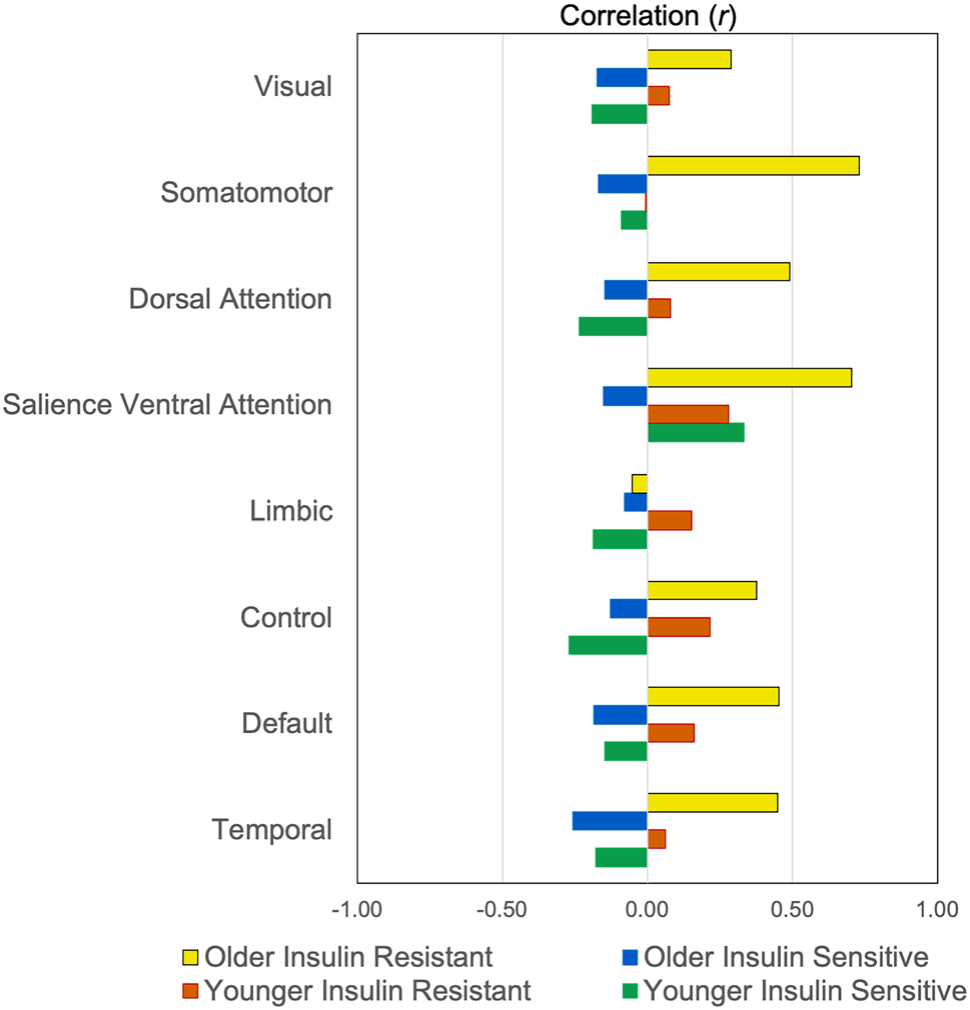
Partial correlation of cerebral blood flow (CBF) and glucose metabolism (CMR_GLC_) controlling for cortical thickness and systolic and diastolic blood pressure in the Schaefer networks for four groups: younger insulin sensitive; younger insulin resistant; older insulin sensitive; and older insulin resistant.

**Figure 5:**
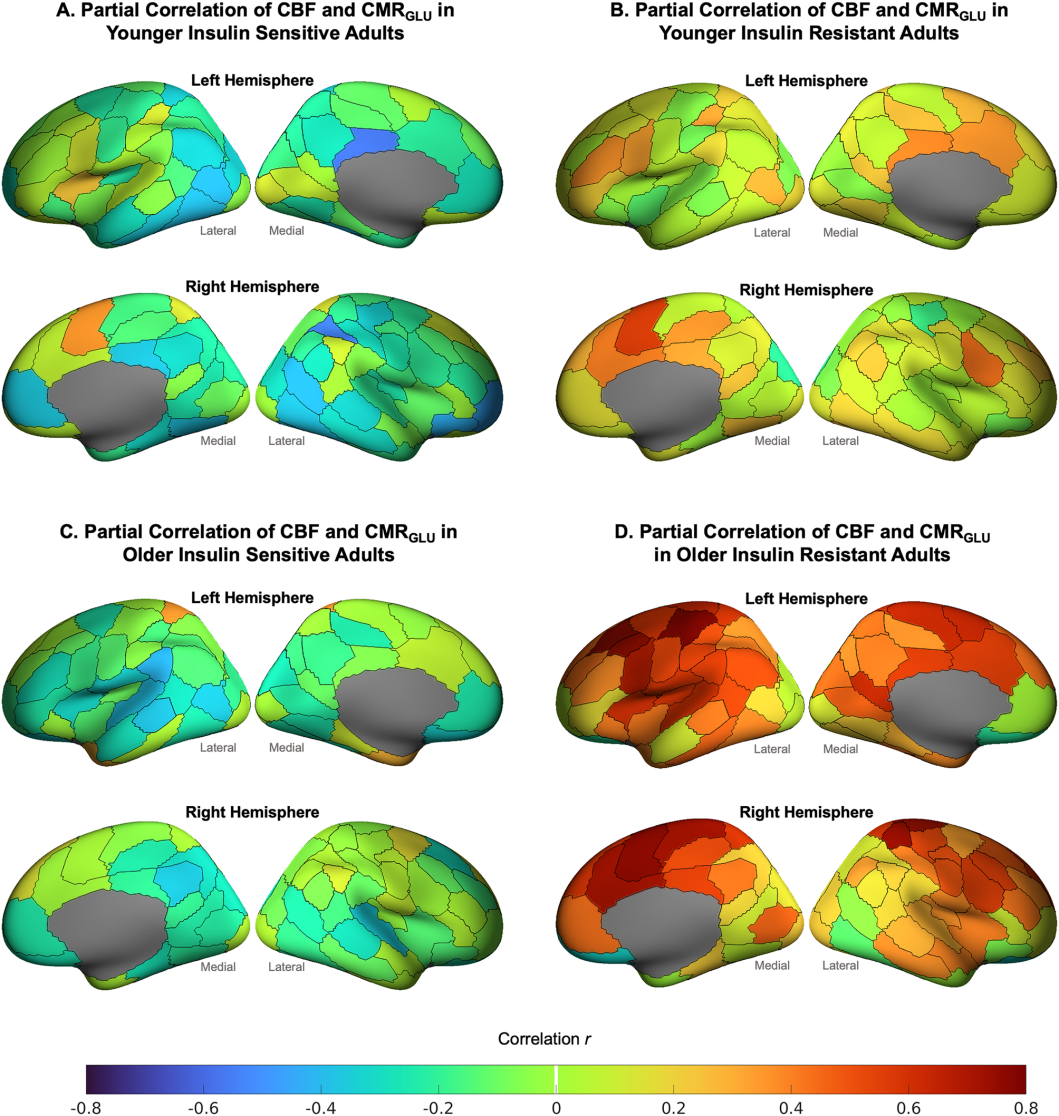
Partial correlation of regional cerebral blood flow (CBF) and glucose metabolism (CMR_GLC_) controlling for cortical thickness and systolic and diastolic blood pressure for four groups: Younger insulin sensitive (**A**), younger insulin resistant (**B**), older insulin sensitive (C), and older insulin resistant (D). See the Supplement for the mean regional CBF (Table S3 and Figure S2) and CMR_GLC_ (Table S6) for the four groups, and their correlations (Figure S3).

The mean CMR_GLU_ for older and younger adults is reported in the Table S6. When differences in the correlations between CBF and CMR_GLU_ were examined for the four groups based on age category and insulin resistance levels, we found widespread between-group differences at both the network (Fig. 4) and regional level (Fig. 5 and Figure S3). Older insulin resistant adults had strong positive correlations between CBF and CMR_GLC_ in all networks. The highest correlations were in the somatomotor and salience ventral attention networks, followed by the dorsal attention, default and control network. For younger insulin resistant adults, the correlations between CBF and CMR_GLC_ were mostly small-to-moderate positive in all networks and much lower than for the older insulin resistant adults. In contrast, both younger and older insulin sensitive adults showed small-to-moderate negative correlations between CBF and CMR_GLC_ across the networks.

The pattern of CBF and CMR_GLC_ correlations described above at the network level was reflected across many of the regions (Fig. 5 and Figure S3). Younger and older insulin sensitive adults showed primarily negative correlations, with 79 and 73 total negative correlations, and 50 and 38 correlations below − 0.2, respectively. The pattern was inverted in older insulin resistant adults, with positive correlations between CBF and CMR_GLC_ in 94 regions (79 region had an r > 0.2). Older insulin resistant adults also showed the lowest absolute rates of CBF and CMR_GLC_ among all groups (Tables S4 and S6). Younger insulin resistant adults showed smaller positive correlations in 82 regions (25 with r > 0.2).

The Pearson and Spearman correlations assessing the strength and rank of the CBF and CMR_GLC_ coupling across the 100 regions between the younger insulin sensitive and older insulin resistant groups were both significant (r = 0.31 and r = 0.27, *p* < 0.01). The DICE coefficient was 0.42 when comparing the top 50% of regional correlations and 0.24 for the top 25% of regional correlations between younger insulin sensitive and older insulin resistant groups.

The Pearson correlations approached significance for the young insulin sensitive and resistant groups (r = 0.12, *p* < 0.060). The DICE coefficient was 0.48 and 0.54 comparing the top 50% of regional correlations between the younger insulin sensitive and younger insulin resistant and older insulin resistant groups, respectively. For the top 25% of regions, the DICE coefficients were 0.16 and 0.28 for comparison of the younger insulin sensitive group with the younger insulin resistant and older insulin sensitive groups, respectively.

### The effect of age and HOMA-IR on the association of CBF and fMRI graph metrics

**Figure 6:**
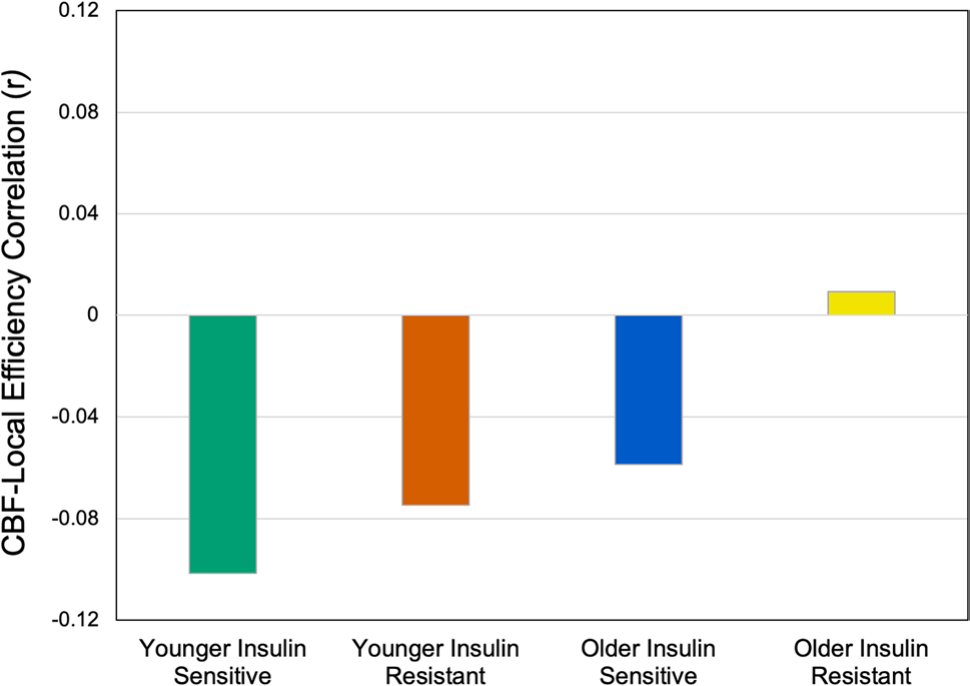
Significant CBF and local efficiency correlation for four groups based on age group and HOMA-IR median slit. Post hoc contrasts are significant for younger insulin sensitive vs older insulin sensitive and older insulin resistant groups (see Table 2). One sample T-tests also show that the correlation for younger insulin sensitive, younger insulin resistant and older insulin sensitive adults are significantly different to zero (all *p* < 0.05).

The GLM was significant for the correlation of CBF and local efficiency (Table 2). Significant differences were found between the four groups based on age category and insulin resistance levels (0.182), and diastolic blood pressure (0.064) levels. One sample T-tests showed that the correlation between CBF and local efficiency was significantly different to zero for all groups (*p* < 0.001), except the older insulin resistant adults. Post-hoc contrasts for the group effect from the GLM showed that younger insulin sensitive adults had significantly higher negative correlations between CBF and local efficiency than both older adult groups (Fig. 6), that is, higher cerebral blood flow was more strongly associated with lower local network efficiency among insulin sensitive younger adults than insulin sensitive older adults. For older insulin resistant adults, the correlation was both significantly different to younger insulin sensitive adults and weakly positive.

**Table 2:** General linear models of the correlation between CBF and fMRI graph metrics (individual correlations across the 100 regions).

	Overall model			4 Groups: age category and HOMA median split						Systolic BP			Diastolic BP			Cortical thickness		
	F	*p*	η^2^_p_	F	*p*	η^2^_p_	YIS: vs YIR	YIS: vs OIS	YIS: vs OIR	F	*p*	η^2^_p_	F	*p*	η^2^_p_	F	*p*	η^2^_p_
Correlation CBF and Global Efficiency	1.9	0.091	0.150	0.1	0.982	0.003				0.4	0.537	0.006	0.3	0.613	0.004	6.5	0.01	0.09
Correlation CBF and Local Efficiency	4.3	0.001	0.285	4.8	0.004	0.182	0.161	0.011	0.000	0.2	0.645	0.003	4.5	0.039	0.064	3.9	0.05	0.06
Correlation CBF and Betweenness Centrality	1.7	0.135	0.136	1.1	0.359	0.048				0.0	0.998	0.000	1.7	0.197	0.025	0.2	0.66	0.00

The group effect for local efficiency is displayed in Fig. 5.

*YIS* younger insulin sensitive, *YIR* younger insulin resistant, *OIS* older insulin sensitive, *OIR* older insulin resistant.

## Discussion

A key finding from the current study is that CBF is lower in older than younger adults, and that lower CBF is associated with worse working memory and processing speed. Consistent with our first hypothesis, we found lower CBF in older than younger adults in 95% of brain regions. The percentage of regions showing significant age differences reduced to 85% when lower cortical thickness among the older adults was taken into account. These results confirm research suggesting that the older adult brain has lower blood flow on a per gram tissue basis, particularly in the frontal and temporal regions^17,19,20^, and highlight the importance of adjusting for cortical atrophy when examining age differences in cerebral blood flow. After also adjusting for blood pressure differences, the percentage of regions showing age-related CBF reductions was 36%. These results are consistent with previous research^38–43^ suggesting that elevated blood pressure in ageing is an important contributor to cerebral hypoperfusion. We discuss the possible mechanisms driving these results below.

The results partly support our second hypothesis that higher insulin resistance is associated with lower CBF. Older insulin resistant adults had lower CBF than younger insulin sensitive adults in 40 regions, but CBF was not significantly lower in younger insulin resistant adults. Older insulin resistant adults also had lower absolute CBF than younger insulin resistant adults. Taken together, these results suggest that CBF is reduced in older adults with insulin resistance, even without Type 2 diabetes or clinical levels of insulin resistance. The fact that we found age group differences after adjusting for blood pressure differences suggests that insulin resistance is also exerting an influence on regional cerebral blood flow in ageing that is independent of blood pressure.

The results support our third hypotheses of lower CBF being associated with worse cognitive performance. Lower CBF across the networks, particularly the limbic, default, control and attention networks, was correlated with worse verbal and working memory and slower reaction time in tasks requiring cognitive flexibility and response inhibition. Our results further indicated that age largely mediates the relationship between CBF and cognition, results that are consistent with a large body of research showing that older adults typically show a decline in episodic and working memory and processing speed compared with younger adults (see^44^ for review). Our results are also consistent with studies in which reductions in CBF were associated with declining processing speed in healthy older adults^27^ and impaired memory and executive function in adults with type 2 diabetes^45,46^. They are also consistent with recent work in which we showed that lower rates of network glucose metabolism are associated with slower reaction time and psychomotor speed^28^.

We found support for our fourth hypothesis of altered cerebral blood flow and glucose metabolism associations in older and insulin resistant adults. Older people with insulin resistance had a very different pattern of coupling between CBF and CMR_GLC_ than the insulin sensitive groups, suggesting a failure of the coupling. Older insulin resistant adults showed a pattern of moderate (r > 0.2), positive correlations in 79% of regions. We recently reported the relationship between CMR_GLC_, age and insulin resistance in the same sample used in the current study^28^. Insulin resistance had a small, non-significant negative effect on CMR_GLC_ in older adults. The reason for this pattern of lower regional CBF and CMR_GLC_ but positive associations in older adults with insulin resistance is unclear. The positive associations appear to be an outcome of normal age related hypoperfusion and hypometabolism, as well as an additional insulin resistant-driven reduction in blood flow. Together these results suggest that the positive association in older insulin resistant adults is a response to an “energy crisis” of low absolute levels of blood flow and rates of glucose metabolism. Such an “energy crisis” is possibly an early pathological event in age-related neurodegenerative disease^47^.

The regional associations of CBF and CMR_GLU_ in younger and older insulin sensitive adults showed mostly small-to-moderate negative correlations. These negative correlations may allow for the maintenance of metabolic homeostasis within a relatively tight physiological range. In other words, to maintain the supply of blood carrying O_2_ and glucose, blood flow and glucose metabolic rates increase in compensation for a decrease in the other and vice versa. For example, if cerebral rates of glucose metabolism drop, blood flow is increased to ensure the supply of additional blood carrying metabolic substrates.

The association of CBF and CMR_GLC_ in the insulin resistant younger adults appears to show early signs of changes, although not to the extent seen in older insulin resistant adults (only 25% regions showed an inversion of positive correlations > 0.2, compared to 79% in older adults). These results raise the possibility that for younger insulin resistant adults, progression of their insulin resistance may result in a phenotype resembling older insulin resistant adults, even in mid-life. In other words, insulin resistance may accelerate “normal” age-related reductions in CMR_GLC_ and its coupling with cerebral blood flow. Additional research across the spectrum of insulin resistance and the adult lifespan could test this hypothesis and further characterise the effects.

There were low topological similarities among the four groups in terms of the regions showing the strongest associations between cerebral blood flow and metabolism. The highest 25% of correlations between CBF and CMR_GLC_ among the insulin resistant older adults ranged from 0.62 to 0.88. Fifteen of the 25 regions (60%) were in default, control and salience ventral attention networks, including regions in the prefrontal and medial frontal cortices, the insula and superior parietal lobule. Seven regions (28%) were in the somatomotor cortex. These results suggest that the positive associations in response to cerebral hypoperfusion and hypometabolism in older insulin resistant adults is prioritised to the prefrontal and somatomotor cortices to support sensory and motor processing and executive functions, such as attention, memory and cognitive control^48^. They also raise the possibility that reduced supply of metabolic substrates is contributing to age-related impairments widely reported in executive function^49^.

We found that insulin resistance affects the strength and direction of the associations between cerebral blood flow and local network efficiency. In insulin sensitive individuals, higher CBF was associated with lower local network efficiency, particularly in younger adults. However, the correlation was reduced in strength for older insulin sensitive adults and weakly positive for older insulin resistant adults. Separate areas of research have shown that older age is associated with an increase in insulin resistance^50,51^ and a loss of local network efficiency^52^. This study is the first to examine these effects within the same cohort and the results indicate that age-related increases in insulin resistance likely impact the efficiency of local network communication via cerebral blood flow.

Although we did not directly assess the mechanisms driving CBF alterations in ageing, changes to the physiology of the cerebrovascular in older adults have been well described. For example, age related cerebrovascular damage causes a loss of elasticity and thickening of the arterial walls, impaired cerebrovascular reactivity, damage to the blood brain barrier and ischemia^53–55^. Changes to larger arteries progresses to distal and downstream damage of the microvasculature and the neurovascular unit, which in turn leads to reduced efficiency in neural processing^55^. These changes can also impact cognition due to reduced oxygen and glucose and an impaired ability of the microvasculature to respond to neuronal metabolic demands^56^.

Our results suggest that insulin-dependent signalling contributes to CBF regulation. Age-related decreases of circulating IGF-1 levels have been identified as a mechanism driving age-related declines in CBF^57^, and resistance to IGF-1 in the brain has been linked to neurodegeneration^10^. Insulin resistance has also been associated with endothelial dysfunction, elevated blood pressure, changes in the cerebral arteries and neurovascular coupling (see^58^ for review of mechanisms). Disrupted neurovascular coupling is seen in diabetes and other diseases in which insulin resistance and cardiometabolic dysfunction play a role, such as dementia (see^19,20,59^ for reviews). Mechanistically, insulin resistance and type 2 diabetes have been shown to affect the cells types (e.g., neurons, astrocytes, microglia), pathways and blood flow underpinning neurovascular coupling^55,60,61^. Our findings suggest that alterations to insulin-dependent signalling in the cerebrovascular are not confined to adults who meet clinical thresholds for insulin resistance. Rather, the association of blood flow with both glucose metabolism and local efficiency is affected in insulin resistant adults who have not progressed to a clinical diagnosis. Maintaining insulin sensitivity and cerebrovascular health through a healthy lifestyle could support cognitive ageing. A focus on exercise, sleep, stress and nutrition could lead to improvements in cerebrovascular and metabolic health and cognitive outcomes in ageing^55^.

Our results are consistent with research showing that elevated blood pressure reduces CBF and alters neurovascular coupling^62,63^. Narrowing of major cerebral arteries in the early stages of hypertension may protect downstream vessels from elevated pressure but lead to eventual stenosis of the arteries and decreased downstream blood supply^64^. Hypertension also alters autoregulation of blood flow^65^. We found that elevated systolic blood pressure was more strongly associated with lower CBF than elevated diastolic blood pressure, whereas diastolic blood pressure was more important in CBF and local network efficiency coupling. This later result is consistent with that seen in cortical thickness. In Gutteridge et al., we found that long-term variability in diastolic, and not systolic, blood pressure was an important influence on cortical thickness in older age^66^. Of note, in the current study we obtained a single measure of blood pressure at a single timepoint, whereas in Gutteridge et al. variability in blood pressure over three years was examined. On balance, the evidence indicates that both diastolic and systolic blood pressure are important mediators of cerebral blood flow and brain health in older age^67,68^ and suggest that lifestyle and antihypertensive medications that reduce blood pressure may support cognitive health among ageing adults at risk for hypertension (see^69^, for discussion of previous trials).

The current study used a cross-sectional design, limiting conclusions about the causal relationship between age, insulin resistance and cerebral blood flow. It is also possible that the differences we found between groups reflects unmeasured differences in the cohorts rather than age-related changes. Research measuring changes in CBF and CMR_GLC_ longitudinally could help confirm the causal pathways and the magnitude of yearly or decade-long reductions in CBF and their impact on coupling and cognition. Such research may also help explain the time course and relative impact of mediating factors, such as blood pressure. Research also suggests that CBF alterations across the lifespan may follow a non-linear trajectory. CBF undergoes a rapid drop in the second decade, remains stable until approximately the fifth decade, before gradually declining in older age^70^. Due to the absence of “middle aged” adults in the current study, non-linear relationships could not be tested but could be a topic for future research.

Although our results are informative in terms of the association between CBF and CMR_GLC_ at the regional level and over relatively long timescales, additional research assessing these factors at other spatial and temporal resolutions is needed. In particular, investigating the relationship between insulin resistance, blood flow and glucose metabolism at higher spatial and temporal resolutions could provide important insights into brain health in ageing and disease (see^71^, for a review of methods). Our age and insulin resistance groups also had relatively small sample sizes and our results require replication.

In conclusion, older adults show hypoperfusion and hypometabolism across the cortex. Cerebral blood flow is further reduced in ageing with peripheral insulin resistance and the presence of hypertension. For older insulin resistant adults, the association between cerebral blood flow and metabolism is strongly positive, particularly in the prefrontal and somatomotor cortices. This positive association possibly reflects a response to an under supply of metabolic substrates and an effort to support the brain’s high energy demands. Rates of glucose metabolism and blood flow are higher in younger adults. However, insulin resistance in younger adults reduces cerebral glucose metabolism. The CBF and CMR_GLC_ associations in younger insulin resistant adults shows early signs of inversion to positive correlations, although not yet to the extent seen in older insulin resistant adults. “Normal” age-related changes and/or progression in insulin resistance are likely to further alter blood flow and its association with glucose metabolism and place younger insulin resistant individuals at risk for cognitive decline. Our findings underscore the criticality of insulin sensitivity to brain health across the adult lifespan and the importance of maintaining cerebrovascular and metabolic health to support cognitive function.

## Method

### Study ethics

The study protocol was reviewed and approved by the Monash University Human Research Ethics Committee, in accordance with Australian Code for the Responsible Conduct of Research (2007) and the Australian National Statement on Ethical Conduct in Human Research (2007). Participants provided informed consent to participate in the study. Administration of ionizing radiation was approved by the Monash Health Principal Medical Physicist, following the Australian Radiation Protection and Nuclear Safety Agency Code of Practice (2005). For participants older than 18 years, the annual radiation exposure limit of 5 mSv applies; the effective dose in this study was 4.9 mSv.

### Participants

Ninety participants were recruited from the general community via local advertising. An initial screening interview ensured that participants had the capacity to provide informed consent, did not have a known diagnosis of diabetes, neurological or psychiatric illness, and were not taking psychoactive medication that could affect cognitive function or metabolism. Participants were also screened for claustrophobia, non-MR compatible implants, and clinical or research PET scan in the past 12 months. Women were screened for current or suspected pregnancy. Participants received a $100 voucher for participating in the study.

The final sample included 75 individuals, 34 younger (mean 28.2; SD 6.3; range 20–42 years) and 41 older (mean 75.7; SD 5.8; range 66–86 years) adults (see Table 1). Fifteen participants were excluded from further analyses due to blood haemolysis or well counter issues preventing insulin measurement or kinetic modelling (n = 7), excessive head motion (n = 2) or incomplete PET (n = 2) or ASL scans (n = 2). Four participants were excluded due to consistently high or low ASL or CMR_GLC_ values more than 2.5 standard deviations from the mean.

### Demographic and behavioural measures

Prior to the scan, participants completed an online demographic and lifestyle questionnaire including age, sex, education, height and weight. They also completed a cognitive test battery. Briefly, the following cognitive measure were used (see Supplement for details): Delayed recall and a recognition discrimination index from the Hopkins Verbal Learning Test; length of longest correct series of forward and backward recall from a digit span test to index working memory; the proportion of correct trials and mean reaction time in a task switching test to index cognitive control and flexibility; the probability of responding and reaction time in a stop signal task to measure response inhibition; and number and total correct responses and seconds per correct response in a digit substitution task to measure visuospatial performance and processing speed.

### MR-PET data acquisition

Participants underwent a 90-min simultaneous MR-PET scan in a Siemens (Erlangen) Biograph 3-Tesla molecular MR scanner. Participants were directed to consume a high-protein/low-sugar diet for the 24 h prior to the scan. They were also instructed to fast for six hours and to drink 2–6 glasses of water. Prior to FDG infusion, participants were cannulated in the vein in each forearm and a 10 ml baseline blood sample taken. At the beginning of the scan, half of the 260 MBq FDG tracer was administered via the left forearm as a bolus, providing a strong PET signal from the beginning of the scan. The remaining 130 MBq of the FDG tracer dose was infused at a rate of 36 ml/hour over 50 min, minimising the amount of signal decay over the course of the data acquisition. We have previously demonstrated that this protocol provides a good balance between a fast increase in signal-to-noise ratio at the start of the scan, and maintenance of signal-to-noise ratio over the duration of the scan^72^.

Participants were positioned supine in the scanner bore with their head in a 32-channel radiofrequency head coil and were instructed to lie as still as possible. The scan sequence was as follows. Non-functional MRI scans were acquired during the first 12 min, including a T1 3DMPRAGE (TA = 3.49 min, TR = 1,640 ms, TE = 234 ms, flip angle = 8°, field of view = 256 × 256 mm^2^, voxel size = 1.0 × 1.0 × 1.0 mm^3^, 176 slices, sagittal acquisition) and T2 FLAIR (TA = 5.52 min, TR = 5,000 ms, TE = 396 ms, field of view = 250 × 250 mm^2^, voxel size = 0.5 × 0.5 × 1 mm^3^, 160 slices) to image the anatomical grey and white matter structures, respectively. Thirteen minutes into the scan, list-mode PET (voxel size = 2.3 × 2.3 × 5.0mm^3^) and T2* EPI BOLD-fMRI (TA = 40 min; TR = 1,000 ms, TE = 39 ms, FOV = 210 mm^2^, 2.4 × 2.4 × 2.4 mm^3^ voxels, 64 slices, ascending axial acquisition) sequences were initiated. A 40-min resting-state scan was undertaken in naturalistic viewing conditions watching a movie of a drone flying over the Hawaii Islands. At 53 min, a 5-delay pseudo-continuous arterial spin labelling (pCASL) scan was undertaken. Scan parameters were TR = 4,220 ms; TE = 45.46 ms; FOV = 240 mm; slice thickness = 3 mm; voxel size 2.5 × 2.5 × 3.0 mm^3^. PLDs were 0.5, 1, 1.5, 2, and 2.5 s, duration of the labelling pulse was 1.51 s. At 58 min, diffusion-weighted imaging (DWI) was acquired with 71 directions to index white matter connectivity. DWI results are not reported here.

Plasma radioactivity levels were measured throughout the duration of the scan. Beginning at 10-min post infusion onset, 5 ml blood samples were taken from the right forearm using a vacutainer at 10-min intervals for a total of nine samples. The blood sample were immediately placed in a Heraeus Megafuge 16 centrifuge (ThermoFisher Scientific, Osterode, Germany) and spun at 2,000 rpm (RCF ~ 515 g) for 5 min. 1,000-μL plasma was pipetted, transferred to a counting tube, and placed in a well counter for four minutes. The count start time, total number of counts, and counts per minute were recorded for each sample.

### MRI pre-processing, cerebral blood flow, cortical thickness and graph theory metrics

To quantify the ASL signal, the BASIL toolkit of the Oxford Centre for Functional MRI of the BRAIN (FMRIB)’s software library (FSL) was used (https://fsl.fmrib.ox.ac.uk/fsl/fslwiki/BASIL). A calibration map M0 of proton density weighted image was acquired for each participant. Single-subject whole-brain CBF maps were calculated from perfusions weighted images (direct subtraction of label and control volumes) in BASIL. Processing included motion correction, distortion correction with field map and partial volume correction. The model included a macro vascular component, adaptive spatial regularisation of perfusion, and incorporating T1 uncertainty. The arterial transit time was set at 1.3 s, T1/T1b at 1.3/1.66 s, and inversion efficiency at 0.85. The resulting CBF images in native space were aligned to the anatomical T1w images and normalised to MNI152 space with Advanced Normalization Tools (ANTs).

For the structural T1 images, the brain was extracted in Freesurfer; and the quality of the pial/white matter surface was manually checked and corrected. Corrected Freesurfer surfaces were registered to MNI152 space using ANTs. Cortical thickness for the Schaefer 100 regions were obtained from the Freesurfer reconstruction statistics for each participant. Freesurfer calculates cortical thickness as the closest distance from the grey and white matter boundary to the grey matter and cerebrospinal fluid boundary at each vertex^73,74^.

For the BOLD-fMRI data, T2* images were brain extracted (FSL BET), unwarped and motion corrected with six rotation and translation parameters (FSL MCFLIRT), temporally detrended, normalised to MNI space and smoothed at 8 mm FWHM^75^. Framewise displacement was calculated for each participant to check for excessive head motion (mean and % of frames > 0.3 mm). The pre-processed BOLD timeseries data was loaded to the CONN toolbox^76^ and denoised by regression of white matter and CSF confounds. The timeseries data was bandpass frequency filtered between 0.01 Hz and 0.1 Hz. Regions of interest were generated using the Schaefer 100 parcellations^77^. Graph metrics were derived after thresholding the resulting matrices at the highest 40% of edges to describe the topological properties of the entire network^78^. Global efficiency, local efficiency and betweenness centrality were used, as follows:

**Global Efficiency** at a node is defined as the average of the shortest inverse-distances between the node and all other nodes in the graph. Across the entire graph, global efficiency represents a measure of global integration.

**Local Efficiency** at each node is defined as the average of shortest inverse-distances between the nodes within the neighbouring sub-graph (all nodes neighbouring that node and all existing edges among them). Network local efficiency is a measure of local integration of a network.

**Betweenness Centrality** is defined as the proportion of times that a node is part of a shortest-path between any two pairs of nodes within a graph. It represents an alternative measure of node centrality within a graph.

### PET image reconstruction, pre-processing and CMR_GLC_ calculations

The list-mode PET data for each subject was binned into 344 3D sinogram frames of 16 s intervals. Attenuation was corrected via the pseudo-CT method for hybrid PET-MR scanners^79^ Ordinary Poisson-Ordered Subset Expectation Maximization algorithm (3 iterations, 21 subsets) with point spread function correction was used to reconstruct 3D volumes from the sinogram frames. The reconstructed DICOM slices were converted to NIFTI format with size 344 × 344 × 127 (voxel size: 2.3 × 2.3 × 5.0mm^3^) for each volume. All 3D volumes were temporally concatenated to form a single 4D NIFTI volume. After concatenation, the PET volumes were motion corrected using FSL MCFLIRT^75^, with the mean PET image used to mask the 4D data. PET images were corrected for partial volume effects using the modified Müller-Gartner method.

PET images were corrected for partial volume effects using the modified Müller-Gartner method implemented in PetSurf (https://surfer.nmr.mgh.harvard.edu/fswiki/PetSurfer). The method corrects for white matter spill in and grey matter spill out of the PET signal^80,81^. A grey matter binary mask with a threshold of 25% was used^80,81^. We also used a Gaussian kernel with a full width at half maximum of 12 mm to increase the signal-to-noise ratio. Subcortical structures were partial volume corrected and spatially smoothed in volume space and merged with the cortical data.

Calculation of CMR_GLC_ was undertaken in PMOD 4.4 (http://www.pmod.com) using the FDG time activity curves for the Schaefer 100 parcellation and major subcortical structures from the AAL atlas. The FDG in the plasma samples was decay-corrected for the time between sampling and counting and used as the input function to the Patlak models. A lumped constant of 0.89 was used, and equilibrium (t) set at 10 min, the time corresponding to the peak of the bolus and onset of a stable signal. The fractional blood space (vB) was set at 0.05^82^. Participant’s plasma glucose (mmol) was entered in the model from their baseline blood sample.

### Insulin resistance

The baseline blood sample was used to collect 2 ml of plasma for insulin and glucose measurement, which was undertaken by a commercial laboratory. HOMA-IR was calculated as fasting glucose (mmol/L) × fasting insulin (mU/L)/22.5^83^. The constant of 22.5 is a normalising factor for normal fasting plasma insulin and glucose (i.e., 4.5 mmol/L × 5 mU/L = 22.5). Higher HOMA-IR values indicate greater insulin resistance. We also calculated HOMA-IR2 using the calculator at https://www.rdm.ox.ac.uk/.

### Data analysis

All analyses were run in SPSS version 29.0 (https://www.ibm.com/products/spss-statistics).

#### Demographics and cognitive measures

For the demographic variables, independent T-tests for age group differences in continuous and Chi-square tests for categorical variables were undertaken and tested at uncorrected *p* < 0.5. Four groups were created based on age category (younger and older) and a HOMA-IR median split (low and high HOMA-IR, or insulin sensitive and insulin resistant) and differences in the demographics variables among the groups were tested using general linear models (GLMs) at uncorrected *p* < 0.05.

#### The effect of age, cortical thickness and blood pressure on regional CBF

Three series of GLMs was run to assess whether regional CBF was lower in older versus younger adults and whether the effect of age group was mediated by differences in cortical thickness and blood pressure. In the first series of GLMs, the association between regional CBF and age group was tested. In the second series, cortical thickness was included in the models as a covariate; in the third series, cortical thickness and systolic and diastolic blood pressure were also added. Each series of GLMs was FDR-corrected at *p* < 0.05^84^.

#### The effect of age and HOMA-IR on regional CBF

Differences in regional CBF were compared among the four groups based on age category and HOMA-IR median split in a series of GLMs. Regional cortical thickness and systolic and diastolic blood pressure were included as covariates. For regions showing significant group differences, post-hoc contrasts were run comparing the younger insulin sensitive group to the other three groups. A similar series of analyses were run for four groups based on age category and HOMA-IR2 median split. Each series of GLMs was FDR-corrected at *p* < 0.05.

#### Association between CBF and cognition

To test our hypothesis that lower CBF will be associated worse cognitive performance, we ran a series of canonical correlation analyses. A canonical correlation analysis tests the relationships between two multivariate sets of latent variables and identifies a mode as the source of common statistical associations in the variable sets. The mode represents the linear combinations of the components in each set that maximally covary across subjects, termed canonical variates. The number of potential canonical variates reflects the smallest number of variables in either set, in this case the 10 cognitive measures.

Stop signal reaction time, seconds per correct response in the digit substitution and category switch reaction time were multiplied by − 1 so that higher scores reflect better performance. The cognitive scores were then converted to Z-scores. Network CBF were included to return a cognitive profile associated with network CBF. The F-statistic from the Wilk’s test was used to test the null hypothesis that the canonical correlation and all smaller ones are equal to zero.

To assess whether age group, blood pressure and cortical thickness mediates the relationship between cognition and network CBF, we repeated the canonical correlation analyses. First, age group, diastolic and systolic blood pressure and whole brain cortical thickness were regressed onto each network CBF measure and the residuals saved for each participant. Canonical correlation analysis was then undertaken between the cognition measures and the residuals of network CBF.

#### Coupling of regional and network CBF and CMR_GLC_

Differences in CBF and CMR_GLC_ coupling were compared among the four groups based on age category and levels of insulin resistance. A separate series of partial correlations was calculated at the seven network and 100 region levels for each of the four groups, controlling for cortical thickness at the same level as well as systolic and diastolic blood pressure. At the region level, Pearson correlations, Spearman correlations and DICE coefficients were calculated comparing the strength, rank and spatial similarity of the coupling for the younger insulin sensitive group to each of the other three groups.

#### Effect of age and HOMA-IR on the coupling of CBF and functional brain network properties

For each participant, a Pearson’s correlation was computed between CBF and global and local efficiency and betweenness centrality across the 100 regions. The correlations were considered a measure of coupling of CBF and functional network properties and used as the dependent variables in separate GLMs. Differences between the four groups based on age category and insulin resistance levels were tested, together with whole brain cortical thickness and systolic and diastolic blood pressure as covariates.

### Supplementary Information

Supplementary Information.

## Data Availability

The datasets used and/or analysed during the current study available from the corresponding author on reasonable request.
