# Supplementary material for: The association of regional cerebral blood flow and glucose metabolism in normative ageing and insulin resistance
Source: Sci Rep. 2024 Jun 25;14:14574. doi: 10.1038/s41598-024-65396-4 (PMC11196590; doi:10.1038/s41598-024-65396-4)
Supplement: Supplementary file 1 — Supplementary Information. [file 41598_2024_65396_MOESM1_ESM.pdf]

The association of regional cerebral blood flow and glucose metabolism in normative ageing and insulin resistance

Deery, Liang, Di Paolo, Voigt, Murray, Siddiqui, Egan, Moran, Jamadar

~ Supplementary Information ~

Table of Contents

1. Supplementary Methods 3
2. Supplementary Results 5
2.1 The Effect of Age, Cortical Thickness and Blood Pressure on Regional CBF 6
2.2 The Effect of Age and Insulin Resistance on Regional CBF 9
2.3 The Effect of Age and Insulin Resistance on Regional CBF-CMR_{GLU} Associations 14
2.4 The Effect of Other Demographic Variables on CBF and CBF-CMR_{GLU} Association 18
3. Supplementary References 23

1. Supplementary Methods

Demographic and Cognitive Battery

Prior to the scan, participants completed an online demographic and lifestyle questionnaire including age, sex, education, height and weight, history of smoking, alcohol and recreational drug use. Participants also completed a cognitive test battery consisting of measures of general intelligence, working memory, cognitive flexibility, inhibitory control and verbal learning.

Wechsler Abbreviated Scale of Intelligence (WASI-IQ). An assessment of intelligence suitable for ages 6-90 years [1]. There are 4 subtests: block design, vocabulary, matrix reasoning and similarities. WASI-IQ was scored by converting raw scores into a scale score, which were transformed into a composite score reflecting verbal comprehension and perceptual reasoning abilities (FSIQ2). This score was converted to an age-based T scores established in a normal population.

Hopkins Verbal Learning Test (HVLT). A three-trial list learning and free recall task comprising 12 words, four words from each of three semantic categories [2]. Approximately 20–25 minutes later, a delayed recall trial and a recognition trial was completed. The delayed recall required free recall of any words remembered. The recognition trial comprised 24 words, including the 12 target words and 12 false-positives, six semantically related, and six semantically unrelated. Delayed recall (total words recalled) and a recognition discrimination index (number of correct minus number of false positives in the recognition task) were calculated.

Digit Span. A measure of verbal short term and working memory used in two formats: Forward and backward digit span [3]. Participants were presented with a series of digits, and are asked to repeat them in either the order presented (forward span) or in reverse order (backwards span). After two consecutive failures of the same length, the test was stopped. Scores were derived as the length of longest correct series for both forward and backward recall.

Task Switching. A computer-based test in which participants were given a word and had to perform one of two simple categorisation tasks, depending on the cue that appeared with the word: 1) 'living' task. If the cue was a heart, participants were asked to categorise the word via a key press based on whether it represents a LIVING versus a NON-LIVING object; and 2) 'size' task. If the cue was an arrow-cross, participants were asked to categorise the word via a key press based on whether it represents an object that is BIGGER or SMALLER than a basketball. The cue selection for each new trial was randomised. Half the test trials were switch trials; half non-switch trials. Half the switch and non-switch trials were congruent in the key presses for either task, half were incongruent. The measures used included the mean latency of correctly responding to a switch trial and switch cost. Switch cost is the difference between mean correct latency of switch trials and nonswitch trials with positive value indicating participants were slower on switch trials, that is, there was a latency cost to switching [4].

Stop Signal. A computer-based test in which participants were presented an arrow that pointed either right or left [5]. The task was to press the left response key if the arrow pointed to the left and press the right response key if the arrow pointed to the right, unless a signal beep is played after the presentation of the arrow. In this case the response should be stopped before execution. The delay between presentation of arrow and signal beep (starting at 250ms) was adjusted up or down (by 50ms) depending on performance. The delay got longer if the previous signal stop was successful (up to 1150ms) and smaller if the previous signal stop was not successful (down to 50ms). The stimulus onset asynchrony between the start of each trial (onset of fixation circles) was 2000ms. Variables were the mean reaction time in stop signal trials and stop signal reaction time. Stop signal reaction time is an estimate of inhibition ability, that is, the time required to stop the initiated go-process. The slower the stop signal reaction time, the more difficult to stop the go-process.

Digit Symbol Substitution. A computer-based task in which participant were presented with an 18 columns x 16 rows matrix [6]. The task was to translate symbols shown above the matrix (key) into digits in the matrix within a two minute period. Total count of correct responses and seconds per correct response were recorded.

2. Supplementary Results

2.1 The Effect of Age, Cortical Thickness and Blood Pressure on Regional CBF

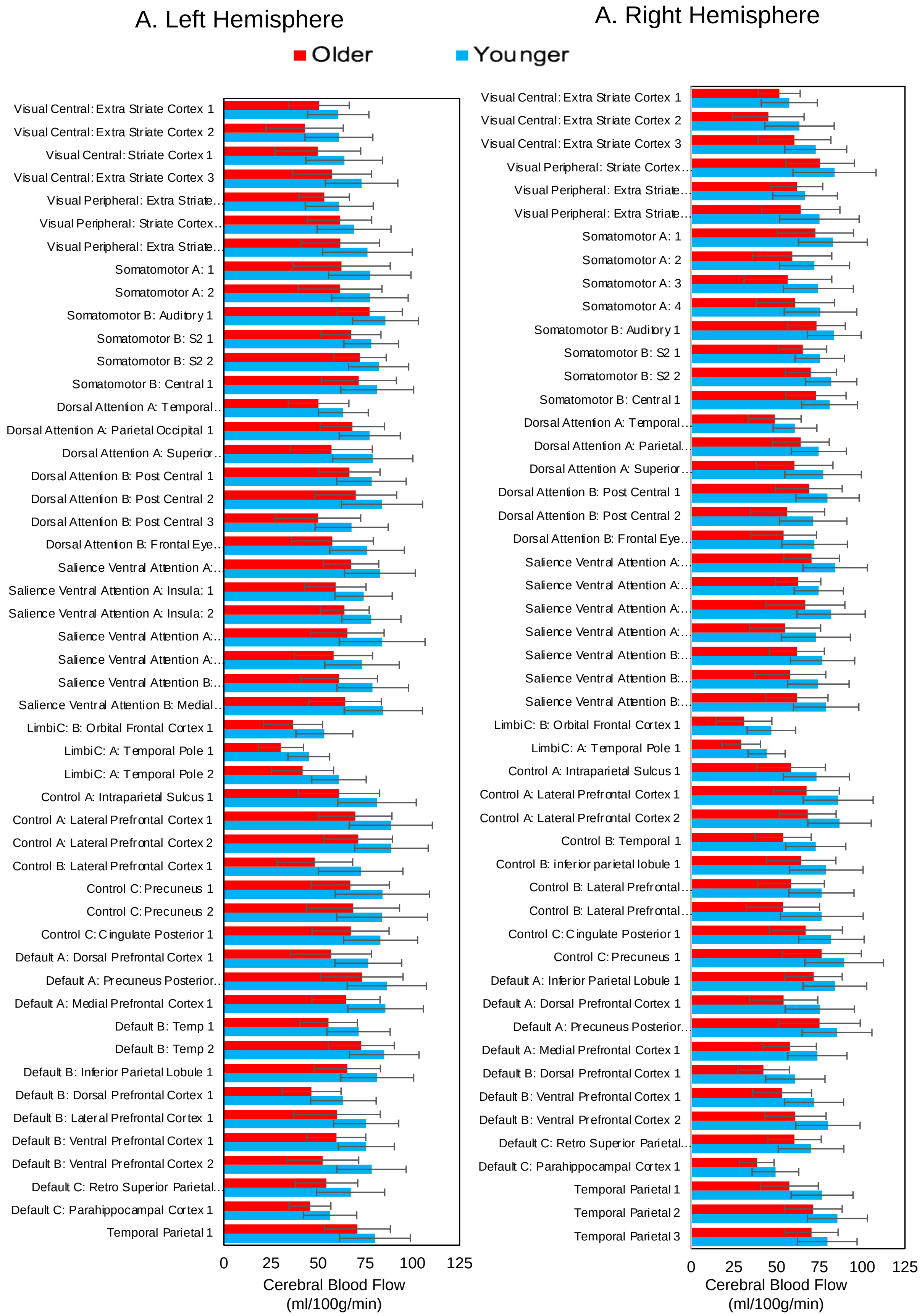

Figure S1. Mean and standard deviation (error bars) of regional cerebral blood flow for younger and older adults. The data are also presented in Table S1.

Table S1. Mean and standard deviation (SD) of regional cerebral blood flow (ml/100g/min) for younger and older adults. The data are also shown in Figure S1.

Left Hemisphere					Right Hemisphere				
	Younger		Older			Younger		Older	
	Mean	SD	Mean	SD		Mean	SD	Mean	SD
Visual Central: Extra Striate Cortex 1	60.7	16.3	50.5	16.1	Visual Central: Extra Striate Cortex 1	57.4	16.4	51.5	12.2
Visual Central: Extra Striate Cortex 2	61.0	18.0	42.8	20.5	Visual Central: Extra Striate Cortex 2	63.4	20.3	45.2	20.8
Visual Central: Striate Cortex 1	63.9	20.3	49.8	22.7	Visual Central: Extra Striate Cortex 3	72.9	18.1	60.5	21.2
Visual Central: Extra Striate Cortex 3	73.1	19.2	57.2	21.0	Visual Peripheral: Striate Cortex Calcarine 1	83.8	24.3	75.4	20.0
Visual Peripheral: Extra Striate Inferior 1	61.2	18.0	53.2	13.4	Visual Peripheral: Extra Striate Inferior 1	66.6	18.9	61.8	15.2
Visual Peripheral: Striate Cortex Calcarine 1	69.1	19.6	61.4	17.0	Visual Peripheral: Extra Striate Superior 1	75.0	23.2	64.1	22.9
Visual Peripheral: Extra Striate Cortex Sup 1	76.1	23.8	61.7	20.8					
Somatomotor A: 1	77.4	21.9	62.4	25.8	Somatomotor A: 1	82.8	20.1	72.7	22.2
Somatomotor A: 2	77.4	20.3	61.6	22.3	Somatomotor A: 2	72.2	20.5	59.1	23.1
Somatomotor B: Auditory 1	85.7	17.5	77.4	17.2	Somatomotor A: 3	74.4	20.4	56.6	25.6
Somatomotor B: S2 1	78.2	14.5	67.5	15.8	Somatomotor A: 4	75.6	21.2	60.9	22.9
Somatomotor B: S2 2	82.0	16.0	72.1	14.0	Somatomotor B: Auditory 1	83.7	15.7	73.2	16.8
Somatomotor B: Central 1	81.3	19.3	71.5	19.9	Somatomotor B: S2 1	75.2	14.5	65.2	14.1
					Somatomotor B: S2 2	81.8	15.0	70.0	15.0
					Somatomotor B: Central 1	80.9	16.3	73.0	17.6
Dorsal Attention A: Temporal Occipital 1	63.4	13.2	50.2	16.1	Dorsal Attention A: Temporal Occipital 1	60.7	12.8	48.8	15.5
Dorsal Attention A: Parietal Occipital 1	77.4	16.2	68.1	17.1	Dorsal Attention A: Parietal Occipital 1	74.7	16.1	64.2	16.6
Dorsal Attention A: Superior Parietal Lobule 1	79.0	21.3	57.1	21.7	Dorsal Attention A: Superior Parietal Lobule 1	77.2	22.4	60.5	22.5
Dorsal Attention B: Post Central 1	78.3	18.4	66.5	16.3	Dorsal Attention B: Post Central 1	79.7	18.5	68.9	19.4
Dorsal Attention B: Post Central 2	83.9	21.5	70.0	21.6	Dorsal Attention B: Post Central 2	71.4	19.6	56.3	21.7
Dorsal Attention B: Post Central 3	67.7	19.4	49.9	22.7	Dorsal Attention B: Frontal Eye Fields 1	72.2	19.2	54.1	19.2
Dorsal Attention B: Frontal Eye Fields 1	76.0	19.8	57.5	21.7	Salience Ventral Attention A: Parietal Operculum 1	84.3	18.8	70.5	16.3
Salience Ventral Attention A: Parietal Operculum 1	82.7	18.8	67.5	14.6	Salience Ventral Attention A: Insula: 1	74.7	14.4	62.5	13.3
Salience Ventral Attention A: Insula: 1	74.1	15.2	59.2	16.2	Salience Ventral Attention A: Parietal Medial 1	81.8	19.9	66.8	23.0
Salience Ventral Attention A: Insula: 2	78.2	15.7	64.0	13.0	Salience Ventral Attention A: Frontal Medial 1	73.0	20.1	54.9	20.9
Salience Ventral Attention A: Parietal Medial 1	84.0	22.8	65.4	19.5	Salience Ventral Attention B: Inferior Parietal Lobule 1	76.8	18.8	61.7	16.1
Salience Ventral Attention A: Frontal Medial 1	73.3	19.7	58.1	20.8	Salience Ventral Attention B: Lateral Prefrontal Cortex 1	74.4	18.0	58.0	20.7
Salience Ventral Attention B: Lateral Prefrontal Cortex 1	78.9	18.9	61.2	20.3	Salience Ventral Attention B: Medial Posterior Prefrontal 1	79.0	19.1	61.7	18.3
Salience Ventral Attention B: Medial Posterior Prefrontal 1	84.6	20.7	64.3	19.3					
Limbic B: Orbital Frontal Cortex 1	53.3	15.1	36.6	15.7	Limbic B: Orbital Frontal Cortex 1	46.9	14.2	30.9	16.3
Limbic A: Temporal Pole 1	45.0	11.1	30.2	12.0	Limbic A: Temporal Pole 1	44.1	10.8	29.2	11.3
Limbic A: Temporal Pole 2	61.0	14.4	41.7	16.5					
Control A: Intraparietal Sulcus 1	81.2	20.8	61.0	21.6	Control A: Intraparietal Sulcus 1	73.2	19.4	58.3	20.1
Control A: Lateral Prefrontal Cortex 1	88.5	22.1	69.7	19.5	Control A: Lateral Prefrontal Cortex 1	86.0	20.5	67.4	19.2
Control A: Lateral Prefrontal Cortex 2	88.9	19.4	71.2	18.2	Control A: Lateral Prefrontal Cortex 2	86.8	18.5	68.1	16.6
Control B: Lateral Prefrontal Cortex 1	72.5	22.4	48.2	20.1	Control B: Temporal 1	72.9	17.5	53.8	16.4
Control C: Precuneus 1	84.1	25.1	67.0	20.8	Control B: inferior parietal lobule 1	79.0	21.5	64.4	20.2
Control C: Precuneus 2	83.9	24.1	68.6	24.6	Control B: Lateral Prefrontal Cortexd 1	76.2	19.1	58.4	19.5
Control C: Cingulate Posterior 1	83.1	19.6	67.2	20.4	Control B: Lateral Prefrontal Cortexv 1	76.4	24.2	53.7	21.4
					Control C: Cingulate Posterior 1	82.0	19.1	67.1	21.4
					Control C: Precuneus 1	89.4	23.0	76.4	23.1
Default A: Dorsal Prefrontal Cortex 1	76.6	17.7	56.9	21.4	Default A: Inferior Parietal Lobule 1	84.0	18.6	71.5	16.7
Default A: Precuneus Posterior Cingulate Cortex1	86.4	21.0	73.2	21.9	Default A: Dorsal Prefrontal Cortex 1	75.2	20.2	54.1	20.0
Default A: Medial Prefrontal Cortex 1	85.8	20.0	64.7	18.1	Default A: Precuneus Posterior Cingulate Cortex 1	85.3	20.4	75.2	23.7
Default B: Temp 1	71.4	16.8	55.6	15.3	Default A: Medial Prefrontal Cortex 1	73.8	17.3	57.7	15.6
Default B: Temp 2	85.1	18.4	72.9	17.5	Default B: Dorsal Prefrontal Cortex 1	61.0	17.3	42.4	15.2
Default B: Inferior Parietal Lobule 1	81.3	19.3	65.5	17.6	Default B: Ventral Prefrontal Cortex 1	71.8	17.3	53.3	17.2
Default B: Dorsal Prefrontal Cortex 1	63.4	17.3	46.5	15.7	Default B: Ventral Prefrontal Cortex 2	80.0	18.7	61.0	17.9
Default B: Lateral Prefrontal Cortex 1	75.5	17.3	60.0	22.9	Default C: Retro Superior Parietal Lobuleenial 1	70.1	19.2	60.4	15.6
Default B: Ventral Prefrontal Cortex 1	75.5	14.9	59.7	15.6	Default C: Parahippocampal Cortex 1	49.3	13.6	38.4	10.1
Default B: Ventral Prefrontal Cortex 2	78.3	18.4	52.4	19.1					
Default C: Retro Superior Parietal Lobuleenial 1	67.2	18.1	54.4	16.6					
Default C: Parahippocampal Cortex 1	56.4	14.1	45.7	11.1					
Temporal Parietal 1	80.2	18.8	70.8	17.5	Temporal Parietal 1	76.6	18.1	57.4	16.9
					Temporal Parietal 2	85.6	17.5	71.5	16.8
					Temporal Parietal 3	79.6	17.4	70.5	15.4

Table S2. General liner models of the association of regional cerebral blood flow with age category (model 1), age category and cortical thickness (model 2) and age category, cortical thickness and blood pressure (model 3). The age category effect sizes from each model are plotted on the brain surface in Figure 1 in main document.

	Model 1: Age Category			Model 2: Age Category and Cortical Thickness						Model 3: Age Category, Cortical Thickness, Systolic and Diastolic Blood Pressure					
	Age Category			Overall Model			Age Category			Overall Model			Age Category		
	F	p-FDR	η^2_p	F	p	η^2_p	F	p	η^2_p	F	p	η^2_p	F	p	η^2_p
Visual Central: Extra Striate Cortex 1	7.3	0.011	0.093	3.6	0.032	0.093	6.4	0.013	0.083	2.9	0.030	0.142	2.2	0.139	0.031
Visual Central: Extra Striate Cortex 2	16.1	0.000	0.183	8.1	0.001	0.186	14.8	0.000	0.173	7.3	0.000	0.297	3.8	0.056	0.052
Visual Central: Striate Cortex 1	7.8	0.009	0.097	5.0	0.009	0.123	8.5	0.005	0.107	4.3	0.004	0.199	1.5	0.232	0.021
Visual Central: Extra Striate Cortex 3	11.2	0.002	0.135	5.6	0.006	0.136	8.8	0.004	0.111	4.2	0.004	0.195	2.1	0.152	0.030
Visual Peripheral: Extra Striate Inferior 1	4.7	0.036	0.062	2.5	0.088	0.066	4.9	0.030	0.064	1.5	0.216	0.079	1.7	0.197	0.024
Visual Peripheral: Striate Cortex Calcarine 1	3.3	0.078	0.043	1.7	0.198	0.045	3.2	0.078	0.043	1.4	0.243	0.075	0.5	0.504	0.006
Visual Peripheral: Extra Striate Cortex Sup 1	7.7	0.009	0.097	3.8	0.026	0.097	5.9	0.017	0.077	2.5	0.050	0.127	1.2	0.274	0.017
Somatomotor A: 1	7.1	0.012	0.090	3.7	0.030	0.094	4.8	0.032	0.063	2.2	0.077	0.113	2.1	0.151	0.030
Somatomotor A: 2	10.1	0.004	0.123	5.1	0.009	0.125	5.2	0.025	0.068	2.6	0.046	0.129	3.3	0.073	0.046
Somatomotor B: Auditory 1	4.3	0.046	0.056	2.1	0.125	0.057	2.3	0.138	0.031	1.5	0.202	0.082	0.5	0.497	0.007
Somatomotor B: S2 1	9.0	0.005	0.112	4.5	0.015	0.112	6.2	0.015	0.080	3.3	0.016	0.160	1.1	0.288	0.016
Somatomotor B: S2 2	8.2	0.008	0.102	4.3	0.018	0.107	7.1	0.009	0.091	2.6	0.042	0.132	2.3	0.133	0.032
Somatomotor B: Central 1	4.5	0.040	0.059	2.2	0.113	0.059	3.7	0.059	0.049	1.8	0.131	0.096	0.6	0.445	0.008
Dorsal Attention A: Temporal Occipital 1	14.5	0.001	0.168	7.2	0.001	0.169	12.1	0.001	0.146	5.2	0.001	0.231	4.3	0.042	0.058
Dorsal Attention A: Parietal Occipital 1	5.6	0.023	0.073	3.3	0.045	0.084	6.4	0.014	0.083	3.3	0.016	0.160	1.3	0.258	0.018
Dorsal Attention A: Superior Parietal Lobule 1	19.1	0.000	0.210	10.5	0.000	0.228	19.2	0.000	0.213	6.6	0.000	0.276	7.9	0.006	0.103
Dorsal Attention B: Post Central 1	8.5	0.007	0.105	4.2	0.019	0.106	6.1	0.016	0.079	2.3	0.065	0.119	2.5	0.122	0.034
Dorsal Attention B: Post Central 2	7.5	0.010	0.095	4.6	0.013	0.115	9.1	0.004	0.113	2.8	0.030	0.142	4.7	0.033	0.064
Dorsal Attention B: Post Central 3	13.0	0.001	0.153	7.9	0.001	0.182	15.8	0.000	0.182	4.5	0.003	0.207	6.8	0.011	0.089
Dorsal Attention B: Frontal Eye Fields 1	14.3	0.001	0.166	8.2	0.001	0.188	4.7	0.034	0.062	4.4	0.003	0.203	2.4	0.130	0.033
Salience Ventral Attention A: Parietal Operculum 1	15.3	0.001	0.175	7.7	0.001	0.178	8.6	0.005	0.108	4.8	0.002	0.217	4.4	0.040	0.060
Salience Ventral Attention A: Insula: 1	16.5	0.000	0.186	9.2	0.000	0.206	8.0	0.006	0.102	4.8	0.002	0.217	4.1	0.047	0.056
Salience Ventral Attention A: Insula: 2	18.1	0.000	0.201	9.2	0.000	0.205	13.4	0.000	0.159	5.2	0.001	0.233	6.4	0.014	0.085
Salience Ventral Attention A: Parietal Medial 1	14.2	0.001	0.165	8.0	0.001	0.184	14.3	0.000	0.168	4.8	0.002	0.219	6.5	0.013	0.086
Salience Ventral Attention A: Frontal Medial 1	10.3	0.004	0.125	6.2	0.003	0.148	1.6	0.212	0.022	3.1	0.020	0.154	1.0	0.315	0.015
Salience Ventral Attention B: Lateral Prefrontal Cortex 1	14.9	0.001	0.172	7.4	0.001	0.172	10.2	0.002	0.126	4.8	0.002	0.218	3.6	0.062	0.050
Salience Ventral Attention B: Medial Posterior Prefrontal 1	19.0	0.000	0.209	10.6	0.000	0.230	17.4	0.000	0.196	5.3	0.001	0.233	10.5	0.002	0.132
LimbiC: B: Orbital Frontal Cortex 1	21.4	0.000	0.229	13.9	0.000	0.281	27.1	0.000	0.276	8.7	0.000	0.336	9.3	0.003	0.119
LimbiC: A: Temporal Pole 1	30.0	0.000	0.294	17.8	0.000	0.334	26.6	0.000	0.273	11.5	0.000	0.399	9.1	0.004	0.117
LimbiC: A: Temporal Pole 2	28.2	0.000	0.282	14.2	0.000	0.285	26.1	0.000	0.269	8.8	0.000	0.337	13.6	0.000	0.165
Control A: Intraparietal Sulcus 1	16.6	0.000	0.188	8.4	0.001	0.191	13.1	0.001	0.155	5.2	0.001	0.232	5.8	0.019	0.077
Control A: Lateral Prefrontal Cortex 1	15.2	0.001	0.175	7.8	0.001	0.180	12.3	0.001	0.148	5.0	0.001	0.223	4.7	0.034	0.063
Control A: Lateral Prefrontal Cortex 2	16.4	0.000	0.186	8.2	0.001	0.188	10.4	0.002	0.128	5.5	0.001	0.240	4.5	0.037	0.061
Control B: Lateral Prefrontal Cortex 1	24.2	0.000	0.252	12.1	0.000	0.254	22.9	0.000	0.244	9.4	0.000	0.353	6.0	0.017	0.080
Control C: Precuneus 1	10.2	0.004	0.124	5.9	0.004	0.143	11.6	0.001	0.140	3.6	0.010	0.173	4.7	0.033	0.064
Control C: Precuneus 2	7.3	0.011	0.092	4.1	0.022	0.103	7.9	0.006	0.100	2.2	0.074	0.115	3.6	0.062	0.050
Control C: Cingulate Posterior 1	11.7	0.002	0.139	6.3	0.003	0.152	10.8	0.002	0.132	4.0	0.005	0.190	3.8	0.054	0.053
Default A: Dorsal Prefrontal Cortex 1	18.3	0.000	0.203	9.0	0.000	0.203	12.3	0.001	0.148	5.2	0.001	0.231	5.4	0.023	0.073
Default A: Precuneus Posterior Cingulate Cortex1	7.0	0.012	0.088	3.4	0.037	0.088	4.6	0.036	0.060	2.3	0.066	0.118	1.2	0.269	0.018
Default A: Medial Prefrontal Cortex 1	22.6	0.000	0.239	11.5	0.000	0.245	19.7	0.000	0.217	6.2	0.000	0.263	8.4	0.005	0.108
Default B: Temp 1	18.1	0.000	0.201	10.1	0.000	0.221	6.1	0.016	0.079	6.0	0.000	0.259	1.9	0.172	0.027
Default B: Temp 2	8.5	0.007	0.106	5.0	0.009	0.124	9.5	0.003	0.118	3.2	0.019	0.155	3.6	0.061	0.050
Default B: Inferior Parietal Lobule 1	13.7	0.001	0.160	6.8	0.002	0.161	5.6	0.020	0.074	5.1	0.001	0.228	1.9	0.172	0.027
Default B: Dorsal Prefrontal Cortex 1	19.3	0.000	0.212	9.9	0.000	0.219	5.5	0.022	0.072	5.4	0.001	0.240	2.6	0.110	0.037
Default B: Lateral Prefrontal Cortex 1	10.4	0.003	0.126	5.3	0.007	0.129	8.4	0.005	0.106	3.5	0.011	0.169	3.0	0.086	0.042
Default B: Ventral Prefrontal Cortex 1	19.8	0.000	0.216	11.1	0.000	0.239	21.4	0.000	0.232	5.6	0.001	0.246	11.1	0.001	0.138
Default B: Ventral Prefrontal Cortex 2	34.9	0.000	0.327	17.2	0.000	0.327	21.6	0.000	0.234	10.0	0.000	0.367	9.3	0.003	0.118
Default C: Retro Superior Parietal Lobuleenial 1	10.1	0.004	0.123	5.5	0.006	0.134	6.4	0.014	0.083	4.3	0.003	0.201	1.1	0.295	0.016
Default C: Parahippocampal Cortex 1	13.3	0.001	0.156	7.0	0.002	0.165	10.3	0.002	0.126	3.6	0.010	0.174	5.2	0.026	0.070
Temporal Parietal 1	4.9	0.034	0.064	2.5	0.087	0.066	4.3	0.043	0.057	1.8	0.147	0.093	1.5	0.224	0.021

	Model 1: Age Category			Model 2: Age Category and Cortical Thickness						Model 3: Age Category, Cortical Thickness, Systolic and Diastolic Blood Pressure					
	Age Category			Overall Model			Age Category			Overall Model			Age Category		
Visual Central: Extra Striate Cortex 1	3.1	0.084	0.041	1.5	0.223	0.041	2.3	0.134	0.031	0.9	0.465	0.050	0.7	0.422	0.009
Visual Central: Extra Striate Cortex 2	14.4	0.001	0.166	7.2	0.001	0.168	13.0	0.001	0.155	4.6	0.002	0.211	4.8	0.032	0.065
Visual Central: Extra Striate Cortex 3	7.2	0.011	0.091	3.7	0.028	0.095	6.8	0.011	0.088	3.5	0.012	0.168	1.5	0.220	0.022
Visual Peripheral: Striate Cortex Calcarine 1	2.7	0.107	0.036	1.4	0.244	0.039	2.4	0.128	0.032	1.1	0.345	0.062	0.3	0.578	0.004
Visual Peripheral: Extra Striate Inferior 1	1.5	0.226	0.020	0.8	0.473	0.021	0.8	0.367	0.011	0.8	0.506	0.046	0.0	0.918	0.000
Visual Peripheral: Extra Striate Superior 1	4.1	0.050	0.054	2.2	0.114	0.059	4.4	0.040	0.058	1.6	0.176	0.086	1.3	0.261	0.018
Somatomotor A: 1	4.2	0.048	0.055	3.7	0.029	0.095	1.2	0.272	0.017	2.2	0.075	0.114	0.1	0.700	0.002
Somatomotor A: 2	6.4	0.016	0.082	3.9	0.026	0.098	4.1	0.047	0.055	2.3	0.072	0.116	1.6	0.213	0.022
Somatomotor A: 3	10.7	0.003	0.129	5.4	0.007	0.132	7.4	0.008	0.095	3.2	0.017	0.158	2.4	0.126	0.034
Somatomotor A: 4	8.1	0.008	0.101	4.7	0.012	0.117	2.8	0.098	0.038	2.6	0.042	0.132	1.4	0.246	0.019
Somatomotor B: Auditory 1	7.5	0.010	0.094	3.9	0.024	0.100	2.5	0.118	0.034	2.9	0.029	0.143	0.2	0.642	0.003
Somatomotor B: S2 1	9.1	0.005	0.113	4.5	0.014	0.113	7.3	0.009	0.093	3.0	0.024	0.149	1.4	0.247	0.019
Somatomotor B: S2 2	11.5	0.002	0.138	7.1	0.002	0.167	3.9	0.052	0.052	4.7	0.002	0.214	0.3	0.560	0.005
Somatomotor B: Central 1	4.0	0.052	0.053	2.3	0.105	0.061	1.7	0.200	0.023	1.9	0.121	0.099	0.1	0.715	0.002
Dorsal Attention A: Temporal Occipital 1	12.8	0.003	0.151	6.3	0.003	0.151	10.8	0.002	0.132	4.7	0.002	0.215	4.1	0.047	0.056
Dorsal Attention A: Parietal Occipital 1	7.6	0.010	0.096	3.8	0.028	0.096	5.2	0.026	0.068	2.8	0.032	0.140	1.5	0.218	0.022
Dorsal Attention A: Superior Parietal Lobule 1	10.1	0.004	0.123	5.7	0.005	0.138	11.1	0.001	0.135	3.7	0.009	0.176	4.3	0.042	0.059
Dorsal Attention B: Post Central 1	6.0	0.020	0.077	3.4	0.040	0.087	5.6	0.020	0.074	2.9	0.028	0.144	1.9	0.169	0.027
Dorsal Attention B: Post Central 2	9.7	0.004	0.119	5.4	0.007	0.131	10.4	0.002	0.128	3.2	0.019	0.155	4.1	0.047	0.056
Dorsal Attention B: Frontal Eye Fields 1	16.4	0.000	0.186	8.5	0.001	0.192	8.7	0.004	0.109	4.3	0.004	0.200	5.3	0.024	0.072
Salience Ventral Attention A: Parietal Operculum 1	11.4	0.002	0.137	6.2	0.003	0.149	6.0	0.017	0.078	4.2	0.004	0.197	1.1	0.296	0.016
Salience Ventral Attention A: Insula: 1	14.3	0.001	0.165	7.2	0.001	0.169	6.3	0.014	0.081	4.4	0.003	0.202	1.7	0.195	0.024
Salience Ventral Attention A: Parietal Medial 1	8.8	0.006	0.109	5.3	0.007	0.130	10.2	0.002	0.126	3.3	0.015	0.162	3.8	0.056	0.052
Salience Ventral Attention A: Frontal Medial 1	14.3	0.001	0.165	7.1	0.002	0.166	6.5	0.013	0.084	4.1	0.005	0.193	2.5	0.118	0.035
Salience Ventral Attention B: Inferior Parietal Lobule 1	13.8	0.001	0.161	7.9	0.001	0.182	7.1	0.010	0.091	5.3	0.001	0.236	1.9	0.178	0.026
Salience Ventral Attention B: Lateral Prefrontal Cortex 1	12.9	0.001	0.152	8.2	0.001	0.187	4.7	0.033	0.062	5.7	0.000	0.250	0.6	0.446	0.008
Salience Ventral Attention B: Medial Posterior Prefrontal 1	15.6	0.001	0.178	10.2	0.000	0.223	19.8	0.000	0.218	5.3	0.001	0.236	8.9	0.004	0.114
LimbiC: B: Orbital Frontal Cortex 1	20.0	0.000	0.217	10.9	0.000	0.236	20.7	0.000	0.226	6.9	0.000	0.284	5.7	0.019	0.077
LimbiC: A: Temporal Pole 1	33.4	0.000	0.317	17.1	0.000	0.325	29.1	0.000	0.291	10.8	0.000	0.386	11.5	0.001	0.143
Control A: Intraparietal Sulcus 1	10.5	0.003	0.127	5.2	0.008	0.127	7.1	0.010	0.090	4.4	0.003	0.202	1.5	0.232	0.021
Control A: Lateral Prefrontal Cortex 1	16.2	0.000	0.183	8.3	0.001	0.189	8.7	0.004	0.109	5.7	0.001	0.248	2.9	0.094	0.040
Control A: Lateral Prefrontal Cortex 2	20.8	0.000	0.224	11.9	0.000	0.252	7.3	0.009	0.093	6.6	0.000	0.276	3.4	0.070	0.047
Control B: Temporal 1	23.4	0.000	0.246	12.1	0.000	0.254	15.7	0.000	0.181	7.9	0.000	0.314	4.5	0.037	0.061
Control B: inferior parietal lobule 1	9.1	0.005	0.112	4.5	0.015	0.112	6.5	0.013	0.084	3.3	0.015	0.162	2.1	0.157	0.029
Control B: Lateral Prefrontal Cortexd 1	15.6	0.001	0.178	8.1	0.001	0.185	6.6	0.012	0.085	4.6	0.002	0.212	2.2	0.139	0.031
Control B: Lateral Prefrontal Cortexv 1	18.4	0.000	0.203	9.1	0.000	0.203	15.7	0.000	0.182	8.4	0.000	0.326	2.0	0.159	0.029
Control C: Cingulate Posterior 1	9.9	0.004	0.121	4.9	0.010	0.122	6.4	0.013	0.083	3.1	0.022	0.150	1.9	0.169	0.027
Control C: Precuneus 1	5.9	0.021	0.076	3.0	0.057	0.078	4.2	0.045	0.055	2.1	0.096	0.107	1.3	0.261	0.018
Default A: Inferior Parietal Lobule 1	9.2	0.005	0.114	4.8	0.012	0.118	3.3	0.072	0.045	3.1	0.022	0.151	1.1	0.294	0.016
Default A: Dorsal Prefrontal Cortex 1	20.3	0.000	0.220	10.6	0.000	0.230	10.0	0.002	0.123	5.6	0.001	0.245	5.4	0.023	0.073
Default A: Precuneus Posterior Cingulate Cortex 1	3.8	0.056	0.051	2.1	0.136	0.055	2.2	0.142	0.030	1.6	0.194	0.083	0.4	0.543	0.005
Default A: Medial Prefrontal Cortex 1	17.8	0.000	0.199	9.4	0.000	0.209	10.3	0.002	0.127	5.8	0.000	0.250	2.5	0.115	0.036
Default B: Dorsal Prefrontal Cortex 1	24.2	0.000	0.251	12.6	0.000	0.262	8.8	0.004	0.110	7.0	0.000	0.288	4.2	0.045	0.057
Default B: Ventral Prefrontal Cortex 1	21.4	0.000	0.229	10.6	0.000	0.229	16.1	0.000	0.185	6.7	0.000	0.278	5.7	0.020	0.077
Default B: Ventral Prefrontal Cortex 2	19.9	0.000	0.216	10.7	0.000	0.232	8.6	0.004	0.108	6.5	0.000	0.273	2.6	0.111	0.036
Default C: Retro Superior Parietal Lobuleenial 1	5.7	0.022	0.073	2.9	0.061	0.076	3.9	0.052	0.052	2.5	0.052	0.126	0.5	0.469	0.008
Default C: Parahippocampal Cortex 1	15.7	0.001	0.179	8.1	0.001	0.185	11.8	0.001	0.143	4.1	0.005	0.191	5.5	0.022	0.074
Temporal Parietal 1	22.1	0.000	0.235	10.9	0.000	0.236	16.6	0.000	0.190	5.9	0.000	0.255	7.4	0.008	0.097
Temporal Parietal 2	12.5	0.001	0.148	6.3	0.003	0.150	8.1	0.006	0.102	4.2	0.004	0.195	1.8	0.183	0.026
Temporal Parietal 3	5.7	0.023	0.074	3.0	0.058	0.077	4.8	0.032	0.063	2.4	0.059	0.121	1.3	0.253	0.019

2.2 The Effect of Age and Insulin Resistance on Regional CBF

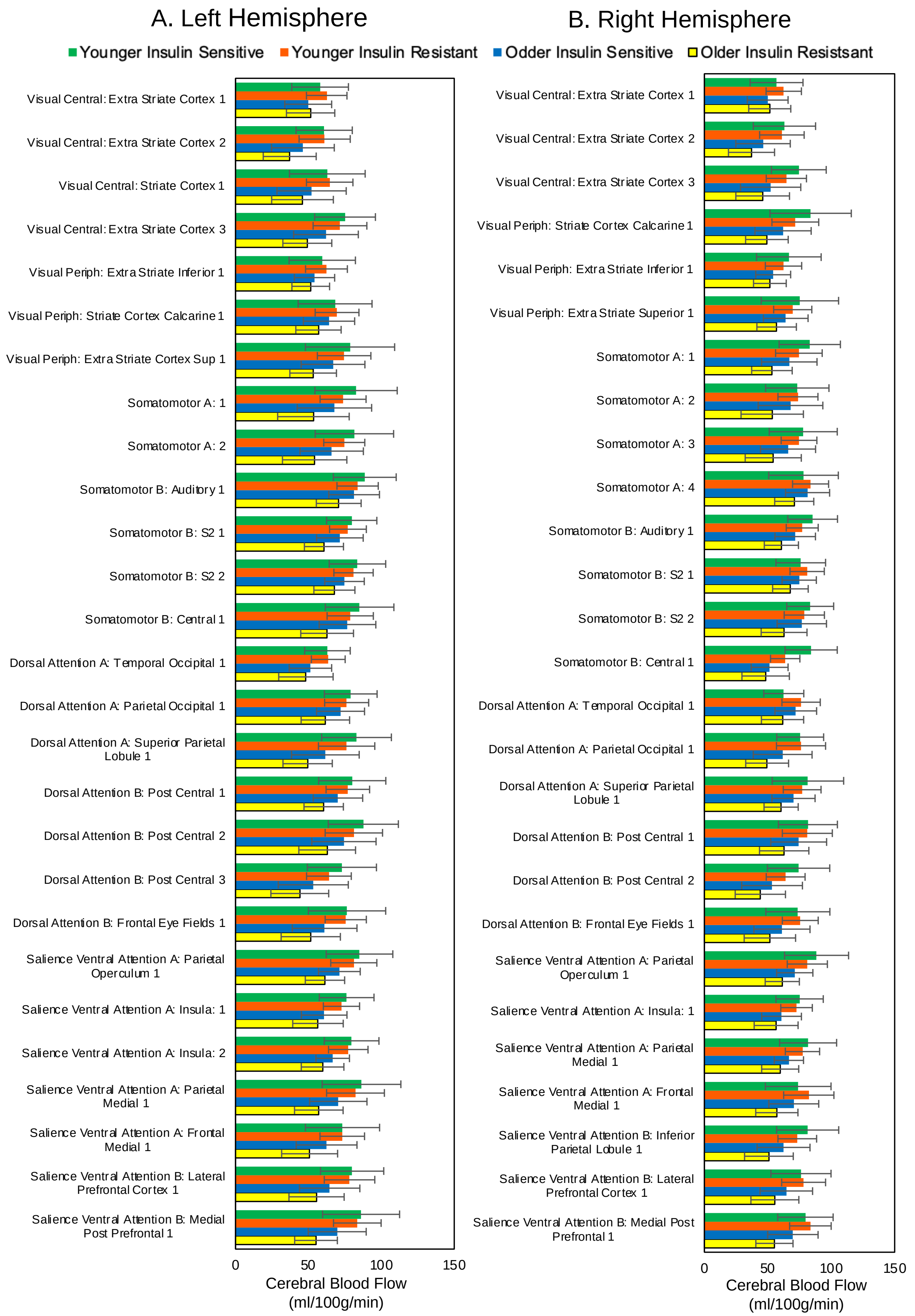

Continued …

Continued …

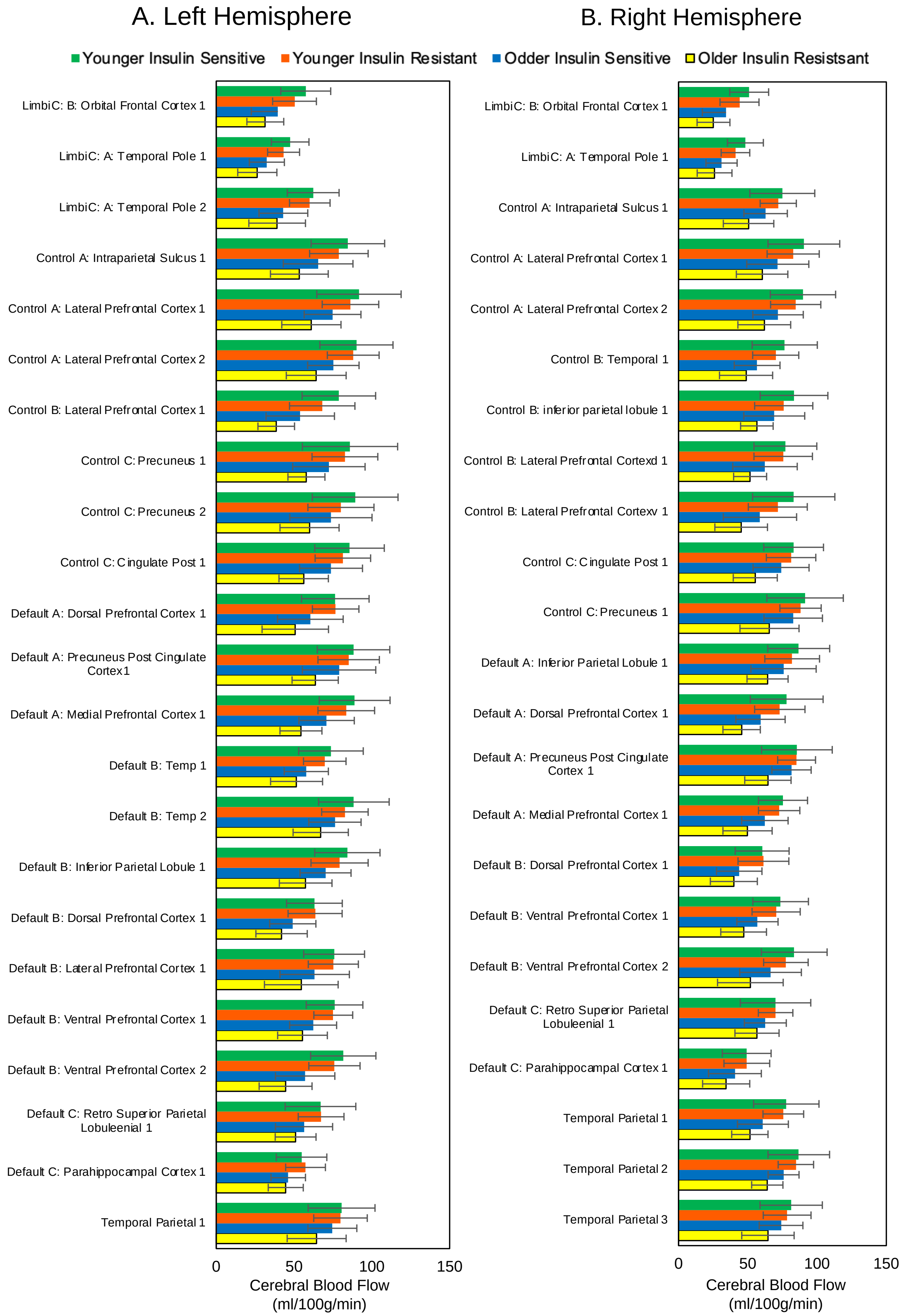

Figure S2. Mean and standard deviation (error bars) of regional cerebral blood flow for four groups based on age group and median HOMA-IR split. The data is also presented in Table S3.

Table S3. Mean and standard deviation (SD) of regional cerebral blood flow (ml/100g/min) among the four subgroups based on age group and HOMA-IR median split. The data is also displayed in Figure S2.

Left Hemisphere

	Younger Insulin Sensitive		Younger Insulin Resistant		Odler Insulin Sensitive		Older Insulin Resistsant	
	Mean	SD	Mean	SD	Mean	SD	Mean	SD
Visual Central: Extra Striate Cortex 1	58.0	19.4	62.5	13.9	49.8	16.1	51.5	16.6
Visual Central: Extra Striate Cortex 2	60.8	19.3	61.1	17.5	46.2	21.5	37.1	18.2
Visual Central: Striate Cortex 1	62.9	26.0	64.6	15.9	52.2	23.7	45.9	21.2
Visual Central: Extra Striate Cortex 3	75.1	20.8	71.6	18.4	62.0	22.2	49.2	16.8
Visual Peripheral: Extra Striate Inferior 1	59.4	22.8	62.4	14.3	54.2	13.8	51.6	12.9
Visual Peripheral: Striate Cortex Calcarine 1	68.3	25.4	69.6	15.0	64.1	17.6	56.9	15.5
Visual Peripheral: Extra Striate Cortex Sup 1	78.5	30.6	74.5	18.4	66.9	21.9	53.2	16.0
Somatomotor A: 1	82.7	28.3	73.7	15.8	67.8	25.5	53.4	24.6
Somatomotor A: 2	81.5	26.9	74.6	14.1	65.9	21.7	54.3	22.1
Somatomotor B: Auditory 1	88.6	21.6	83.7	14.2	81.3	17.3	70.8	15.4
Somatomotor B: S2 1	79.7	17.2	77.1	12.6	71.6	16.0	60.6	13.4
Somatomotor B: S2 2	83.6	19.4	81.0	13.5	74.7	13.5	67.8	14.1
Somatomotor B: Central 1	85.0	23.5	78.7	15.9	76.8	19.5	62.8	18.1
Dorsal Attention A: Temporal Occipital 1	63.0	15.6	63.6	11.6	51.4	14.6	48.3	18.6
Dorsal Attention A: Parietal Occipital 1	79.0	18.1	76.2	15.1	72.0	16.5	61.6	16.6
Dorsal Attention A: Superior Parietal Lobule 1	83.0	23.9	76.1	19.3	61.6	23.2	49.4	16.9
Dorsal Attention B: Post Central 1	80.1	23.0	77.0	14.9	70.2	17.0	60.4	13.5
Dorsal Attention B: Post Central 2	87.7	24.1	81.2	19.7	74.3	22.0	62.9	19.5
Dorsal Attention B: Post Central 3	73.0	23.7	64.0	15.3	53.3	24.0	44.1	19.8
Dorsal Attention B: Frontal Eye Fields 1	76.5	26.5	75.6	14.1	61.1	22.2	51.6	20.3
Salience Ventral Attention A: Parietal Operculum 1	85.0	22.9	81.1	15.8	71.2	14.3	61.4	13.5
Salience Ventral Attention A: Insula: 1	76.2	18.7	72.7	12.4	60.8	15.7	56.5	17.3
Salience Ventral Attention A: Insula: 2	79.6	18.8	77.3	13.5	66.6	11.5	59.7	14.6
Salience Ventral Attention A: Parietal Medial 1	86.4	27.0	82.2	19.8	70.4	19.7	57.1	16.6
Salience Ventral Attention A: Frontal Medial 1	73.3	25.4	73.2	15.3	62.4	20.8	50.8	19.2
Salience Ventral Attention B: Lateral Prefrontal Cortex 1	79.9	21.7	78.2	17.3	64.5	20.7	55.5	18.9
Salience Ventral Attention B: Medial Posterior Prefrontal 1	86.1	26.4	83.5	16.4	69.8	19.9	55.2	14.6
Limbic B: Orbital Frontal Cortex 1	57.5	16.1	50.3	14.1	39.7	17.1	31.5	11.8
Limbic A: Temporal Pole 1	47.5	12.1	43.3	10.3	32.5	11.3	26.4	12.5
Limbic A: Temporal Pole 2	62.3	16.6	60.1	13.0	43.2	15.7	39.2	18.2
Control A: Intraparietal Sulcus 1	84.6	23.6	78.8	18.8	65.5	22.4	53.4	18.6
Control A: Lateral Prefrontal Cortex 1	91.7	27.1	86.2	18.1	74.8	18.2	61.1	19.1
Control A: Lateral Prefrontal Cortex 2	90.1	23.5	88.1	16.6	75.3	16.5	64.3	19.2
Control B: Lateral Prefrontal Cortex 1	78.8	23.6	68.1	21.0	54.0	22.0	38.6	11.7
Control C: Precuneus 1	86.0	30.6	82.7	21.1	72.4	23.3	58.0	11.9
Control C: Precuneus 2	89.3	27.5	80.2	21.2	73.7	26.4	60.0	19.0
Control C: Cingulate Posterior 1	85.6	22.4	81.4	17.8	73.8	20.2	56.2	15.9
Default A: Dorsal Prefrontal Cortex 1	76.5	21.7	76.7	14.9	60.5	21.1	50.8	21.3
Default A: Precuneus Posterior Cingulate Cortex1	88.2	23.3	85.1	19.7	78.9	23.6	63.6	14.8
Default A: Medial Prefrontal Cortex 1	89.0	22.7	83.6	18.2	70.9	17.8	54.4	13.5
Default B: Temp 1	73.7	20.7	69.8	13.7	57.9	14.2	51.6	16.7
Default B: Temp 2	88.4	22.7	82.7	14.9	76.3	16.8	67.2	17.7
Default B: Inferior Parietal Lobule 1	84.3	21.0	79.3	18.3	70.3	16.4	57.5	17.0
Default B: Dorsal Prefrontal Cortex 1	63.1	17.9	63.6	17.4	49.2	14.8	42.0	16.5
Default B: Lateral Prefrontal Cortex 1	75.8	19.5	75.3	16.1	63.3	22.3	54.7	23.7
Default B: Ventral Prefrontal Cortex 1	76.0	18.2	75.2	12.5	62.2	15.1	55.4	16.0
Default B: Ventral Prefrontal Cortex 2	81.6	21.0	76.0	16.5	57.1	19.1	44.6	17.0
Default C: Retro Superior Parietal Lobuleenial 1	67.0	22.7	67.4	14.7	56.5	18.4	51.0	13.1
Default C: Parahippocampal Cortex 1	54.8	16.3	57.4	12.8	46.3	11.1	44.7	11.3
Temporal Parietal 1	80.6	21.5	79.9	17.2	74.6	15.7	64.5	18.9

Right Hemisphere

	Younger Insulin Sensitive		Younger Insulin Resistant		Odler Insulin Sensitive		Older Insulin Resistsant	
	Mean	SD	Mean	SD	Mean	SD	Mean	SD
Visual Central: Extra Striate Cortex 1	56.9	20.8	57.7	13.0	53.4	13.2	48.4	10.2
Visual Central: Extra Striate Cortex 2	63.0	24.6	63.6	17.3	50.3	21.4	36.6	17.3
Visual Central: Extra Striate Cortex 3	74.4	21.7	71.8	15.7	66.4	21.5	50.6	17.3
Visual Peripheral: Striate Cortex Calcarine 1	83.7	32.1	83.9	17.9	78.5	21.9	70.2	15.6
Visual Peripheral: Extra Striate Inferior 1	66.5	25.5	66.7	13.2	64.5	16.6	57.3	11.8
Visual Peripheral: Extra Striate Superior 1	75.2	30.5	74.8	17.3	70.0	25.2	54.3	14.3
Somatomotor A: 1	83.0	24.2	82.7	17.4	78.4	21.9	63.2	20.0
Somatomotor A: 2	73.2	25.0	71.4	17.4	63.5	24.1	51.9	20.2
Somatomotor A: 3	77.9	26.9	72.0	14.7	60.3	26.6	50.4	23.5
Somatomotor A: 4	78.1	27.4	73.9	16.1	64.4	23.7	55.1	21.0
Somatomotor B: Auditory 1	85.3	19.6	82.5	12.7	77.8	15.3	65.7	17.1
Somatomotor B: S2 1	76.0	19.6	74.7	10.0	68.2	13.7	60.2	13.8
Somatomotor B: S2 2	83.4	18.5	80.8	12.3	72.9	14.4	65.2	15.2
Somatomotor B: Central 1	84.2	20.6	78.7	12.7	77.1	15.5	66.1	19.2
Dorsal Attention A: Temporal Occipital 1	62.5	15.8	59.4	10.5	50.7	15.4	45.6	15.6
Dorsal Attention A: Parietal Occipital 1	75.5	18.5	74.1	14.6	68.0	15.9	57.7	16.2
Dorsal Attention A: Superior Parietal Lobule 1	81.6	28.2	74.1	17.4	66.9	23.8	49.8	15.4
Dorsal Attention B: Post Central 1	81.6	23.2	78.5	14.8	73.5	20.3	61.2	15.5
Dorsal Attention B: Post Central 2	74.2	24.6	69.4	15.7	60.3	23.0	49.6	18.2
Dorsal Attention B: Frontal Eye Fields 1	73.7	25.3	71.1	14.1	57.7	19.9	47.9	16.9
Salience Ventral Attention A: Parietal Operculum 1	88.4	25.2	81.4	12.6	74.4	17.6	63.9	11.6
Salience Ventral Attention A: Insula: 1	75.0	18.6	74.4	11.1	65.6	12.5	57.3	13.4
Salience Ventral Attention A: Parietal Medial 1	81.7	22.4	81.8	18.6	71.9	23.2	58.4	20.8
Salience Ventral Attention A: Frontal Medial 1	73.9	25.9	72.4	15.5	59.1	21.2	47.9	18.9
Salience Ventral Attention B: Inferior Parietal Lobule 1	81.4	24.5	73.6	13.3	64.7	18.2	56.8	10.9
Salience Ventral Attention B: Lateral Prefrontal Cortex 1	76.1	23.7	73.1	13.1	61.9	21.2	51.6	18.7
Salience Ventral Attention B: Medial Posterior Prefrontal 1	79.6	21.8	78.5	17.5	66.1	17.4	54.4	18.0
Limbic B: Orbital Frontal Cortex 1	51.1	14.0	44.0	13.8	34.3	17.0	25.2	13.7
Limbic A: Temporal Pole 1	48.3	13.0	41.1	8.1	31.1	10.8	26.1	11.7
Control A: Intraparietal Sulcus 1	74.9	23.4	72.0	16.5	62.9	21.2	50.6	16.0
Control A: Lateral Prefrontal Cortex 1	90.5	25.9	82.8	15.6	71.6	20.0	60.4	16.1
Control A: Lateral Prefrontal Cortex 2	89.9	23.5	84.6	14.3	71.8	15.9	61.9	16.4
Control B: Temporal 1	76.7	23.5	70.2	11.7	56.8	15.8	48.8	16.7
Control B: inferior parietal lobule 1	83.4	24.4	76.0	19.2	69.1	21.1	56.6	16.3
Control B: Lateral Prefrontal Cortexd 1	77.1	22.7	75.6	16.7	62.4	19.5	51.7	18.2
Control B: Lateral Prefrontal Cortexv 1	83.1	29.7	71.7	18.8	58.8	23.0	45.2	15.4
Control C: Cingulate Posterior 1	83.1	21.5	81.2	17.7	74.0	20.8	55.4	17.2
Control C: Precuneus 1	91.3	27.6	88.1	19.8	82.8	24.5	65.7	16.2
Default A: Inferior Parietal Lobule 1	86.8	22.3	82.0	15.8	75.8	17.4	64.3	13.1
Default A: Dorsal Prefrontal Cortex 1	78.1	26.3	73.1	15.1	59.2	20.1	45.6	17.2
Default A: Precuneus Posterior Cingulate Cortex 1	85.5	25.5	85.2	16.7	81.5	25.1	64.5	17.0
Default A: Medial Prefrontal Cortex 1	75.5	17.7	72.6	17.4	62.4	15.5	49.9	12.6
Default B: Dorsal Prefrontal Cortex 1	60.4	19.5	61.4	16.2	43.8	14.7	39.9	16.3
Default B: Ventral Prefrontal Cortex 1	73.7	20.1	70.5	15.5	57.0	16.3	47.0	17.3
Default B: Ventral Prefrontal Cortex 2	83.5	23.8	77.5	14.4	66.4	16.2	51.9	17.3
Default C: Retro Superior Parietal Lobuleenial 1	70.0	25.4	70.1	14.1	62.7	17.7	56.7	10.9
Default C: Parahippocampal Cortex 1	49.2	17.6	49.3	10.5	40.8	9.4	34.4	10.2
Temporal Parietal 1	77.8	23.6	75.7	13.6	60.9	15.4	51.5	18.3
Temporal Parietal 2	86.8	22.2	84.7	13.8	75.9	15.9	64.1	16.4
Temporal Parietal 3	81.3	22.5	78.4	13.3	74.0	15.8	64.6	13.1

Table S4. General linear models of the association between regional cerebral blood flow among the four groups based on age category and insulin resistance levels (HOMA-IR median split), with blood pressure and cortical thickness as covariates. Post-hoc contrasts in the 40 regions with significant group difference are shown, comparing the young insulin sensitive group to the other three groups. The group effect sizes are plotted on the brain surface in Figure 2 in main document.

	Overall			4 Groups Based on Age Group and HOMA-IR Median Split						Systolic BP			Diastolic BP			Cortical Thickness		
	F	p-FDR	η^2_p	F	p	η^2_p	YIS vs YIR	YIS vs OIS	YIS vs OIR	F	p	η^2_p	F	p	η^2_p	F	p	η^2_p
Visual Central: Extra Striate Cortex 1	2.2	0.060	0.165	1.4	0.264	0.057				0.4	0.543	0.006	1.4	0.237	0.021	0.0	0.916	0.000
Visual Central: Extra Striate Cortex 2	6.1	0.000	0.352	3.2	0.028	0.126	0.361	0.781	0.040	2.4	0.126	0.035	2.6	0.113	0.037	0.2	0.658	0.003
Visual Central: Striate Cortex 1	3.3	0.011	0.230	1.4	0.259	0.058				2.1	0.151	0.031	0.8	0.385	0.011	2.0	0.161	0.029
Visual Central: Extra Striate Cortex 3	3.9	0.005	0.256	2.6	0.061	0.104				2.4	0.126	0.035	0.2	0.660	0.003	0.0	0.828	0.001
Visual Peripheral: Extra Striate Inferior 1	1.1	0.363	0.092	0.9	0.459	0.038				0.5	0.477	0.008	0.0	0.824	0.001	0.2	0.646	0.003
Visual Peripheral: Striate Cortex Calcarine 1	1.3	0.267	0.106	0.9	0.429	0.040				1.2	0.284	0.017	0.1	0.747	0.002	0.1	0.725	0.002
Visual Peripheral: Extra Striate Cortex Sup 1	2.5	0.038	0.183	2.0	0.128	0.081				1.5	0.224	0.022	0.0	0.951	0.000	0.4	0.536	0.006
Somatomotor A: 1	2.5	0.038	0.184	2.7	0.055	0.106				0.1	0.789	0.001	0.8	0.384	0.011	0.7	0.397	0.011
Somatomotor A: 2	2.4	0.044	0.177	2.4	0.073	0.098				0.0	0.879	0.000	0.2	0.658	0.003	0.2	0.664	0.003
Somatomotor B: Auditory 1	1.8	0.123	0.137	1.6	0.197	0.067				1.3	0.264	0.019	0.0	0.911	0.000	0.0	0.915	0.000
Somatomotor B: S2 1	3.4	0.010	0.235	2.6	0.061	0.104				2.2	0.143	0.032	0.1	0.769	0.001	0.0	0.927	0.000
Somatomotor B: S2 2	2.2	0.058	0.166	1.7	0.178	0.070				1.2	0.282	0.017	0.0	0.939	0.000	0.5	0.489	0.007
Somatomotor B: Central 1	2.5	0.040	0.181	2.5	0.065	0.102				1.1	0.297	0.016	0.3	0.608	0.004	0.1	0.715	0.002
Dorsal Attention A: Temporal Occipital 1	3.8	0.006	0.255	2.1	0.104	0.087				0.6	0.456	0.008	2.0	0.166	0.028	0.1	0.787	0.001
Dorsal Attention A: Parietal Occipital 1	3.2	0.013	0.222	2.3	0.089	0.092				2.6	0.109	0.038	0.4	0.538	0.006	1.1	0.292	0.017
Dorsal Attention A: Superior Parietal Lobule 1	5.5	0.001	0.330	4.6	0.005	0.171	0.629	0.067	0.001	1.9	0.169	0.028	0.2	0.656	0.003	1.9	0.173	0.028
Dorsal Attention B: Post Central 1	2.1	0.068	0.159	1.9	0.137	0.079				0.8	0.383	0.011	0.0	0.827	0.001	0.0	0.925	0.000
Dorsal Attention B: Post Central 2	2.7	0.030	0.193	3.0	0.035	0.120	0.614	0.169	0.006	0.4	0.547	0.005	0.6	0.455	0.008	2.2	0.143	0.032
Dorsal Attention B: Post Central 3	3.7	0.006	0.251	3.6	0.017	0.140	0.388	0.050	0.002	1.5	0.227	0.022	0.0	0.850	0.001	3.7	0.059	0.052
Dorsal Attention B: Frontal Eye Fields 1	3.3	0.012	0.227	1.5	0.227	0.062				0.1	0.737	0.002	0.5	0.471	0.008	1.6	0.215	0.023
Salience Ventral Attention A: Parietal Operculum 1	4.3	0.003	0.276	3.4	0.024	0.131	0.893	0.306	0.007	0.5	0.494	0.007	1.3	0.263	0.019	0.1	0.763	0.001
Salience Ventral Attention A: Insula: 1	3.5	0.009	0.236	1.9	0.135	0.079				0.2	0.646	0.003	0.1	0.704	0.002	1.8	0.183	0.026
Salience Ventral Attention A: Insula: 2	3.9	0.005	0.259	2.9	0.041	0.115	0.790	0.089	0.008	2.0	0.158	0.029	0.2	0.658	0.003	0.6	0.444	0.009
Salience Ventral Attention A: Parietal Medial 1	4.3	0.003	0.276	4.0	0.011	0.152	0.969	0.227	0.004	0.5	0.470	0.008	1.1	0.297	0.016	0.5	0.469	0.008
Salience Ventral Attention A: Frontal Medial 1	2.6	0.035	0.187	1.3	0.295	0.053				0.0	0.936	0.000	0.5	0.461	0.008	0.8	0.372	0.012
Salience Ventral Attention B: Lateral Prefrontal Cortex 1	3.6	0.007	0.245	2.0	0.118	0.083				2.9	0.091	0.042	0.1	0.796	0.001	0.1	0.713	0.002
Salience Ventral Attention B: Medial Posterior Prefrontal 1	4.8	0.002	0.299	5.8	0.001	0.207	0.965	0.036	0.000	0.0	0.943	0.000	0.6	0.449	0.009	2.0	0.165	0.029
Limbic: B: Orbital Frontal Cortex 1	6.6	0.000	0.372	4.5	0.006	0.167	0.392	0.029	0.001	3.0	0.088	0.043	0.0	0.936	0.000	4.4	0.040	0.062
Limbic: A: Temporal Pole 1	8.6	0.000	0.435	4.6	0.006	0.169	0.434	0.031	0.001	2.2	0.140	0.032	0.6	0.444	0.009	5.1	0.028	0.070
Limbic: A: Temporal Pole 2	6.2	0.000	0.356	5.2	0.003	0.189	0.776	0.029	0.002	0.2	0.673	0.003	2.6	0.115	0.037	0.2	0.641	0.003
Control A: Intraparietal Sulcus 1	4.4	0.003	0.285	3.7	0.017	0.141	0.835	0.218	0.004	0.6	0.453	0.008	1.1	0.297	0.016	0.0	0.993	0.000
Control A: Lateral Prefrontal Cortex 1	4.4	0.003	0.282	3.5	0.021	0.135	0.633	0.160	0.004	3.0	0.088	0.043	0.1	0.759	0.001	1.2	0.286	0.017
Control A: Lateral Prefrontal Cortex 2	4.5	0.003	0.287	3.0	0.036	0.119	0.983	0.254	0.012	3.2	0.080	0.045	0.0	0.972	0.000	0.7	0.406	0.010
Control B: Lateral Prefrontal Cortex 1	8.4	0.000	0.428	5.1	0.003	0.186	0.256	0.085	0.000	8.3	0.005	0.111	0.4	0.551	0.005	1.5	0.228	0.022
Control C: Precuneus 1	3.2	0.012	0.225	3.1	0.031	0.123	0.929	0.316	0.010	0.8	0.373	0.012	0.4	0.533	0.006	1.1	0.308	0.016
Control C: Precuneus 2	2.5	0.041	0.180	3.0	0.035	0.119	0.390	0.156	0.005	0.5	0.480	0.007	0.0	0.869	0.000	2.0	0.167	0.028
Control C: Cingulate Posterior 1	4.9	0.002	0.303	5.1	0.003	0.185	0.912	0.369	0.002	1.3	0.257	0.019	0.6	0.460	0.008	1.3	0.264	0.019
Default A: Dorsal Prefrontal Cortex 1	3.9	0.005	0.261	2.7	0.050	0.109	0.735	0.265	0.022	1.7	0.202	0.024	0.0	0.996	0.000	0.0	0.890	0.000
Default A: Precuneus Posterior Cingulate Cortex1	2.7	0.029	0.194	2.5	0.064	0.102				1.1	0.294	0.016	0.2	0.650	0.003	0.1	0.820	0.001
Default A: Medial Prefrontal Cortex 1	5.9	0.001	0.346	5.9	0.001	0.209	0.635	0.081	0.000	1.1	0.289	0.017	0.0	0.939	0.000	0.3	0.618	0.004
Default B: Temp 1	4.5	0.003	0.286	1.5	0.230	0.062				1.4	0.246	0.020	0.1	0.731	0.002	2.0	0.164	0.029
Default B: Temp 2	2.8	0.024	0.201	2.5	0.063	0.102				1.1	0.291	0.017	0.1	0.802	0.001	1.5	0.232	0.021
Default B: Inferior Parietal Lobule 1	4.9	0.002	0.306	3.2	0.029	0.125	0.895	0.493	0.013	2.2	0.143	0.032	0.7	0.408	0.010	0.1	0.811	0.001
Default B: Dorsal Prefrontal Cortex 1	3.9	0.005	0.259	1.4	0.238	0.061				1.6	0.205	0.024	0.1	0.706	0.002	0.2	0.622	0.004
Default B: Lateral Prefrontal Cortex 1	2.8	0.024	0.201	1.9	0.138	0.078				2.1	0.151	0.031	0.0	0.925	0.000	0.8	0.376	0.012
Default B: Ventral Prefrontal Cortex 1	4.0	0.005	0.264	4.2	0.008	0.159	0.807	0.050	0.004	0.3	0.559	0.005	0.0	0.831	0.001	1.2	0.279	0.017
Default B: Ventral Prefrontal Cortex 2	7.9	0.000	0.415	5.1	0.003	0.186	0.622	0.030	0.000	3.2	0.079	0.045	0.0	0.833	0.001	0.1	0.798	0.001
Default C: Retro Superior Parietal Lobuleenial 1	3.4	0.010	0.232	1.3	0.284	0.055				1.7	0.203	0.024	0.8	0.364	0.012	1.3	0.262	0.019
Default C: Parahippocampal Cortex 1	2.5	0.037	0.184	2.0	0.124	0.082				0.1	0.791	0.001	0.4	0.550	0.005	1.0	0.314	0.015
Temporal Parietal 1	1.9	0.103	0.144	1.9	0.144	0.077				1.2	0.282	0.017	0.1	0.791	0.001	0.9	0.354	0.013

	Overall			4 Groups Based on Age Group and HOMA-IR Median Split						Systolic BP			Diastolic BP			Cortical Thickness		
	F	p-FDR	η^2_p	F	p	η^2_p	YIS vs YIR	YIS vs OIS	YIS vs OIR	F	p	η^2_p	F	p	η^2_p	F	p	η^2_p
Visual Central: Extra Striate Cortex 1	0.8	0.544	0.070	0.7	0.558	0.030				0.3	0.566	0.005	0.0	0.845	0.001	0.0	0.959	0.000
Visual Central: Extra Striate Cortex 2	4.5	0.003	0.289	4.2	0.009	0.158	0.458	0.663	0.013	0.7	0.422	0.010	1.7	0.198	0.025	0.2	0.679	0.003
Visual Central: Extra Striate Cortex 3	3.9	0.005	0.260	3.3	0.024	0.130	0.873	0.974	0.027	3.1	0.081	0.045	0.2	0.642	0.003	0.7	0.391	0.011
Visual Peripheral: Striate Cortex Calcarine 1	1.0	0.412	0.086	0.7	0.567	0.030				0.8	0.380	0.012	0.1	0.769	0.001	0.1	0.768	0.001
Visual Peripheral: Extra Striate Inferior 1	0.9	0.476	0.078	0.8	0.518	0.033				0.9	0.350	0.013	0.1	0.752	0.002	0.1	0.819	0.001
Visual Peripheral: Extra Striate Superior 1	2.1	0.067	0.160	2.4	0.074	0.098				0.6	0.447	0.009	0.6	0.425	0.010	0.3	0.569	0.005
Somatomotor A: 1	2.9	0.021	0.206	2.6	0.057	0.106				0.3	0.598	0.004	0.8	0.386	0.011	4.5	0.037	0.064
Somatomotor A: 2	2.2	0.057	0.167	1.9	0.134	0.079				0.1	0.781	0.001	0.8	0.377	0.012	1.8	0.188	0.026
Somatomotor A: 3	2.5	0.036	0.186	1.6	0.203	0.066				0.9	0.358	0.013	0.1	0.767	0.001	0.2	0.690	0.002
Somatomotor A: 4	2.2	0.062	0.164	1.3	0.280	0.055				0.1	0.822	0.001	0.7	0.416	0.010	1.4	0.248	0.020
Somatomotor B: Auditory 1	3.1	0.015	0.218	2.2	0.094	0.090				2.5	0.118	0.036	0.0	0.935	0.000	0.7	0.415	0.010
Somatomotor B: S2 1	2.6	0.032	0.190	1.6	0.196	0.067				2.0	0.159	0.029	0.0	0.888	0.000	0.0	0.968	0.000
Somatomotor B: S2 2	3.8	0.006	0.253	1.3	0.290	0.054				3.3	0.074	0.047	0.3	0.608	0.004	3.3	0.074	0.047
Somatomotor B: Central 1	2.3	0.056	0.168	1.9	0.136	0.079				1.5	0.224	0.022	0.0	0.864	0.000	0.5	0.486	0.007
Dorsal Attention A: Temporal Occipital 1	3.6	0.007	0.246	2.3	0.085	0.094				0.9	0.344	0.013	1.4	0.247	0.020	0.1	0.753	0.001
Dorsal Attention A: Parietal Occipital 1	2.9	0.020	0.207	2.4	0.072	0.098				1.0	0.321	0.015	0.8	0.372	0.012	0.0	0.895	0.000
Dorsal Attention A: Superior Parietal Lobule 1	4.2	0.004	0.274	4.6	0.006	0.170	0.630	0.316	0.002	0.6	0.449	0.009	1.0	0.314	0.015	0.8	0.360	0.013
Dorsal Attention B: Post Central 1	2.9	0.020	0.208	2.5	0.069	0.100				2.3	0.136	0.033	0.2	0.668	0.003	1.3	0.256	0.019
Dorsal Attention B: Post Central 2	2.9	0.022	0.205	2.8	0.046	0.112	0.829	0.236	0.011	0.3	0.564	0.005	0.6	0.446	0.009	1.1	0.308	0.016
Dorsal Attention B: Frontal Eye Fields 1	3.4	0.010	0.232	2.7	0.052	0.108				0.1	0.806	0.001	0.4	0.550	0.005	0.3	0.601	0.004
Salience Ventral Attention A: Parietal Operculum 1	3.8	0.005	0.256	2.2	0.101	0.088				2.1	0.156	0.030	0.0	0.838	0.001	1.1	0.300	0.016
Salience Ventral Attention A: Insula: 1	3.8	0.006	0.252	2.1	0.109	0.086				1.7	0.197	0.025	0.0	0.832	0.001	0.5	0.479	0.008
Salience Ventral Attention A: Parietal Medial 1	3.3	0.011	0.229	3.3	0.026	0.128	0.612	0.545	0.021	0.8	0.369	0.012	0.7	0.408	0.010	1.4	0.249	0.020
Salience Ventral Attention A: Frontal Medial 1	3.5	0.009	0.238	2.2	0.097	0.089				0.6	0.460	0.008	0.6	0.439	0.009	0.0	0.942	0.000
Salience Ventral Attention B: Inferior Parietal Lobule 1	4.2	0.004	0.273	1.8	0.159	0.074				1.4	0.236	0.021	0.4	0.510	0.007	1.2	0.274	0.018
Salience Ventral Attention B: Lateral Prefrontal Cortex 1	4.3	0.003	0.279	1.1	0.354	0.047				2.8	0.097	0.041	0.1	0.787	0.001	2.0	0.162	0.029
Salience Ventral Attention B: Medial Posterior Prefrontal 1	4.5	0.003	0.288	4.7	0.005	0.174	0.903	0.088	0.002	0.6	0.433	0.009	0.1	0.716	0.002	3.1	0.081	0.045
Limbic: B: Orbital Frontal Cortex 1	5.5	0.001	0.329	3.5	0.021	0.134	0.306	0.074	0.002	3.6	0.063	0.051	0.2	0.636	0.003	0.8	0.378	0.012
Limbic: A: Temporal Pole 1	8.6	0.000	0.435	6.0	0.001	0.212	0.134	0.005	0.000	1.8	0.188	0.026	0.5	0.474	0.008	1.3	0.260	0.019
Control A: Intraparietal Sulcus 1	4.1	0.004	0.270	2.6	0.059	0.105				1.8	0.185	0.026	1.2	0.275	0.018	0.0	0.903	0.000
Control A: Lateral Prefrontal Cortex 1	4.8	0.002	0.298	2.6	0.058	0.105				3.2	0.076	0.046	0.0	0.991	0.000	0.0	0.939	0.000
Control A: Lateral Prefrontal Cortex 2	5.3	0.001	0.320	2.6	0.059	0.104				0.5	0.463	0.008	0.5	0.501	0.007	2.1	0.149	0.031
Control B: Temporal 1	6.1	0.001	0.353	2.9	0.043	0.114	0.404	0.106	0.005	3.1	0.083	0.044	0.0	0.901	0.000	1.0	0.325	0.014
Control B: inferior parietal lobule 1	3.3	0.011	0.230	2.7	0.053	0.108				0.8	0.378	0.012	1.0	0.320	0.015	0.0	0.827	0.001
Control B: Lateral Prefrontal Cortexd 1	3.7	0.006	0.251	1.9	0.130	0.080				0.9	0.334	0.014	0.2	0.641	0.003	0.6	0.446	0.009
Control B: Lateral Prefrontal Cortexv 1	7.0	0.000	0.384	2.8	0.045	0.112	0.316	0.281	0.008	9.0	0.004	0.119	0.3	0.613	0.004	0.0	0.884	0.000
Control C: Cingulate Posterior 1	4.1	0.004	0.270	4.4	0.007	0.165	0.796	0.991	0.013	0.7	0.414	0.010	1.0	0.333	0.014	0.3	0.595	0.004
Control C: Precuneus 1	2.7	0.028	0.195	2.9	0.040	0.116	0.909	0.916	0.031	0.2	0.634	0.003	1.4	0.247	0.020	0.0	0.911	0.000
Default A: Inferior Parietal Lobule 1	3.1	0.016	0.215	2.2	0.093	0.091				1.2	0.286	0.017	0.3	0.608	0.004	0.0	0.901	0.000
Default A: Dorsal Prefrontal Cortex 1	4.7	0.002	0.297	3.5	0.019	0.137	0.696	0.122	0.003	0.3	0.570	0.005	0.3	0.568	0.005	0.3	0.609	0.004
Default A: Precuneus Posterior Cingulate Cortex 1	2.4	0.046	0.175	2.6	0.058	0.105				0.5	0.481	0.007	0.9	0.346	0.013	0.1	0.799	0.001
Default A: Medial Prefrontal Cortex 1	5.0	0.002	0.309	2.8	0.048	0.111	0.773	0.414	0.013	3.2	0.077	0.046	0.4	0.555	0.005	0.6	0.433	0.009
Default B: Dorsal Prefrontal Cortex 1	4.7	0.002	0.295	1.6	0.201	0.066				1.7	0.198	0.025	0.0	0.904	0.000	0.4	0.512	0.006
Default B: Ventral Prefrontal Cortex 1	5.3	0.001	0.322	3.4	0.023	0.132	0.919	0.200	0.005	2.9	0.096	0.041	0.0	0.954	0.000	0.0	0.973	0.000
Default B: Ventral Prefrontal Cortex 2	5.9	0.001	0.345	3.4	0.022	0.132	0.626	0.361	0.007	2.5	0.121	0.036	0.0	0.929	0.000	0.7	0.407	0.010
Default C: Retro Superior Parietal Lobuleenial 1	2.0	0.077	0.155	0.9	0.424	0.041				1.0	0.327	0.014	0.7	0.396	0.011	0.4	0.533	0.006
Default C: Parahippocampal Cortex 1	3.2	0.013	0.221	2.7	0.052	0.108				0.3	0.564	0.005	0.1	0.803	0.001	0.7	0.412	0.010
Temporal Parietal 1	4.6	0.002	0.293	3.7	0.015	0.143	0.981	0.120	0.004	0.7	0.417	0.010	0.3	0.617	0.004	0.0	0.974	0.000
Temporal Parietal 2	3.9	0.005	0.257	2.5	0.067	0.101				2.7	0.106	0.039	0.0	0.980	0.000	0.0	0.962	0.000
Temporal Parietal 3	2.4	0.041	0.179	2.0	0.117	0.084				1.3	0.249	0.020	0.4	0.550	0.005	0.4	0.508	0.007

YIS = younger insulin sensitive; YIR = younger insulin resistant; OIS = older insulin sensitive; OIR = older insulin resistant

Table S5. General linear models of the association between regional cerebral blood flow among the four sub-groups based on age category and HOMA-IR2 levels, with blood pressure and cortical thickness as covariates. Post-hoc contrasts in the 42 regions with significant group difference are shown, comparing the young insulin sensitive group to the other three groups. The location of the 40 regions is plotted on the brain surface in Figure 2 in main document.

Left Hemisphere

	Overall			4 Groups: Age Cat and HOMAIR2 Median Split			Post-Hoc Contrasts			Systolic BP			Diastolic BP			Cortical Thickness		
	F	p-FDR	η^2_p	F	p	η^2_p	YIS vs YIR	YIS vs OIS	YIS vs OIR	F	p	η^2_p	F	p	η^2_p	F	p	η^2_p
Visual Central: Extra Striate Cortex 1	1.9	0.101	0.145	0.8	0.500	0.000				0.3	0.567	0.005	1.1	0.307	0.016	0.0	0.945	0.000
Visual Central: Extra Striate Cortex 2	5.8	0.001	0.343	2.9	0.043	0.000	0.649	0.553	0.027	2.3	0.135	0.033	2.2	0.141	0.032	0.3	0.585	0.004
Visual Central: Striate Cortex 1	3.0	0.017	0.213	0.9	0.458	0.000				2.2	0.146	0.031	0.3	0.576	0.005	1.8	0.188	0.026
Visual Central: Extra Striate Cortex 3	4.0	0.005	0.265	2.9	0.043	0.000	0.819	0.605	0.017	2.5	0.122	0.035	0.2	0.692	0.002	0.0	0.934	0.000
Visual Peripheral: Extra Striate Inferior 1	1.0	0.411	0.085	0.7	0.556	0.000				0.5	0.466	0.008	0.0	0.966	0.000	0.2	0.634	0.003
Visual Peripheral: Striate Cortex Calcarine 1	1.3	0.279	0.104	0.9	0.454	0.000				1.2	0.278	0.018	0.0	0.952	0.000	0.0	0.905	0.000
Visual Peripheral: Extra Striate Cortex Sup 1	2.8	0.025	0.200	2.5	0.069	0.000				1.6	0.204	0.024	0.0	0.849	0.001	0.4	0.520	0.006
Somatomotor A: 1	2.2	0.060	0.165	2.1	0.107	0.000				0.1	0.701	0.002	0.4	0.531	0.006	0.4	0.524	0.006
Somatomotor A: 2	2.2	0.057	0.167	2.1	0.103	0.000				0.0	0.829	0.001	0.1	0.779	0.001	0.1	0.726	0.002
Somatomotor B: Auditory 1	1.8	0.111	0.141	1.7	0.173	0.000				1.3	0.257	0.019	0.0	0.961	0.000	0.0	0.942	0.000
Somatomotor B: S2 1	3.7	0.007	0.246	3.0	0.038	0.000	0.759	0.898	0.030	2.0	0.157	0.030	0.2	0.670	0.003	0.1	0.777	0.001
Somatomotor B: S2 2	2.5	0.038	0.184	2.2	0.093	0.000				1.1	0.301	0.016	0.1	0.805	0.001	0.7	0.403	0.010
Somatomotor B: Central 1	2.1	0.069	0.160	1.9	0.138	0.000				1.2	0.284	0.017	0.1	0.707	0.002	0.1	0.765	0.001
Dorsal Attention A: Temporal Occipital 1	3.9	0.005	0.257	2.2	0.096	0.000				0.5	0.480	0.007	2.0	0.166	0.028	0.1	0.783	0.001
Dorsal Attention A: Parietal Occipital 1	3.5	0.008	0.239	2.8	0.046	0.000	0.666	0.889	0.058	2.6	0.114	0.037	0.5	0.463	0.008	1.2	0.281	0.017
Dorsal Attention A: Superior Parietal Lobule 1	5.5	0.001	0.330	4.6	0.005	0.000	0.657	0.061	0.001	2.0	0.166	0.028	0.2	0.663	0.003	2.1	0.153	0.030
Dorsal Attention B: Post Central 1	2.0	0.087	0.151	1.7	0.183	0.000				0.8	0.366	0.012	0.1	0.777	0.001	0.0	0.993	0.000
Dorsal Attention B: Post Central 2	2.6	0.032	0.191	3.0	0.037	0.000	0.715	0.173	0.007	0.4	0.538	0.006	0.6	0.445	0.009	2.4	0.123	0.035
Dorsal Attention B: Post Central 3	3.7	0.007	0.247	3.5	0.020	0.000	0.258	0.025	0.002	1.6	0.204	0.024	0.1	0.745	0.002	4.0	0.049	0.056
Dorsal Attention B: Frontal Eye Fields 1	3.1	0.016	0.215	1.1	0.346	0.000				0.1	0.728	0.002	0.4	0.541	0.006	1.6	0.204	0.024
Salience Ventral Attention A: Parietal Operculum 1	4.2	0.004	0.274	3.3	0.026	0.000	0.954	0.288	0.008	0.5	0.485	0.007	1.2	0.268	0.018	0.0	0.835	0.001
Salience Ventral Attention A: Insula: 1	3.5	0.008	0.239	2.0	0.120	0.000				0.2	0.656	0.003	0.2	0.628	0.004	1.6	0.212	0.023
Salience Ventral Attention A: Insula: 2	4.0	0.005	0.263	3.1	0.034	0.000	0.863	0.126	0.011	2.0	0.160	0.029	0.1	0.745	0.002	0.8	0.370	0.012
Salience Ventral Attention A: Parietal Medial 1	4.3	0.004	0.276	4.0	0.011	0.000	0.982	0.194	0.004	0.5	0.478	0.008	1.1	0.299	0.016	0.8	0.378	0.012
Salience Ventral Attention A: Frontal Medial 1	2.3	0.051	0.172	0.8	0.478	0.000				0.0	0.921	0.000	0.3	0.562	0.005	0.8	0.366	0.012
Salience Ventral Attention B: Lateral Prefrontal Cortex 1	3.6	0.008	0.242	1.9	0.135	0.000				2.9	0.092	0.042	0.1	0.800	0.001	0.2	0.692	0.002
Salience Ventral Attention B: Medial Posterior Prefrontal 1	4.7	0.002	0.297	5.7	0.001	0.000	0.894	0.027	0.000	0.0	0.979	0.000	0.6	0.437	0.009	2.6	0.110	0.038
LimbiC: B: Orbital Frontal Cortex 1	6.6	0.000	0.371	4.4	0.007	0.000	0.645	0.045	0.001	3.0	0.090	0.042	0.0	0.854	0.001	5.1	0.027	0.071
LimbiC: A: Temporal Pole 1	8.5	0.000	0.434	4.5	0.006	0.000	0.399	0.024	0.001	2.3	0.136	0.033	0.5	0.467	0.008	4.5	0.038	0.063
LimbiC: A: Temporal Pole 2	6.3	0.000	0.360	5.4	0.002	0.000	0.693	0.034	0.002	0.2	0.692	0.002	2.7	0.103	0.039	0.2	0.619	0.004
Control A: Intraparietal Sulcus 1	4.3	0.003	0.280	3.5	0.021	0.000	0.925	0.186	0.005	0.6	0.432	0.009	1.0	0.324	0.015	0.0	0.835	0.001
Control A: Lateral Prefrontal Cortex 1	4.3	0.004	0.277	3.3	0.026	0.000	0.842	0.193	0.007	3.0	0.090	0.042	0.1	0.784	0.001	1.2	0.287	0.017
Control A: Lateral Prefrontal Cortex 2	4.4	0.003	0.281	2.8	0.047	0.000	0.702	0.321	0.026	3.1	0.084	0.044	0.0	0.952	0.000	0.6	0.444	0.009
Control B: Lateral Prefrontal Cortex 1	8.0	0.000	0.418	4.6	0.005	0.000	0.417	0.105	0.001	8.3	0.005	0.111	0.3	0.573	0.005	1.6	0.217	0.023
Control C: Precuneus 1	3.5	0.008	0.240	3.6	0.017	0.000	0.339	0.112	0.002	0.8	0.364	0.012	0.2	0.627	0.004	1.8	0.186	0.026
Control C: Precuneus 2	2.6	0.035	0.187	3.2	0.027	0.000	0.331	0.121	0.003	0.6	0.455	0.008	0.0	0.883	0.000	2.5	0.115	0.037
Control C: Cingulate Posterior 1	4.9	0.002	0.306	5.2	0.003	0.000	0.840	0.270	0.001	1.2	0.268	0.018	0.5	0.467	0.008	1.8	0.180	0.027
Default A: Dorsal Prefrontal Cortex 1	3.7	0.007	0.248	2.3	0.083	0.000				1.7	0.197	0.025	0.0	0.897	0.000	0.0	0.836	0.001
Default A: Precuneus Posterior Cingulate Cortex1	2.7	0.027	0.197	2.6	0.058	0.000				1.1	0.294	0.016	0.1	0.709	0.002	0.1	0.816	0.001
Default A: Medial Prefrontal Cortex 1	5.7	0.001	0.337	5.5	0.002	0.000	0.866	0.100	0.001	1.1	0.292	0.017	0.0	0.921	0.000	0.3	0.616	0.004
Default B: Temp 1	4.5	0.003	0.287	1.5	0.220	0.000				1.4	0.248	0.020	0.2	0.668	0.003	1.5	0.219	0.022
Default B: Temp 2	3.1	0.015	0.217	3.1	0.034	0.000	0.761	0.257	0.009	1.1	0.295	0.016	0.1	0.709	0.002	1.8	0.185	0.026
Default B: Inferior Parietal Lobule 1	4.7	0.002	0.296	2.8	0.045	0.000	0.977	0.486	0.019	2.2	0.144	0.032	0.7	0.423	0.010	0.1	0.786	0.001
Default B: Dorsal Prefrontal Cortex 1	3.7	0.007	0.248	1.1	0.351	0.000				1.7	0.200	0.024	0.3	0.600	0.004	0.3	0.608	0.004
Default B: Lateral Prefrontal Cortex 1	2.6	0.033	0.190	1.6	0.204	0.000				2.1	0.150	0.031	0.0	0.979	0.000	0.8	0.369	0.012
Default B: Ventral Prefrontal Cortex 1	4.1	0.004	0.270	4.5	0.007	0.000	0.548	0.074	0.006	0.3	0.578	0.005	0.1	0.767	0.001	1.4	0.246	0.020
Default B: Ventral Prefrontal Cortex 2	8.1	0.000	0.421	5.3	0.002	0.000	0.836	0.043	0.000	3.1	0.081	0.045	0.0	0.904	0.000	0.1	0.767	0.001
Default C: Retro Superior Parietal Lobuleenial 1	3.4	0.009	0.235	1.4	0.260	0.000				1.7	0.194	0.025	0.5	0.479	0.008	1.7	0.196	0.025
Default C: Parahippocampal Cortex 1	2.4	0.044	0.178	1.8	0.152	0.000				0.1	0.790	0.001	0.2	0.662	0.003	1.0	0.320	0.015
Temporal Parietal 1	2.3	0.052	0.171	2.7	0.056	0.000				1.1	0.297	0.016	0.2	0.671	0.003	1.0	0.323	0.015

Right Hemisphere

	Overall			4 Groups: Age Cat and HOMAIR2 Median Split			Post-Hoc Contrasts			Systolic BP			Diastolic BP			Cortical Thickness		
	F	p-FDR	η^2_p	F	p	η^2_p	YIS vs YIR	YIS vs OIS	YIS vs OIR	F	p	η^2_p	F	p	η^2_p	F	p	η^2_p
Visual Central: Extra Striate Cortex 1	1.0	0.450	0.080	1.0	0.420	0.000				0.3	0.557	0.005	0.0	0.998	0.000	0.0	0.888	0.000
Visual Central: Extra Striate Cortex 2	4.4	0.003	0.282	3.9	0.012	0.000	0.809	0.288	0.004	0.6	0.425	0.010	1.2	0.281	0.017	0.2	0.678	0.003
Visual Central: Extra Striate Cortex 3	3.9	0.005	0.259	3.3	0.025	0.000	0.740	0.734	0.014	3.2	0.077	0.046	0.1	0.730	0.002	1.0	0.328	0.014
Visual Peripheral: Striate Cortex Calcarine 1	1.2	0.306	0.100	1.0	0.377	0.000				0.8	0.368	0.012	0.0	0.941	0.000	0.1	0.786	0.001
Visual Peripheral: Extra Striate Inferior 1	1.0	0.410	0.086	1.0	0.412	0.000				0.9	0.337	0.014	0.0	0.948	0.000	0.1	0.702	0.002
Visual Peripheral: Extra Striate Superior 1	2.0	0.087	0.150	2.1	0.105	0.000				0.6	0.439	0.009	0.3	0.586	0.004	0.2	0.622	0.004
Somatomotor A: 1	2.5	0.038	0.184	2.0	0.127	0.000				0.3	0.573	0.005	0.5	0.489	0.007	4.0	0.051	0.056
Somatomotor A: 2	2.0	0.083	0.153	1.5	0.216	0.000				0.1	0.751	0.002	0.5	0.492	0.007	1.7	0.191	0.025
Somatomotor A: 3	2.4	0.047	0.176	1.3	0.283	0.000				0.9	0.343	0.013	0.0	0.849	0.001	0.1	0.737	0.002
Somatomotor A: 4	2.0	0.079	0.155	1.1	0.370	0.000				0.1	0.781	0.001	0.4	0.530	0.006	1.1	0.294	0.016
Somatomotor B: Auditory 1	3.0	0.017	0.212	2.0	0.119	0.000				2.5	0.118	0.036	0.0	0.879	0.000	0.5	0.482	0.007
Somatomotor B: S2 1	2.6	0.034	0.188	1.6	0.208	0.000				2.1	0.156	0.030	0.1	0.815	0.001	0.0	1.000	0.000
Somatomotor B: S2 2	3.7	0.007	0.249	1.1	0.337	0.000				3.4	0.070	0.048	0.4	0.523	0.006	2.9	0.091	0.042
Somatomotor B: Central 1	2.3	0.056	0.169	1.9	0.136	0.000				1.6	0.208	0.024	0.0	0.960	0.000	0.4	0.534	0.006
Dorsal Attention A: Temporal Occipital 1	3.7	0.007	0.249	2.4	0.074	0.000				0.9	0.344	0.013	1.2	0.284	0.017	0.1	0.779	0.001
Dorsal Attention A: Parietal Occipital 1	2.9	0.019	0.208	2.5	0.069	0.000				1.0	0.327	0.014	0.7	0.398	0.011	0.0	0.856	0.000
Dorsal Attention A: Superior Parietal Lobule 1	4.0	0.005	0.264	4.2	0.009	0.000	0.400	0.174	0.002	0.7	0.420	0.010	0.7	0.403	0.010	1.1	0.302	0.016
Dorsal Attention B: Post Central 1	3.1	0.015	0.218	2.8	0.047	0.000	0.736	0.473	0.019	2.3	0.136	0.033	0.2	0.674	0.003	1.6	0.208	0.024
Dorsal Attention B: Post Central 2	2.8	0.024	0.202	2.7	0.052	0.000				0.4	0.553	0.005	0.4	0.521	0.006	1.1	0.295	0.016
Dorsal Attention B: Frontal Eye Fields 1	3.2	0.013	0.224	2.5	0.068	0.000				0.1	0.792	0.001	0.3	0.615	0.004	0.3	0.614	0.004
Salience Ventral Attention A: Parietal Operculum 1	3.9	0.005	0.260	2.3	0.086	0.000				2.2	0.144	0.032	0.0	0.943	0.000	0.9	0.334	0.014
Salience Ventral Attention A: Insula: 1	3.6	0.008	0.243	1.8	0.157	0.000				1.7	0.201	0.024	0.0	0.919	0.000	0.3	0.583	0.005
Salience Ventral Attention A: Parietal Medial 1	3.4	0.010	0.231	3.3	0.024	0.000	0.687	0.446	0.015	0.8	0.382	0.011	0.6	0.425	0.010	1.8	0.182	0.026
Salience Ventral Attention A: Frontal Medial 1	3.2	0.013	0.224	1.7	0.166	0.000				0.6	0.455	0.008	0.4	0.526	0.006	0.0	0.943	0.000
Salience Ventral Attention B: Inferior Parietal Lobule 1	4.2	0.004	0.272	1.7	0.168	0.000				1.5	0.218	0.023	0.2	0.634	0.003	0.9	0.336	0.014
Salience Ventral Attention B: Lateral Prefrontal Cortex 1	4.2	0.004	0.272	0.9	0.460	0.000				2.9	0.096	0.041	0.0	0.861	0.000	2.0	0.161	0.029
Salience Ventral Attention B: Medial Posterior Prefrontal 1	4.7	0.002	0.294	4.9	0.004	0.000	0.995	0.065	0.001	0.6	0.453	0.008	0.1	0.721	0.002	3.7	0.059	0.052
LimbiC: B: Orbital Frontal Cortex 1	5.4	0.001	0.325	3.3	0.025	0.000	0.402	0.084	0.003	3.6	0.062	0.051	0.2	0.658	0.003	0.9	0.355	0.013
LimbiC: A: Temporal Pole 1	8.9	0.000	0.444	6.4	0.001	0.000	0.049	0.001	0.000	2.0	0.162	0.029	0.3	0.564	0.005	1.2	0.285	0.017
Control A: Intraparietal Sulcus 1	4.1	0.004	0.266	2.5	0.069	0.000				1.8	0.187	0.026	1.1	0.309	0.015	0.0	0.913	0.000
Control A: Lateral Prefrontal Cortex 1	4.8	0.002	0.302	2.7	0.051	0.000				3.3	0.072	0.047	0.0	0.958	0.000	0.0	0.947	0.000
Control A: Lateral Prefrontal Cortex 2	5.2	0.001	0.316	2.4	0.072	0.000				0.6	0.445	0.009	0.4	0.544	0.006	1.8	0.184	0.026
Control B: Temporal 1	6.2	0.000	0.357	3.0	0.035	0.000	0.288	0.071	0.004	3.2	0.079	0.045	0.0	0.932	0.000	0.9	0.359	0.013
Control B: inferior parietal lobule 1	3.1	0.014	0.220	2.4	0.078	0.000				0.8	0.364	0.012	0.8	0.388	0.011	0.0	0.833	0.001
Control B: Lateral Prefrontal Cortexd 1	3.5	0.009	0.237	1.5	0.222	0.000				1.0	0.326	0.014	0.1	0.758	0.001	0.4	0.543	0.006
Control B: Lateral Prefrontal Cortexv 1	7.1	0.000	0.388	3.0	0.038	0.000	0.297	0.249	0.006	9.1	0.004	0.120	0.3	0.617	0.004	0.0	0.827	0.001
Control C: Cingulate Posterior 1	4.2	0.004	0.273	4.5	0.006	0.000	0.882	0.819	0.008	0.6	0.425	0.010	0.9	0.355	0.013	0.0	0.835	0.001
Control C: Precuneus 1	2.6	0.034	0.189	2.7	0.050	0.000				0.2	0.632	0.003	1.1	0.295	0.016	0.0	0.917	0.000
Default A: Inferior Parietal Lobule 1	3.2	0.014	0.220	2.4	0.078	0.000				1.2	0.283	0.017	0.2	0.645	0.003	0.0	0.916	0.000
Default A: Dorsal Prefrontal Cortex 1	4.6	0.002	0.292	3.4	0.024	0.000	0.573	0.082	0.003	0.4	0.548	0.005	0.2	0.629	0.004	0.2	0.666	0.003
Default A: Precuneus Posterior Cingulate Cortex 1	2.4	0.047	0.175	2.6	0.057	0.000				0.5	0.488	0.007	0.7	0.402	0.010	0.1	0.793	0.001
Default A: Medial Prefrontal Cortex 1	5.0	0.002	0.307	2.7	0.050	0.000				3.2	0.077	0.046	0.3	0.561	0.005	0.7	0.413	0.010
Default B: Dorsal Prefrontal Cortex 1	4.6	0.003	0.290	1.4	0.243	0.000				1.7	0.197	0.025	0.1	0.795	0.001	0.5	0.479	0.008
Default B: Ventral Prefrontal Cortex 1	5.2	0.001	0.318	3.3	0.027	0.000	0.774	0.162	0.006	2.8	0.101	0.040	0.0	0.982	0.000	0.0	0.900	0.000
Default B: Ventral Prefrontal Cortex 2	5.8	0.001	0.343	3.3	0.025	0.000	0.286	0.159	0.004	2.6	0.111	0.037	0.0	0.932	0.000	0.4	0.512	0.006
Default C: Retro Superior Parietal Lobuleenial 1	2.2	0.062	0.164	1.2	0.317	0.000				1.0	0.312	0.015	0.4	0.513	0.006	0.7	0.414	0.010
Default C: Parahippocampal Cortex 1	3.3	0.012	0.228	2.9	0.040	0.000	0.276	0.043	0.005	0.4	0.536	0.006	0.3	0.609	0.004	0.7	0.412	0.010
Temporal Parietal 1	4.7	0.002	0.295	3.8	0.014	0.000	0.944	0.099	0.003	0.7	0.423	0.010	0.2	0.627	0.004	0.0	0.924	0.000
Temporal Parietal 2	3.9	0.005	0.257	2.5	0.068	0.000				2.7	0.108	0.038	0.0	0.962	0.000	0.0	0.994	0.000
Temporal Parietal 3	2.4	0.047	0.176	1.9	0.131	0.000				1.3	0.251	0.020	0.3	0.600	0.004	0.4	0.546	0.005

YIS = younger insulin sensitive; YIR = younger insulin resistant; OIS = older insulin sensitive; OIR = older insulin resistant

2.3 The Effect of Age and Insulin Resistance on Regional CBF-CMR_{GLU} Associations

Table S6. Mean and standard deviation (SD) of cerebral metabolic rates of glucose (mg/100ml/min) among the four subgroups based on age and HOMA-IR.

	Left Hemisphere									Right Hemisphere							
	Younger Insulin Sensitive		Younger Insulin Resistant		Odler Insulin Sensitive		Older Insulin Resistsant			Younger Insulin Sensitive		Younger Insulin Resistant		Odler Insulin Sensitive		Older Insulin Resistant	
	Mean	SD	Mean	SD	Mean	SD	Mean	SD		Mean	SD	Mean	SD	Mean	SD	Mean	SD
Visual Central: Extra Striate Cortex 1	3.9	0.7	3.5	1.0	3.5	0.6	3.3	0.5	Visual Central: Extra Striate Cortex 1	3.9	0.6	3.5	1.0	3.5	0.6	3.4	0.4
Visual Central: Extra Striate Cortex 2	3.8	0.8	3.4	1.0	3.4	0.8	3.3	0.6	Visual Central: Extra Striate Cortex 2	4.1	0.9	3.5	1.0	3.6	0.9	3.4	0.7
Visual Central: Striate Cortex 1	4.4	1.0	3.8	1.1	3.7	0.8	3.5	0.7	Visual Central: Extra Striate Cortex 3	3.6	0.7	3.0	0.8	3.0	0.5	2.9	0.4
Visual Central: Extra Striate Cortex 3	3.5	0.7	3.1	0.8	2.9	0.5	2.9	0.4	Visual Peripheral: Striate Cortex Calcarine 1	4.5	1.0	3.9	1.0	3.8	0.8	3.7	0.6
Visual Peripheral: Extra Striate Inferior 1	4.1	0.6	3.8	0.9	3.4	0.6	3.3	0.4	Visual Peripheral: Extra Striate Inferior 1	3.6	0.8	3.3	0.9	2.9	0.5	2.8	0.3
Visual Peripheral: Striate Cortex Calcarine 1	4.0	1.1	3.5	1.0	3.2	0.6	3.2	0.4	Visual Peripheral: Extra Striate Superior 1	3.6	0.7	3.1	0.9	2.9	0.6	2.8	0.4
Visual Peripheral: Extra Striate Cortex Sup 1	3.9	0.7	3.4	0.9	3.1	0.6	3.0	0.5									
Somatomotor A: 1	3.6	0.6	3.2	0.8	2.8	0.5	2.7	0.4	Somatomotor A: 1	4.3	0.8	3.8	0.9	3.3	0.7	3.1	0.4
Somatomotor A: 2	3.5	0.6	3.0	0.7	2.7	0.6	2.5	0.4	Somatomotor A: 2	3.5	0.6	3.2	0.8	2.8	0.6	2.7	0.4
Somatomotor B: Auditory 1	4.0	0.6	3.5	0.9	3.2	0.4	3.0	0.4	Somatomotor A: 3	3.1	0.6	2.7	0.7	2.4	0.6	2.2	0.3
Somatomotor B: S2 1	4.4	0.8	3.8	0.9	3.5	0.5	3.3	0.4	Somatomotor A: 4	3.5	0.6	3.1	0.7	2.8	0.6	2.5	0.4
Somatomotor B: S2 2	4.1	0.6	3.5	0.9	2.9	0.5	2.8	0.4	Somatomotor B: Auditory 1	3.9	0.5	3.3	0.8	3.0	0.4	2.9	0.4
Somatomotor B: Central 1	3.7	0.6	3.3	0.8	3.0	0.5	2.9	0.4	Somatomotor B: S2 1	4.2	0.6	3.7	0.9	3.2	0.5	3.0	0.4
									Somatomotor B: S2 2	4.1	0.6	3.4	1.0	2.9	0.6	2.6	0.4
									Somatomotor B: Central 1	3.6	0.6	3.1	0.8	2.9	0.5	2.8	0.4
Dorsal Attention A: Temporal Occipital 1	3.7	0.6	3.3	0.9	3.1	0.4	2.9	0.4	Dorsal Attention A: Temporal Occipital 1	3.5	0.5	3.1	0.8	2.9	0.4	2.9	0.4
Dorsal Attention A: Parietal Occipital 1	3.4	0.7	3.1	0.9	2.8	0.3	2.7	0.4	Dorsal Attention A: Parietal Occipital 1	3.7	0.6	3.2	0.9	2.9	0.5	2.9	0.4
Dorsal Attention A: Superior Parietal Lobule 1	3.7	0.6	3.2	0.9	2.9	0.6	2.7	0.5	Dorsal Attention A: Superior Parietal Lobule 1	3.3	0.5	2.8	0.7	2.6	0.5	2.4	0.3
Dorsal Attention B: Post Central 1	3.4	0.6	3.0	0.7	2.6	0.5	2.4	0.4	Dorsal Attention B: Post Central 1	3.5	0.7	3.0	0.8	2.6	0.5	2.5	0.4
Dorsal Attention B: Post Central 2	3.2	0.9	2.8	0.7	2.4	0.6	2.2	0.4	Dorsal Attention B: Post Central 2	3.2	0.6	2.9	0.8	2.6	0.6	2.4	0.4
Dorsal Attention B: Post Central 3	3.1	0.7	2.7	0.7	2.4	0.5	2.2	0.4	Dorsal Attention B: Frontal Eye Fields 1	3.6	0.7	3.2	0.8	2.7	0.6	2.3	0.3
Dorsal Attention B: Frontal Eye Fields 1	3.7	0.7	3.4	0.8	2.8	0.5	2.5	0.4									
Salience Ventral Attention A: Parietal Operculum 1	4.0	0.7	3.5	0.9	3.0	0.5	2.8	0.5	Salience Ventral Attention A: Parietal Operculum 1	4.0	0.8	3.4	0.9	2.9	0.4	2.7	0.4
Salience Ventral Attention A: Insula: 1	3.2	0.5	2.9	0.7	2.3	0.4	2.2	0.3	Salience Ventral Attention A: Insula: 1	4.2	0.6	3.7	0.9	3.1	0.5	2.9	0.4
Salience Ventral Attention A: Insula: 2	4.8	0.8	4.3	1.1	3.5	0.7	3.3	0.4	Salience Ventral Attention A: Parietal Medial 1	4.1	0.6	3.6	0.8	3.3	0.6	2.9	0.4
Salience Ventral Attention A: Parietal Medial 1	4.0	0.6	3.4	0.9	3.0	0.6	2.7	0.3	Salience Ventral Attention A: Frontal Medial 1	4.1	0.8	3.7	0.9	3.1	0.7	2.7	0.5
Salience Ventral Attention A: Frontal Medial 1	4.2	0.8	3.8	0.9	3.2	0.7	2.8	0.5	Salience Ventral Attention B: Inferior Parietal Lobule 1	3.8	0.7	3.2	0.8	2.8	0.5	2.6	0.5
Salience Ventral Attention B: Lateral Prefrontal Cortex 1	4.5	0.8	4.0	1.0	3.3	0.6	2.9	0.5	Salience Ventral Attention B: Lateral Prefrontal Cortex 1	4.6	0.8	4.0	1.0	3.4	0.6	3.1	0.5
Salience Ventral Attention B: Medial Posterior Prefrontal 1	3.6	0.6	3.3	0.9	2.7	0.6	2.3	0.3	Salience Ventral Attention B: Medial Posterior Prefrontal 1	3.7	0.8	3.5	0.8	2.7	0.7	2.3	0.4
Limbic B: Orbital Frontal Cortex 1	4.5	0.7	4.0	1.1	3.5	0.5	3.2	0.5	Limbic B: Orbital Frontal Cortex 1	4.7	0.8	4.1	1.1	3.6	0.5	3.3	0.5
Limbic A: Temporal Pole 1	2.9	0.3	2.5	0.6	2.3	0.3	2.1	0.3	Limbic A: Temporal Pole 1	3.1	0.3	2.7	0.7	2.4	0.3	2.3	0.4
Limbic A: Temporal Pole 2	4.0	0.5	3.6	0.9	3.3	0.5	3.1	0.5									
Control A: Intraparietal Sulcus 1	3.9	0.8	3.5	0.9	2.9	0.6	2.7	0.5	Control A: Intraparietal Sulcus 1	3.2	0.6	2.8	0.7	2.5	0.5	2.4	0.4
Control A: Lateral Prefrontal Cortex 1	4.2	0.8	3.7	0.9	3.2	0.6	2.9	0.5	Control A: Lateral Prefrontal Cortex 1	4.5	0.8	3.8	1.0	3.2	0.5	3.0	0.5
Control A: Lateral Prefrontal Cortex 2	4.1	0.8	3.7	1.0	3.1	0.6	2.8	0.4	Control A: Lateral Prefrontal Cortex 2	4.0	0.8	3.4	0.9	2.9	0.5	2.7	0.4
Control B: Lateral Prefrontal Cortex 1	4.9	0.7	4.3	1.1	3.8	0.6	3.5	0.6	Control B: Temporal 1	4.1	0.5	3.5	0.9	3.2	0.5	2.9	0.5
Control C: Praecuneus 1	4.8	0.9	4.3	1.2	4.1	0.6	3.7	0.5	Control B: inferior parietal lobule 1	4.3	0.8	3.7	1.0	3.2	0.6	2.9	0.6
Control C: Praecuneus 2	4.0	0.7	3.5	0.8	3.3	0.7	3.0	0.5	Control B: Lateral Prefrontal Cortex 1	4.7	0.8	4.1	1.0	3.5	0.7	3.1	0.5
Control C: Cingulate Posterior 1	3.7	0.6	3.2	0.8	3.0	0.5	2.8	0.4	Control B: Lateral Prefrontal Cortexv 1	4.7	0.7	4.1	1.0	3.6	0.5	3.3	0.6
									Control C: Cingulate Posterior 1	4.1	0.6	3.6	0.8	3.3	0.6	2.9	0.4
									Control C: Precuneus 1	4.7	0.8	4.0	1.0	3.9	0.7	3.5	0.5
Default A: Dorsal Prefrontal Cortex 1	4.6	0.8	4.1	1.0	3.2	0.7	2.8	0.4	Default A: Inferior Parietal Lobule 1	3.7	0.6	3.2	0.8	2.9	0.5	2.6	0.5
Default A: Precuneus Posterior Cingulate Cortex1	4.8	0.7	4.2	1.0	4.0	0.7	3.6	0.5	Default A: Dorsal Prefrontal Cortex 1	4.2	0.9	3.8	1.0	3.0	0.6	2.7	0.4
Default A: Medial Prefrontal Cortex 1	4.1	0.6	3.8	0.9	3.0	0.5	2.8	0.4	Default A: Precuneus Posterior Cingulate Cortex 1	5.1	0.7	4.4	1.1	4.2	0.7	3.9	0.6
Default B: Temp 1	3.5	0.4	3.2	0.8	2.7	0.4	2.6	0.4	Default A: Medial Prefrontal Cortex 1	4.3	0.6	3.8	0.9	3.1	0.6	2.8	0.4
Default B: Temp 2	3.9	0.6	3.5	0.9	3.1	0.5	2.9	0.4	Default B: Dorsal Prefrontal Cortex 1	4.4	0.7	4.0	1.0	3.2	0.7	2.8	0.5
Default B: Inferior Parietal Lobule 1	3.8	0.6	3.4	0.9	3.0	0.5	2.8	0.5	Default B: Ventral Prefrontal Cortex 1	4.4	0.6	3.9	1.0	3.3	0.4	3.0	0.4
Default B: Dorsal Prefrontal Cortex 1	4.4	0.7	4.1	1.0	3.2	0.7	2.8	0.5	Default B: Ventral Prefrontal Cortex 2	4.8	0.7	4.2	1.1	3.5	0.6	3.2	0.5
Default B: Lateral Prefrontal Cortex 1	4.4	0.8	4.0	1.0	3.3	0.7	3.1	0.5	Default C: Retro Superior Parietal Lobuleenial 1	4.9	0.7	4.3	1.1	4.0	0.7	3.7	0.6
Default B: Ventral Prefrontal Cortex 1	4.4	0.6	3.9	1.0	3.3	0.5	3.0	0.4	Default C: Parahippocampal Cortex 1	3.3	0.4	2.9	0.7	2.7	0.4	2.6	0.3
Default B: Ventral Prefrontal Cortex 2	4.7	0.7	4.2	1.1	3.6	0.6	3.3	0.5									
Default C: Retro Superior Parietal Lobuleenial 1	5.1	0.7	4.4	1.2	4.1	0.6	3.6	0.7									
Default C: Parahippocampal Cortex 1	3.1	0.3	2.8	0.7	2.5	0.4	2.3	0.4									
Temporal Parietal 1	4.0	0.5	3.5	0.9	3.2	0.5	3.0	0.4	Temporal Parietal 1	3.0	0.3	2.7	0.7	2.2	0.3	2.2	0.3
									Temporal Parietal 2	3.9	0.5	3.4	0.9	2.9	0.4	2.8	0.4
									Temporal Parietal 3	3.5	0.6	3.1	0.8	2.8	0.4	2.6	0.4

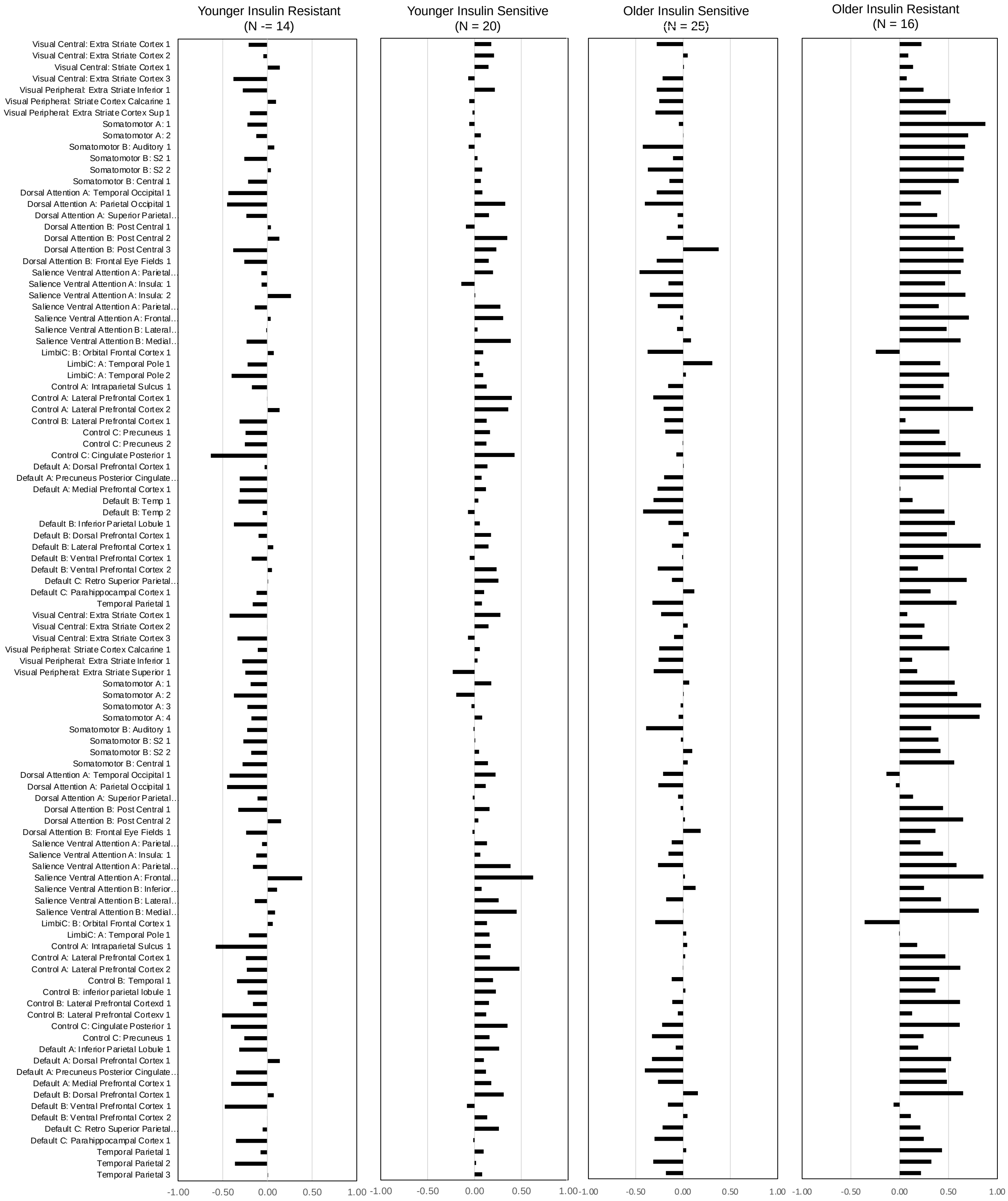

Figure S3. Partial correlation of regional CBF and CMR_{GLC} controlling cortical thickness and systolic and diastolic blood pressure for four groups of participants based on age category and HOMA-IR levels: younger insulin sensitive; younger insulin resistant; older insulin sensitive; and older insulin resistant. The data for each group are also plotted on the brain surface in Figure 4 in main document.

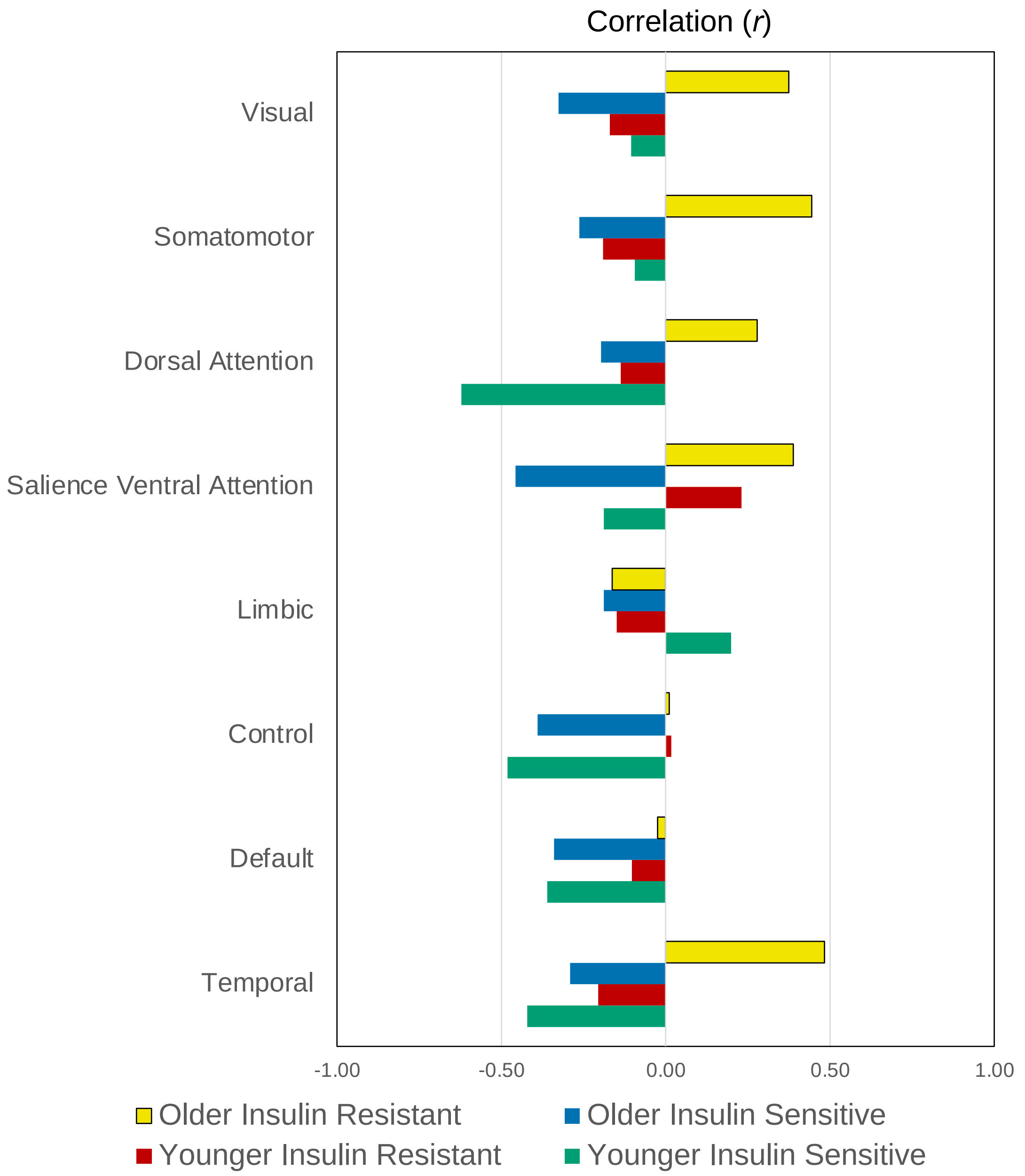

Figure S4. Partial correlation of network cerebral blood flow (CBF) and glucose metabolism (CMR_{GLC}) controlling for cortical thickness blood pressure, resting heart rate, BMI, sex and years of education. Four groups based on age category and HOMA-IR levels: younger insulin sensitive; younger insulin resistant; older insulin sensitive; and older insulin resistant

2.4 The Effect of Other Demographic Variables on CBF and CBF-CMR_{GLU} Association

Table S7. General linear models of the association between regional cerebral blood flow and age category, blood pressure, cortical thickness, resting heart rate, BMI, sex, years of education.

Left Hemisphere

	Overall Model			Age Category			Systolic BP			Diastolic BP			Resting HR			BMI			Years of Education			Sex		
	F	p-FDR	η^2_p	F	p	η^2_p	F	p	η^2_p	F	p	η^2_p	F	p	η^2_p	F	p	η^2_p	F	p	η^2_p	F	p	η^2_p
Visual Central: Extra Striate Cortex 1	4.8	0.000	0.417	0.6	0.461	0.010	0.0	0.999	0.000	2.3	0.131	0.042	0.2	0.679	0.003	0.4	0.537	0.007	5.9	0.018	0.099	14.2	0.000	0.208
Visual Central: Extra Striate Cortex 2	6.2	0.000	0.479	4.1	0.047	0.071	0.1	0.763	0.002	2.2	0.142	0.040	0.1	0.726	0.002	1.4	0.244	0.025	1.1	0.304	0.020	14.6	0.000	0.213
Visual Central: Striate Cortex 1	5.2	0.000	0.436	0.8	0.388	0.014	0.6	0.442	0.011	0.4	0.554	0.007	0.0	0.993	0.000	1.8	0.184	0.032	0.4	0.513	0.008	19.1	0.000	0.261
Visual Central: Extra Striate Cortex 3	5.2	0.000	0.433	1.8	0.184	0.032	1.6	0.217	0.028	0.1	0.721	0.002	0.1	0.723	0.002	3.7	0.060	0.064	0.5	0.491	0.009	14.2	0.000	0.209
Visual Peripheral: Extra Striate Inferior 1	3.5	0.003	0.339	0.3	0.603	0.005	0.0	0.858	0.001	0.1	0.762	0.002	0.1	0.718	0.002	1.7	0.198	0.030	2.6	0.115	0.045	14.6	0.000	0.212
Visual Peripheral: Striate Cortex Calcarine 1	3.9	0.002	0.364	0.0	0.854	0.001	0.3	0.613	0.005	0.0	0.976	0.000	0.9	0.352	0.016	3.7	0.061	0.064	1.7	0.202	0.030	17.7	0.000	0.247
Visual Peripheral: Extra Striate Cortex Sup 1	4.7	0.000	0.411	0.3	0.616	0.005	1.3	0.253	0.024	0.7	0.414	0.012	1.4	0.241	0.025	5.8	0.019	0.098	0.4	0.544	0.007	15.6	0.000	0.225
Somatomotor A: 1	3.3	0.004	0.329	0.8	0.374	0.015	0.0	0.921	0.000	0.1	0.789	0.001	0.8	0.390	0.014	0.2	0.625	0.004	0.2	0.680	0.003	14.9	0.000	0.216
Somatomotor A: 2	4.3	0.001	0.387	1.7	0.196	0.031	0.0	0.841	0.001	0.0	0.988	0.000	1.1	0.305	0.019	0.9	0.340	0.017	0.1	0.743	0.002	18.8	0.000	0.258
Somatomotor B: Auditory 1	2.4	0.029	0.260	0.6	0.428	0.012	0.2	0.634	0.004	0.4	0.542	0.007	1.1	0.303	0.020	3.3	0.075	0.057	2.3	0.134	0.041	6.7	0.012	0.111
Somatomotor B: S2 1	4.1	0.001	0.376	0.8	0.385	0.014	0.4	0.551	0.007	0.1	0.748	0.002	0.3	0.609	0.005	4.5	0.038	0.077	1.0	0.316	0.019	12.1	0.001	0.183
Somatomotor B: S2 2	2.8	0.011	0.297	1.2	0.282	0.021	0.6	0.445	0.011	0.2	0.628	0.004	0.3	0.586	0.006	3.1	0.086	0.054	1.2	0.283	0.021	7.5	0.008	0.122
Somatomotor B: Central 1	4.6	0.000	0.407	0.0	0.912	0.000	1.6	0.214	0.028	0.3	0.594	0.005	1.3	0.257	0.024	3.6	0.064	0.062	1.4	0.245	0.025	17.8	0.000	0.248
Dorsal Attention A: Temporal Occipital 1	5.7	0.000	0.456	2.1	0.149	0.038	0.1	0.718	0.002	0.6	0.429	0.012	0.2	0.698	0.003	4.3	0.043	0.074	2.6	0.111	0.046	13.9	0.000	0.205
Dorsal Attention A: Parietal Occipital 1	3.1	0.006	0.316	1.0	0.313	0.019	0.6	0.448	0.011	0.0	0.981	0.000	0.3	0.559	0.006	3.5	0.065	0.062	1.8	0.189	0.032	8.0	0.006	0.129
Dorsal Attention A: Superior Parietal Lobule 1	8.3	0.000	0.553	6.3	0.015	0.105	1.4	0.239	0.026	0.0	0.833	0.001	0.4	0.509	0.008	2.3	0.137	0.040	0.5	0.498	0.009	26.4	0.000	0.328
Dorsal Attention B: Post Central 1	3.3	0.005	0.328	0.9	0.335	0.017	0.7	0.410	0.013	0.7	0.404	0.013	0.6	0.459	0.010	1.6	0.216	0.028	0.5	0.470	0.010	12.3	0.001	0.185
Dorsal Attention B: Post Central 2	3.7	0.002	0.357	1.3	0.264	0.023	0.4	0.550	0.007	0.0	0.902	0.000	0.8	0.381	0.014	3.0	0.091	0.052	0.2	0.700	0.003	15.0	0.000	0.217
Dorsal Attention B: Post Central 3	5.5	0.000	0.451	5.4	0.025	0.090	0.8	0.379	0.014	0.6	0.448	0.011	1.7	0.199	0.030	0.9	0.352	0.016	0.0	0.882	0.000	18.4	0.000	0.254
Dorsal Attention B: Frontal Eye Fields 1	4.5	0.001	0.401	1.5	0.223	0.027	0.0	0.865	0.001	0.3	0.606	0.005	0.3	0.601	0.005	0.5	0.478	0.009	0.8	0.386	0.014	13.5	0.001	0.200
Salience Ventral Attention A: Parietal Operculum 1	5.4	0.000	0.445	3.7	0.059	0.065	0.2	0.682	0.003	0.4	0.526	0.007	0.0	0.985	0.000	2.3	0.138	0.040	1.0	0.319	0.018	15.4	0.000	0.222
Salience Ventral Attention A: Insula: 1	5.6	0.000	0.454	4.0	0.050	0.069	0.2	0.627	0.004	0.0	0.831	0.001	1.0	0.314	0.019	1.9	0.177	0.034	2.9	0.094	0.051	16.6	0.000	0.235
Salience Ventral Attention A: Insula: 2	5.1	0.000	0.430	1.7	0.200	0.030	1.3	0.256	0.024	1.2	0.286	0.021	0.5	0.481	0.009	4.5	0.039	0.077	0.8	0.373	0.015	12.4	0.001	0.187
Salience Ventral Attention A: Parietal Medial 1	5.4	0.000	0.444	1.4	0.243	0.025	1.3	0.258	0.024	0.0	0.950	0.000	0.4	0.553	0.007	5.1	0.028	0.086	0.0	0.945	0.000	12.2	0.001	0.185
Salience Ventral Attention A: Frontal Medial 1	3.4	0.004	0.335	1.0	0.319	0.018	0.9	0.360	0.016	1.1	0.294	0.020	0.1	0.736	0.002	0.9	0.360	0.016	0.2	0.658	0.004	12.5	0.001	0.188
Salience Ventral Attention B: Lateral Prefrontal Cort	5.7	0.000	0.456	3.0	0.091	0.052	0.9	0.339	0.017	0.2	0.649	0.004	0.5	0.493	0.009	0.7	0.405	0.013	0.5	0.495	0.009	20.7	0.000	0.277
Salience Ventral Attention B: Medial Posterior Prefro	5.9	0.000	0.468	6.2	0.016	0.103	0.0	0.870	0.000	0.0	0.961	0.000	0.1	0.711	0.003	2.0	0.160	0.036	0.2	0.662	0.004	16.3	0.000	0.232
LimbiC: B: Orbital Frontal Cortex 1	5.1	0.000	0.429	5.0	0.029	0.085	2.8	0.102	0.049	0.2	0.632	0.004	0.0	0.904	0.000	3.7	0.059	0.064	0.1	0.769	0.002	1.1	0.296	0.020
LimbiC: A: Temporal Pole 1	9.2	0.000	0.578	7.2	0.010	0.117	1.0	0.328	0.018	0.2	0.679	0.003	0.2	0.690	0.003	0.7	0.413	0.012	0.2	0.685	0.003	19.4	0.000	0.264
LimbiC: A: Temporal Pole 2	6.5	0.000	0.489	11.2	0.002	0.172	0.0	0.853	0.001	1.7	0.197	0.031	0.0	0.970	0.000	2.6	0.113	0.046	1.6	0.215	0.028	10.4	0.002	0.161
Control A: Intraparietal Sulcus 1	6.2	0.000	0.480	4.1	0.048	0.070	0.1	0.749	0.002	0.3	0.591	0.005	0.3	0.571	0.006	1.4	0.240	0.025	0.4	0.538	0.007	21.5	0.000	0.285
Control A: Lateral Prefrontal Cortex 1	6.4	0.000	0.485	0.6	0.432	0.011	1.7	0.196	0.031	0.4	0.519	0.008	0.1	0.781	0.001	3.9	0.052	0.068	0.1	0.745	0.002	19.0	0.000	0.260
Control A: Lateral Prefrontal Cortex 2	6.1	0.000	0.475	1.7	0.204	0.030	1.7	0.193	0.031	0.2	0.645	0.004	0.0	0.874	0.000	3.1	0.084	0.054	0.9	0.335	0.017	16.5	0.000	0.234
Control B: Lateral Prefrontal Cortex 1	7.0	0.000	0.508	3.2	0.081	0.055	6.5	0.014	0.107	1.1	0.303	0.020	0.2	0.681	0.003	2.1	0.151	0.038	0.3	0.569	0.006	8.5	0.005	0.136
Control C: Precuneus 1	5.3	0.000	0.442	2.3	0.135	0.041	0.7	0.413	0.012	0.0	0.878	0.000	0.4	0.515	0.008	3.3	0.073	0.058	0.7	0.404	0.013	18.4	0.000	0.254
Control C: Precuneus 2	4.6	0.000	0.404	2.6	0.115	0.045	0.2	0.649	0.004	0.2	0.660	0.004	0.8	0.367	0.015	4.8	0.033	0.082	0.3	0.565	0.006	16.8	0.000	0.237
Control C: Cingulate Posterior 1	6.3	0.000	0.481	0.9	0.342	0.017	0.7	0.418	0.012	0.0	0.983	0.000	0.0	0.981	0.000	5.3	0.025	0.089	0.1	0.738	0.002	18.2	0.000	0.252
Default A: Dorsal Prefrontal Cortex 1	4.7	0.000	0.408	3.6	0.065	0.062	0.1	0.726	0.002	0.0	0.840	0.001	0.5	0.475	0.010	0.3	0.604	0.005	0.5	0.475	0.009	15.0	0.000	0.217
Default A: Precuneus Posterior Cingulate Cortex1	4.3	0.001	0.391	0.3	0.589	0.005	0.7	0.417	0.012	0.1	0.704	0.003	0.4	0.513	0.008	6.8	0.012	0.112	0.0	0.835	0.001	14.1	0.000	0.207
Default A: Medial Prefrontal Cortex 1	5.3	0.000	0.438	7.5	0.008	0.122	0.5	0.483	0.009	0.4	0.512	0.008	0.5	0.476	0.009	3.2	0.080	0.056	0.0	0.846	0.001	8.6	0.005	0.138
Default B: Temp 1	5.7	0.000	0.456	1.2	0.274	0.022	0.0	0.989	0.000	0.3	0.578	0.006	0.1	0.802	0.001	1.5	0.223	0.027	0.8	0.368	0.015	16.6	0.000	0.235
Default B: Temp 2	3.3	0.004	0.329	2.3	0.132	0.041	0.0	0.877	0.000	0.0	0.995	0.000	0.7	0.403	0.013	4.1	0.049	0.070	1.0	0.319	0.018	9.7	0.003	0.153
Default B: Inferior Parietal Lobule 1	6.0	0.000	0.471	1.0	0.311	0.019	0.8	0.368	0.015	0.2	0.678	0.003	0.0	0.958	0.000	3.8	0.056	0.066	1.3	0.260	0.023	16.6	0.000	0.235
Default B: Dorsal Prefrontal Cortex 1	4.6	0.000	0.405	4.1	0.048	0.070	0.1	0.716	0.002	0.1	0.709	0.003	0.3	0.599	0.005	0.7	0.413	0.012	2.0	0.159	0.036	9.8	0.003	0.154
Default B: Lateral Prefrontal Cortex 1	3.6	0.002	0.351	1.9	0.176	0.034	0.5	0.486	0.009	0.0	0.958	0.000	0.1	0.769	0.002	1.1	0.293	0.020	1.8	0.181	0.033	11.2	0.001	0.172
Default B: Ventral Prefrontal Cortex 1	5.0	0.000	0.427	5.6	0.021	0.094	0.1	0.732	0.002	0.2	0.689	0.003	0.2	0.654	0.004	1.7	0.202	0.030	0.9	0.339	0.017	16.7	0.000	0.236
Default B: Ventral Prefrontal Cortex 2	8.5	0.000	0.559	3.6	0.064	0.062	3.1	0.083	0.055	0.6	0.430	0.012	0.0	0.996	0.000	5.8	0.020	0.096	0.7	0.401	0.013	11.9	0.001	0.181
Default C: Retro Superior Parietal Lobuleenial 1	5.1	0.000	0.432	1.3	0.264	0.023	0.2	0.655	0.004	0.1	0.818	0.001	1.9	0.177	0.033	2.9	0.093	0.051	1.6	0.205	0.030	16.8	0.000	0.237
Default C: Parahippocampal Cortex 1	3.7	0.002	0.353	3.9	0.055	0.067	0.0	0.887	0.000	0.0	0.827	0.001	0.4	0.528	0.007	3.2	0.079	0.056	2.9	0.092	0.052	7.8	0.007	0.126
Temporal Parietal 1	2.9	0.011	0.299	0.9	0.352	0.016	0.0	0.852	0.001	0.0	0.901	0.000	0.1	0.766	0.002	5.2	0.027	0.087	1.7	0.203	0.030	8.3	0.006	0.133

Continued …

… Table S7 continued

Right Hemisphere

	Overall Model			Age Category			Systolic BP			Diastolic BP			Resting HR			BMI			Years of Education			Sex		
	F	p-FDR	η^2_p	F	p	η^2_p	F	p	η^2_p	F	p	η^2_p	F	p	η^2_p	F	p	η^2_p	F	p	η^2_p	F	p	η^2_p
Visual Central: Extra Striate Cortex 1	2.6	0.019	0.277	0.1	0.734	0.002	0.0	0.880	0.000	0.0	0.836	0.001	0.0	0.899	0.000	2.2	0.144	0.039	1.0	0.317	0.019	11.1	0.002	0.170
Visual Central: Extra Striate Cortex 2	3.8	0.002	0.358	4.0	0.050	0.069	0.0	0.880	0.000	1.1	0.301	0.020	0.3	0.582	0.006	1.5	0.230	0.027	0.3	0.560	0.006	7.9	0.007	0.127
Visual Central: Extra Striate Cortex 3	4.6	0.000	0.406	0.9	0.357	0.016	0.5	0.464	0.010	0.0	0.914	0.000	0.0	0.897	0.000	2.5	0.121	0.044	0.1	0.802	0.001	16.2	0.000	0.230
Visual Peripheral: Striate Cortex Calcarine 1	2.3	0.035	0.253	0.9	0.354	0.016	0.0	0.900	0.000	0.0	0.940	0.000	1.2	0.282	0.021	1.7	0.193	0.031	0.4	0.517	0.008	9.8	0.003	0.153
Visual Peripheral: Extra Striate Inferior 1	2.0	0.062	0.230	0.1	0.743	0.002	0.0	0.880	0.000	0.0	0.878	0.000	1.0	0.312	0.019	1.7	0.201	0.030	1.1	0.308	0.019	9.4	0.003	0.148
Visual Peripheral: Extra Striate Superior 1	3.3	0.005	0.326	2.1	0.158	0.037	0.1	0.782	0.001	0.0	0.885	0.000	1.1	0.306	0.019	4.4	0.040	0.076	1.7	0.203	0.030	9.9	0.003	0.155
Somatomotor A: 1	3.1	0.006	0.317	0.2	0.647	0.004	0.0	0.957	0.000	0.0	0.865	0.001	0.1	0.717	0.002	4.2	0.045	0.072	1.6	0.213	0.029	7.9	0.007	0.128
Somatomotor A: 2	3.6	0.003	0.348	0.6	0.444	0.011	0.0	0.996	0.000	0.0	0.832	0.001	0.4	0.533	0.007	1.3	0.258	0.024	0.0	0.873	0.000	15.9	0.000	0.227
Somatomotor A: 3	4.7	0.000	0.410	1.0	0.329	0.018	1.6	0.210	0.029	0.6	0.439	0.011	1.7	0.199	0.030	2.3	0.134	0.041	0.2	0.691	0.003	16.1	0.000	0.230
Somatomotor A: 4	3.6	0.003	0.347	0.6	0.453	0.010	0.0	0.963	0.000	0.2	0.694	0.003	0.6	0.455	0.010	0.1	0.799	0.001	0.4	0.530	0.007	16.2	0.000	0.231
Somatomotor B: Auditory 1	4.1	0.001	0.380	0.1	0.809	0.001	0.7	0.414	0.012	0.4	0.513	0.008	0.3	0.566	0.006	6.3	0.015	0.105	0.1	0.769	0.002	12.6	0.001	0.189
Somatomotor B: S2 1	4.0	0.001	0.375	0.0	0.870	0.000	1.1	0.302	0.020	0.5	0.496	0.009	0.1	0.785	0.001	8.1	0.006	0.131	0.2	0.639	0.004	10.0	0.003	0.156
Somatomotor B: S2 2	4.7	0.000	0.408	0.0	0.899	0.000	1.4	0.246	0.025	0.4	0.519	0.008	0.0	0.927	0.000	6.4	0.014	0.106	0.0	0.870	0.001	8.8	0.004	0.140
Somatomotor B: Central 1	3.6	0.002	0.349	0.1	0.819	0.001	0.9	0.356	0.016	0.1	0.728	0.002	0.1	0.802	0.001	4.8	0.032	0.082	0.1	0.783	0.001	13.0	0.001	0.194
Dorsal Attention A: Temporal Occipital 1	5.3	0.000	0.440	1.9	0.179	0.033	0.0	0.888	0.000	1.9	0.171	0.034	0.1	0.810	0.001	1.7	0.197	0.031	0.0	0.893	0.000	17.8	0.000	0.248
Dorsal Attention A: Parietal Occipital 1	4.1	0.001	0.379	0.5	0.493	0.009	0.2	0.659	0.004	0.4	0.541	0.007	0.3	0.583	0.006	3.9	0.052	0.068	0.4	0.542	0.007	11.9	0.001	0.181
Dorsal Attention A: Superior Parietal Lobule 1	5.5	0.000	0.450	4.5	0.038	0.077	0.0	0.950	0.000	0.3	0.604	0.005	0.2	0.678	0.003	2.1	0.157	0.037	0.2	0.697	0.003	22.3	0.000	0.292
Dorsal Attention B: Post Central 1	4.2	0.001	0.385	0.9	0.356	0.016	2.3	0.133	0.041	0.3	0.568	0.006	0.3	0.594	0.005	6.4	0.014	0.106	0.0	0.996	0.000	11.6	0.001	0.176
Dorsal Attention B: Post Central 2	4.2	0.001	0.385	3.9	0.053	0.067	0.0	0.864	0.001	0.0	0.998	0.000	1.4	0.240	0.025	0.7	0.397	0.013	0.5	0.465	0.010	16.5	0.000	0.234
Dorsal Attention B: Frontal Eye Fields 1	4.8	0.000	0.418	4.2	0.045	0.072	0.1	0.811	0.001	0.0	0.983	0.000	0.2	0.678	0.003	0.7	0.413	0.012	0.2	0.668	0.003	17.5	0.000	0.245
Salience Ventral Attention A: Parietal Operculum 1	4.4	0.001	0.395	0.0	0.980	0.000	1.3	0.260	0.023	0.0	0.857	0.001	0.1	0.727	0.002	7.6	0.008	0.123	0.1	0.761	0.002	5.7	0.020	0.096
Salience Ventral Attention A: Insula: 1	5.5	0.000	0.448	0.0	0.919	0.000	0.9	0.344	0.017	0.5	0.478	0.009	0.3	0.617	0.005	6.6	0.013	0.109	0.0	0.887	0.000	12.9	0.001	0.193
Salience Ventral Attention A: Parietal Medial 1	3.4	0.004	0.337	0.8	0.363	0.015	1.3	0.251	0.024	0.1	0.737	0.002	0.4	0.550	0.007	2.4	0.128	0.042	0.0	0.845	0.001	9.6	0.003	0.151
Salience Ventral Attention A: Frontal Medial 1	5.4	0.000	0.445	0.8	0.385	0.014	0.0	0.893	0.000	0.5	0.490	0.009	0.0	0.867	0.001	0.8	0.381	0.014	0.1	0.769	0.002	20.2	0.000	0.272
Salience Ventral Attention B: Inferior Parietal Lobule	4.0	0.001	0.372	1.9	0.173	0.034	0.3	0.581	0.006	0.1	0.728	0.002	0.0	0.899	0.000	3.4	0.071	0.059	0.0	0.972	0.000	6.3	0.015	0.104
Salience Ventral Attention B: Lateral Prefrontal Cort	6.6	0.000	0.493	0.5	0.471	0.010	1.0	0.325	0.018	0.1	0.795	0.001	1.2	0.281	0.022	3.3	0.076	0.057	0.0	0.838	0.001	17.5	0.000	0.245
Salience Ventral Attention B: Medial Posterior Prefro	5.5	0.000	0.449	4.8	0.032	0.082	0.1	0.723	0.002	0.0	0.962	0.000	0.2	0.635	0.004	2.1	0.154	0.037	0.8	0.371	0.015	16.8	0.000	0.237
LimbiC: B: Orbital Frontal Cortex 1	6.2	0.000	0.477	2.7	0.109	0.047	6.4	0.014	0.106	1.7	0.193	0.031	0.0	0.843	0.001	4.8	0.033	0.081	1.4	0.238	0.026	1.9	0.172	0.034
LimbiC: A: Temporal Pole 1	9.1	0.000	0.573	7.9	0.007	0.128	0.4	0.511	0.008	0.5	0.470	0.010	0.1	0.801	0.001	1.1	0.304	0.020	1.7	0.202	0.030	13.3	0.001	0.197
Control A: Intraparietal Sulcus 1	5.3	0.000	0.439	2.3	0.136	0.041	0.4	0.535	0.007	0.1	0.728	0.002	0.1	0.738	0.002	2.7	0.107	0.047	0.1	0.703	0.003	17.3	0.000	0.243
Control A: Lateral Prefrontal Cortex 1	5.3	0.000	0.438	1.8	0.186	0.032	1.8	0.185	0.032	0.3	0.563	0.006	0.5	0.471	0.010	3.9	0.054	0.067	0.0	0.990	0.000	11.7	0.001	0.178
Control A: Lateral Prefrontal Cortex 2	6.7	0.000	0.500	1.3	0.266	0.023	0.2	0.689	0.003	0.1	0.751	0.002	0.1	0.749	0.002	5.7	0.021	0.095	0.0	0.862	0.001	10.3	0.002	0.160
Control B: Temporal 1	7.8	0.000	0.535	2.7	0.107	0.048	0.9	0.336	0.017	0.2	0.675	0.003	0.1	0.712	0.003	3.4	0.071	0.059	0.1	0.755	0.002	19.4	0.000	0.264
Control B: inferior parietal lobule 1	4.1	0.001	0.377	1.4	0.249	0.024	0.1	0.751	0.002	0.4	0.536	0.007	0.0	0.873	0.000	2.9	0.097	0.050	0.0	0.831	0.001	12.6	0.001	0.190
Control B: Lateral Prefrontal Cortexd 1	4.8	0.000	0.415	2.9	0.093	0.052	0.0	0.838	0.001	0.0	0.863	0.001	0.8	0.367	0.015	2.6	0.113	0.046	0.0	0.869	0.001	13.0	0.001	0.194
Control B: Lateral Prefrontal Cortexv 1	7.7	0.000	0.532	0.7	0.401	0.013	6.4	0.014	0.107	0.5	0.497	0.009	0.0	0.964	0.000	3.2	0.078	0.056	1.3	0.260	0.023	13.3	0.001	0.198
Control C: Cingulate Posterior 1	5.0	0.000	0.425	0.8	0.386	0.014	0.1	0.800	0.001	0.1	0.819	0.001	0.1	0.766	0.002	3.9	0.052	0.068	0.2	0.647	0.004	17.1	0.000	0.240
Control C: Precuneus 1	3.1	0.007	0.314	1.5	0.225	0.027	0.1	0.707	0.003	0.0	0.984	0.000	0.8	0.368	0.015	3.8	0.057	0.066	1.2	0.278	0.022	8.9	0.004	0.141
Default A: Inferior Parietal Lobule 1	5.0	0.000	0.427	0.2	0.688	0.003	0.5	0.470	0.010	0.0	0.889	0.000	0.3	0.614	0.005	6.4	0.014	0.106	0.0	0.933	0.000	13.3	0.001	0.198
Default A: Dorsal Prefrontal Cortex 1	5.5	0.000	0.447	6.2	0.016	0.103	0.1	0.823	0.001	0.1	0.820	0.001	0.7	0.391	0.014	1.3	0.255	0.024	0.2	0.647	0.004	17.1	0.000	0.241
Default A: Precuneus Posterior Cingulate Cortex 1	2.8	0.012	0.294	0.1	0.730	0.002	0.2	0.677	0.003	0.0	0.890	0.000	0.6	0.457	0.010	5.7	0.021	0.095	0.1	0.726	0.002	7.6	0.008	0.124
Default A: Medial Prefrontal Cortex 1	5.6	0.000	0.454	1.2	0.281	0.022	2.0	0.165	0.035	1.2	0.272	0.022	0.3	0.557	0.006	2.8	0.101	0.049	0.0	0.832	0.001	14.0	0.000	0.206
Default B: Dorsal Prefrontal Cortex 1	5.6	0.000	0.452	3.5	0.068	0.060	0.1	0.728	0.002	0.0	0.897	0.000	0.8	0.381	0.014	1.1	0.298	0.020	0.7	0.417	0.012	11.9	0.001	0.181
Default B: Ventral Prefrontal Cortex 1	4.8	0.000	0.413	3.9	0.052	0.068	1.4	0.241	0.025	0.2	0.638	0.004	0.1	0.711	0.003	2.8	0.100	0.049	1.2	0.285	0.021	4.2	0.046	0.072
Default B: Ventral Prefrontal Cortex 2	5.0	0.000	0.426	1.2	0.273	0.022	1.1	0.308	0.019	0.1	0.716	0.002	0.1	0.799	0.001	4.1	0.048	0.070	0.5	0.482	0.009	4.6	0.037	0.078
Default C: Retro Superior Parietal Lobuleenial 1	2.5	0.021	0.273	1.0	0.315	0.019	0.1	0.801	0.001	0.0	0.834	0.001	0.7	0.394	0.013	3.1	0.084	0.054	0.4	0.524	0.008	7.4	0.009	0.121
Default C: Parahippocampal Cortex 1	2.8	0.012	0.292	2.8	0.098	0.050	0.0	0.968	0.000	0.1	0.757	0.002	0.0	0.918	0.000	1.6	0.207	0.029	0.2	0.664	0.004	6.6	0.013	0.108
Temporal Parietal 1	4.8	0.000	0.416	5.9	0.019	0.098	0.0	0.874	0.000	0.1	0.701	0.003	0.1	0.724	0.002	1.3	0.257	0.024	0.2	0.622	0.005	14.1	0.000	0.207
Temporal Parietal 2	5.4	0.000	0.445	0.3	0.605	0.005	0.9	0.345	0.017	0.1	0.727	0.002	0.0	0.923	0.000	5.6	0.021	0.094	0.1	0.803	0.001	16.2	0.000	0.231
Temporal Parietal 3	3.7	0.002	0.353	0.3	0.607	0.005	0.2	0.622	0.005	0.1	0.775	0.002	0.0	0.852	0.001	4.3	0.044	0.073	0.0	0.983	0.000	12.8	0.001	0.191

Table S8. General linear models of the association between cerebral blood flow among the four sub-groups based on age category and HOMA-IR levels, with blood pressure, cortical thickness, resting heart rate, BMI, sex, years of education as covariates. Post-hoc contrasts in the regions with significant group difference are shown, comparing the young insulin sensitive group to the other three groups.

Left Hemisphere

	Overall			Systolic BP			Diastolic BP			Resting HR			BMI			Years of Education			Sex			Cortical Thickness			4 Groups: Age Category and HOMA Median Split			Post-Hoc Contrasts		
	F	p-FDR	η^2_p	F	p	η^2_p	F	p	η^2_p	F	p	η^2_p	F	p	η^2_p	F	p	η^2_p	F	p	η^2_p	F	p	η^2_p	F	p	η^2_p	YIS: vs YIR	YIS: vs OIS	YIS: vs OIR
Visual Central: Extra Striate Cortex 1	5.1	0.000	0.495	0.1	0.710	0.003	1.2	0.285	0.022	0.3	0.601	0.005	3.2	0.080	0.058	4.7	0.035	0.083	18.8	0.000	0.266	3.4	0.072	0.061	2.9	0.045	0.142	0.031	0.777	0.141
Visual Central: Extra Striate Cortex 2	5.6	0.000	0.518	0.5	0.468	0.010	2.5	0.123	0.045	0.0	0.837	0.001	2.8	0.101	0.051	0.2	0.621	0.005	16.0	0.000	0.235	0.9	0.359	0.016	2.8	0.047	0.141	0.047	0.949	0.792
Visual Central: Striate Cortex 1	5.2	0.000	0.498	1.4	0.236	0.027	0.2	0.644	0.004	0.6	0.443	0.011	4.9	0.031	0.087	0.1	0.821	0.001	23.2	0.000	0.308	0.0	0.961	0.000	2.4	0.077				
Visual Central: Extra Striate Cortex 3	4.2	0.001	0.449	2.1	0.157	0.038	0.0	0.899	0.000	0.3	0.609	0.005	3.6	0.063	0.065	0.1	0.740	0.002	13.8	0.000	0.210	0.0	0.876	0.000	1.1	0.362				
Visual Peripheral: Extra Striate Inferior 1	3.5	0.002	0.405	0.3	0.558	0.007	0.0	0.926	0.000	1.3	0.268	0.024	4.7	0.036	0.082	1.4	0.239	0.027	17.7	0.000	0.254	0.5	0.487	0.009	2.0	0.125				
Visual Peripheral: Striate Cortex Calcarine 1	3.6	0.002	0.406	0.7	0.397	0.014	0.0	0.979	0.000	2.1	0.151	0.039	5.8	0.019	0.101	0.7	0.397	0.014	19.3	0.000	0.271	4.1	0.048	0.073	1.3	0.297				
Visual Peripheral: Extra Striate Cortex Sup 1	4.0	0.001	0.435	2.0	0.161	0.037	0.3	0.574	0.006	1.9	0.171	0.036	6.1	0.017	0.104	0.0	0.857	0.001	15.6	0.000	0.231	0.7	0.395	0.014	0.8	0.490				
Somatomotor A: 1	2.8	0.009	0.347	0.0	0.882	0.000	0.4	0.532	0.008	0.4	0.523	0.008	0.0	0.865	0.001	0.0	0.845	0.001	13.3	0.001	0.204	1.8	0.185	0.034	0.8	0.521				
Somatomotor A: 2	3.4	0.003	0.392	0.0	0.948	0.000	0.0	0.926	0.000	1.2	0.273	0.023	1.0	0.317	0.019	0.0	0.896	0.000	18.0	0.000	0.257	1.2	0.269	0.023	0.7	0.548				
Somatomotor B: Auditory 1	1.9	0.074	0.263	0.2	0.651	0.004	0.2	0.645	0.004	0.7	0.403	0.013	2.1	0.152	0.039	1.9	0.170	0.036	5.9	0.019	0.101	0.1	0.777	0.002	0.3	0.839				
Somatomotor B: S2 1	3.2	0.004	0.381	0.4	0.537	0.007	0.0	0.936	0.000	0.1	0.707	0.003	3.1	0.085	0.056	0.7	0.424	0.012	10.6	0.002	0.170	0.7	0.398	0.014	0.4	0.757				
Somatomotor B: S2 2	2.2	0.032	0.300	0.7	0.412	0.013	0.2	0.651	0.004	0.4	0.526	0.008	3.0	0.091	0.054	0.8	0.371	0.015	7.3	0.009	0.123	0.1	0.786	0.001	0.5	0.717				
Somatomotor B: Central 1	4.0	0.001	0.435	2.2	0.144	0.041	0.0	0.909	0.000	1.3	0.263	0.024	2.8	0.099	0.052	0.5	0.468	0.010	16.7	0.000	0.243	0.8	0.374	0.015	0.8	0.478				
Dorsal Attention A: Temporal Occipital 1	5.2	0.000	0.501	0.4	0.555	0.007	0.2	0.638	0.004	1.2	0.285	0.022	8.0	0.007	0.133	2.0	0.163	0.037	16.9	0.000	0.245	0.4	0.523	0.008	2.3	0.088				
Dorsal Attention A: Parietal Occipital 1	2.5	0.016	0.327	0.8	0.363	0.016	0.0	0.898	0.000	0.5	0.491	0.009	3.3	0.075	0.060	1.0	0.316	0.019	7.7	0.008	0.129	0.1	0.701	0.003	0.6	0.611				
Dorsal Attention A: Superior Parietal Lobule 1	6.5	0.000	0.556	1.6	0.218	0.029	0.0	0.969	0.000	0.4	0.539	0.007	1.7	0.198	0.032	0.2	0.627	0.005	24.4	0.000	0.319	1.1	0.298	0.021	2.2	0.103				
Dorsal Attention B: Post Central 1	2.7	0.012	0.338	1.0	0.315	0.019	0.4	0.519	0.008	0.7	0.410	0.013	1.6	0.206	0.031	0.2	0.678	0.003	11.7	0.001	0.184	0.0	0.870	0.001	0.6	0.629				
Dorsal Attention B: Post Central 2	3.0	0.005	0.368	0.6	0.453	0.011	0.0	0.950	0.000	0.9	0.350	0.017	2.7	0.104	0.050	0.4	0.534	0.007	14.2	0.000	0.214	0.6	0.456	0.011	0.7	0.553				
Dorsal Attention B: Post Central 3	4.3	0.001	0.451	0.8	0.384	0.015	0.5	0.475	0.010	1.5	0.219	0.029	0.8	0.383	0.015	0.0	0.911	0.000	17.1	0.000	0.248	1.7	0.195	0.032	1.7	0.173				
Dorsal Attention B: Frontal Eye Fields 1	3.9	0.001	0.426	0.0	0.936	0.000	0.2	0.683	0.003	1.0	0.334	0.018	1.5	0.223	0.028	0.3	0.563	0.006	14.8	0.000	0.222	2.8	0.102	0.051	1.3	0.287				
Salience Ventral Attention A: Parietal Operculum 1	4.5	0.000	0.463	0.4	0.535	0.007	0.8	0.362	0.016	0.0	0.990	0.000	1.7	0.195	0.032	0.4	0.547	0.007	14.3	0.000	0.216	0.0	0.957	0.000	1.8	0.160				
Salience Ventral Attention A: Insula: 1	4.4	0.001	0.458	0.3	0.600	0.005	0.0	0.975	0.000	1.0	0.314	0.019	1.8	0.188	0.033	3.0	0.091	0.054	16.2	0.000	0.237	3.9	0.053	0.070	1.4	0.249				
Salience Ventral Attention A: Insula: 2	4.0	0.001	0.436	1.5	0.230	0.028	1.1	0.293	0.021	0.8	0.378	0.015	4.8	0.034	0.084	0.6	0.460	0.011	12.3	0.001	0.191	0.4	0.537	0.007	0.7	0.542				
Salience Ventral Attention A: Parietal Medial 1	5.0	0.000	0.490	2.3	0.134	0.043	0.2	0.630	0.005	0.6	0.448	0.011	5.3	0.026	0.092	0.3	0.565	0.006	12.8	0.001	0.198	0.5	0.502	0.009	2.1	0.115				
Salience Ventral Attention A: Frontal Medial 1	2.9	0.007	0.357	0.4	0.520	0.008	1.2	0.279	0.023	0.0	0.979	0.000	1.5	0.226	0.028	0.0	0.910	0.000	12.9	0.001	0.199	1.9	0.170	0.036	0.9	0.432				
Salience Ventral Attention B: Lateral Prefrontal Corte	4.6	0.000	0.472	1.4	0.245	0.026	0.1	0.702	0.003	1.0	0.328	0.018	1.3	0.258	0.025	0.1	0.713	0.003	21.0	0.000	0.288	0.0	0.997	0.000	1.5	0.222				
Salience Ventral Attention B: Medial Posterior Prefro	4.9	0.000	0.486	0.0	0.936	0.000	0.1	0.726	0.002	0.2	0.648	0.004	1.8	0.187	0.033	0.0	0.946	0.000	15.1	0.000	0.225	0.0	0.959	0.000	2.7	0.056				
LimbiC: B: Orbital Frontal Cortex 1	4.1	0.001	0.443	2.8	0.100	0.051	0.0	0.898	0.000	0.0	0.927	0.000	2.0	0.159	0.038	0.2	0.645	0.004	0.8	0.379	0.015	2.0	0.168	0.036	2.1	0.114				
LimbiC: A: Temporal Pole 1	7.1	0.000	0.578	0.9	0.345	0.017	0.2	0.660	0.004	0.1	0.734	0.002	0.5	0.497	0.009	0.2	0.679	0.003	17.9	0.000	0.256	4.5	0.038	0.080	2.3	0.087				
LimbiC: A: Temporal Pole 2	5.3	0.000	0.505	0.0	0.931	0.000	1.0	0.329	0.018	0.1	0.721	0.002	4.0	0.052	0.071	1.3	0.253	0.025	11.5	0.001	0.182	0.0	0.876	0.000	4.3	0.009	0.198	0.414	0.042	0.160
Control A: Intraparietal Sulcus 1	5.0	0.000	0.489	0.2	0.633	0.004	0.6	0.460	0.011	0.4	0.535	0.007	1.3	0.267	0.024	0.1	0.732	0.002	20.1	0.000	0.279	0.1	0.790	0.001	1.7	0.186				
Control A: Lateral Prefrontal Cortex 1	5.2	0.000	0.501	2.4	0.126	0.045	0.2	0.650	0.004	0.3	0.616	0.005	4.2	0.046	0.074	0.0	0.968	0.000	17.8	0.000	0.255	0.6	0.436	0.012	0.8	0.520				
Control A: Lateral Prefrontal Cortex 2	5.0	0.000	0.492	2.4	0.129	0.044	0.1	0.768	0.002	0.2	0.659	0.004	3.7	0.061	0.066	0.4	0.555	0.007	16.5	0.000	0.241	0.1	0.783	0.001	1.2	0.335				
Control B: Lateral Prefrontal Cortex 1	6.7	0.000	0.562	8.6	0.005	0.141	0.1	0.735	0.002	0.0	0.841	0.001	0.8	0.380	0.015	1.1	0.296	0.021	7.5	0.009	0.125	0.8	0.366	0.016	3.3	0.028	0.159	0.528	0.951	0.071
Control C: Precuneus 1	4.5	0.000	0.465	1.2	0.285	0.022	0.0	0.866	0.001	0.6	0.455	0.011	3.2	0.079	0.058	0.2	0.682	0.003	17.9	0.000	0.256	0.1	0.757	0.002	1.5	0.219				
Control C: Precuneus 2	3.6	0.002	0.407	0.3	0.616	0.005	0.1	0.769	0.002	0.7	0.418	0.013	3.6	0.064	0.064	0.2	0.667	0.004	15.1	0.000	0.225	0.9	0.347	0.017	0.9	0.444				
Control C: Cingulate Posterior 1	5.9	0.000	0.532	1.4	0.241	0.026	0.4	0.525	0.008	0.0	0.976	0.000	4.1	0.047	0.074	0.9	0.349	0.017	17.1	0.000	0.248	0.2	0.639	0.004	2.2	0.097				
Default A: Dorsal Prefrontal Cortex 1	3.9	0.001	0.431	0.4	0.547	0.007	0.0	0.877	0.000	1.1	0.289	0.022	0.9	0.359	0.016	0.1	0.711	0.003	15.6	0.000	0.231	0.7	0.395	0.014	1.9	0.146				
Default A: Precuneus Posterior Cingulate Cortex1	3.7	0.001	0.416	1.2	0.273	0.023	0.0	0.937	0.000	0.6	0.434	0.012	6.0	0.018	0.103	0.1	0.793	0.001	13.5	0.001	0.206	0.0	0.947	0.000	0.9	0.472				
Default A: Medial Prefrontal Cortex 1	4.8	0.000	0.481	0.9	0.352	0.017	0.0	0.942	0.000	0.3	0.619	0.005	1.7	0.201	0.031	0.0	0.828	0.001	7.1	0.010	0.120	0.3	0.558	0.007	4.0	0.012	0.189	0.627	0.350	0.014
Default B: Temp 1	4.5	0.000	0.463	0.0	0.897	0.000	0.1	0.736	0.002	0.0	0.853	0.001	1.3	0.262	0.024	1.1	0.294	0.021	16.3	0.000	0.239	4.8	0.033	0.084	0.6	0.604				
Default B: Temp 2	2.6	0.015	0.330	0.0	0.900	0.000	0.0	0.992	0.000	0.6	0.456	0.011	3.1	0.083	0.057	0.9	0.334	0.018	9.0	0.004	0.147	0.0	0.852	0.001	0.8	0.525				
Default B: Inferior Parietal Lobule 1	5.0	0.000	0.491	1.3	0.253	0.025	0.5	0.480	0.010	0.0	0.927	0.000	3.0	0.089	0.054	0.5	0.500	0.009	15.4	0.000	0.229	0.0	0.870	0.001	1.0	0.389				
Default B: Dorsal Prefrontal Cortex 1	3.8	0.001	0.423	0.3	0.571	0.006	0.1	0.713	0.003	0.8	0.387	0.014	1.4	0.240	0.026	1.2	0.269	0.023	10.5	0.002	0.168	0.3	0.607	0.005	1.9	0.142				
Default B: Lateral Prefrontal Cortex 1	3.1	0.005	0.371	1.0	0.327	0.019	0.0	0.981	0.000	0.4	0.524	0.008	1.7	0.192	0.033	0.9	0.351	0.017	11.4	0.001	0.180	0.1	0.724	0.002	1.2	0.323				
Default B: Ventral Prefrontal Cortex 1	4.1	0.001	0.438	0.0	0.897	0.000	0.1	0.717	0.003	0.5	0.478	0.010	2.2	0.140	0.041	0.5	0.497	0.009	16.6	0.000	0.242	0.2	0.625	0.005	2.2	0.101				
Default B: Ventral Prefrontal Cortex 2	7.3	0.000	0.583	4.5	0.038	0.080	0.2	0.684	0.003	0.1	0.802	0.001	5.6	0.022	0.097	1.8	0.184	0.034	11.6	0.001	0.183	0.0	0.847	0.001	2.2	0.096				
Default C: Retro Superior Parietal Lobuleenial 1	4.4	0.001	0.457	0.5	0.470	0.010	0.1	0.782	0.001	3.0	0.089	0.054	4.3	0.043	0.076	0.7	0.401	0.014	17.7	0.000	0.254	1.8	0.183	0.034	1.2	0.305				
Default C: Parahippocampal Cortex 1	3.9	0.001	0.426	0.0	0.886	0.000	0.0	0.840	0.001	2.1	0.156	0.038	7.6	0.008	0.128	2.4	0.130	0.043	11.0	0.002	0.175	0.8	0.370	0.015	3.6	0.019	0.172	0.052	0.496	0.654
Temporal Parietal 1	2.3	0.028	0.305	0.1	0.748	0.002	0.0	0.897	0.000	0.2	0.622	0.005	5.2	0.026	0.092	1.1	0.305	0.020	8.2	0.006	0.136	0.0	0.968	0.000	0.5	0.713				

Continued …

… Table S8 Continued
Right Hemisphere

	Overall			Systolic BP			Diastolic BP			Resting HR			BMI			Years of Education			Sex			Cortical Thickness			4 Groups: Age Category and HOMA Median Split			Post-Hoc Contrasts		
	F	p-FDR	η^2_p	F	p	η^2_p	F	p	η^2_p	F	p	η^2_p	F	p	η^2_p	F	p	η^2_p	F	p	η^2_p	F	p	η^2_p	F	p	η^2_p	YIS: vs YIR	YIS: vs OIS	YIS: vs OIR
Visual Central: Extra Striate Cortex 1	2.3	0.029	0.304	0.0	0.937	0.000	0.0	0.935	0.000	0.3	0.560	0.007	3.6	0.062	0.065	0.6	0.452	0.011	11.9	0.001	0.187	1.8	0.182	0.034	0.7	0.545				
Visual Central: Extra Striate Cortex 2	3.6	0.002	0.409	0.1	0.788	0.001	2.0	0.167	0.036	0.1	0.817	0.001	1.9	0.169	0.036	0.0	0.988	0.000	8.0	0.007	0.134	0.7	0.393	0.014	2.9	0.044	0.143	0.080	0.793	0.470
Visual Central: Extra Striate Cortex 3	4.2	0.001	0.444	1.1	0.301	0.021	0.3	0.614	0.005	0.0	0.962	0.000	2.4	0.124	0.045	0.1	0.791	0.001	16.0	0.000	0.235	0.1	0.781	0.002	1.5	0.232				
Visual Peripheral: Striate Cortex Calcarine 1	2.1	0.039	0.291	0.0	0.929	0.000	0.0	0.947	0.000	2.4	0.130	0.044	3.2	0.079	0.058	0.1	0.747	0.002	11.2	0.002	0.178	1.1	0.292	0.021	1.2	0.312				
Visual Peripheral: Extra Striate Inferior 1	1.7	0.108	0.245	0.1	0.727	0.002	0.0	0.904	0.000	1.5	0.220	0.029	2.2	0.144	0.041	0.5	0.471	0.010	9.5	0.003	0.155	0.1	0.775	0.002	0.4	0.761				
Visual Peripheral: Extra Striate Superior 1	3.0	0.005	0.369	0.5	0.500	0.009	0.0	0.905	0.000	1.8	0.191	0.033	5.0	0.029	0.088	0.5	0.462	0.010	10.0	0.003	0.162	1.2	0.286	0.022	1.9	0.143				
Somatomotor A: 1	2.6	0.014	0.333	0.0	0.904	0.000	0.2	0.669	0.004	0.1	0.742	0.002	3.2	0.081	0.057	0.8	0.368	0.016	7.1	0.010	0.120	4.5	0.038	0.080	0.5	0.690				
Somatomotor A: 2	2.8	0.008	0.353	0.0	0.943	0.000	0.1	0.709	0.003	0.3	0.592	0.006	0.9	0.351	0.017	0.1	0.770	0.002	14.4	0.000	0.217	2.6	0.111	0.048	0.3	0.799				
Somatomotor A: 3	3.9	0.001	0.427	2.1	0.149	0.040	0.5	0.500	0.009	2.5	0.122	0.045	3.2	0.081	0.057	0.4	0.520	0.008	16.5	0.000	0.241	0.2	0.669	0.004	0.8	0.475				
Somatomotor A: 4	3.0	0.005	0.369	0.1	0.795	0.001	0.3	0.593	0.006	1.0	0.317	0.019	0.3	0.594	0.005	0.1	0.778	0.002	16.5	0.000	0.241	1.9	0.177	0.035	0.8	0.500				
Somatomotor B: Auditory 1	3.3	0.003	0.390	0.9	0.334	0.018	0.2	0.646	0.004	0.5	0.501	0.009	5.7	0.021	0.099	0.0	0.975	0.000	12.0	0.001	0.188	3.3	0.076	0.059	0.3	0.826				
Somatomotor B: S2 1	3.2	0.004	0.379	1.2	0.274	0.023	0.4	0.543	0.007	0.2	0.679	0.003	7.5	0.009	0.126	0.1	0.769	0.002	9.6	0.003	0.156	2.0	0.167	0.036	0.1	0.945				
Somatomotor B: S2 2	3.6	0.002	0.411	1.5	0.228	0.028	0.3	0.573	0.006	0.0	0.854	0.001	5.6	0.021	0.098	0.1	0.788	0.001	8.4	0.005	0.139	5.1	0.028	0.089	0.1	0.975				
Somatomotor B: Central 1	2.9	0.007	0.360	1.1	0.303	0.020	0.0	0.942	0.000	0.1	0.793	0.001	3.9	0.054	0.069	0.3	0.616	0.005	12.0	0.001	0.188	1.0	0.327	0.018	0.3	0.805				
Dorsal Attention A: Temporal Occipital 1	4.4	0.001	0.456	0.0	0.902	0.000	1.6	0.211	0.030	0.0	0.860	0.001	2.7	0.104	0.050	0.0	0.903	0.000	18.3	0.000	0.261	1.0	0.320	0.019	1.1	0.339				
Dorsal Attention A: Parietal Occipital 1	3.6	0.002	0.412	0.6	0.443	0.011	0.7	0.391	0.014	0.1	0.803	0.001	4.2	0.045	0.075	0.0	0.901	0.000	12.1	0.001	0.188	0.1	0.743	0.002	1.1	0.348				
Dorsal Attention A: Superior Parietal Lobule 1	4.5	0.000	0.465	0.0	0.866	0.001	0.7	0.399	0.014	0.1	0.786	0.001	1.2	0.288	0.022	0.0	0.896	0.000	20.0	0.000	0.278	0.3	0.558	0.007	2.0	0.129				
Dorsal Attention B: Post Central 1	3.6	0.002	0.408	3.1	0.085	0.056	0.1	0.775	0.002	0.5	0.495	0.009	6.0	0.018	0.104	0.1	0.705	0.003	11.2	0.002	0.177	1.6	0.216	0.029	0.9	0.430				
Dorsal Attention B: Post Central 2	3.3	0.003	0.392	0.1	0.753	0.002	0.0	0.909	0.000	1.6	0.216	0.029	0.8	0.372	0.015	0.2	0.626	0.005	15.8	0.000	0.233	0.3	0.601	0.005	1.5	0.235				
Dorsal Attention B: Frontal Eye Fields 1	4.0	0.001	0.437	0.2	0.646	0.004	0.0	0.928	0.000	0.5	0.475	0.010	1.2	0.270	0.023	0.5	0.466	0.010	17.9	0.000	0.256	0.0	0.878	0.000	2.0	0.125				
Salience Ventral Attention A: Parietal Operculum 1	3.4	0.002	0.398	1.1	0.297	0.021	0.0	0.953	0.000	0.2	0.622	0.005	5.0	0.029	0.088	0.1	0.788	0.001	4.9	0.032	0.086	3.8	0.056	0.068	0.1	0.954				
Salience Ventral Attention A: Insula: 1	4.4	0.000	0.456	1.2	0.281	0.022	0.4	0.548	0.007	0.5	0.489	0.009	6.5	0.013	0.112	0.0	0.936	0.000	12.6	0.001	0.195	5.0	0.030	0.087	0.3	0.858				
Salience Ventral Attention A: Parietal Medial 1	3.3	0.003	0.388	2.3	0.133	0.043	0.0	0.914	0.000	0.7	0.417	0.013	2.6	0.112	0.048	0.1	0.702	0.003	9.7	0.003	0.158	0.1	0.804	0.001	1.7	0.172				
Salience Ventral Attention A: Frontal Medial 1	4.8	0.000	0.481	0.2	0.625	0.005	0.6	0.457	0.011	0.5	0.501	0.009	2.0	0.168	0.036	0.0	0.886	0.000	21.9	0.000	0.297	2.1	0.155	0.039	1.5	0.233				
Salience Ventral Attention B: Inferior Parietal Lobule	3.1	0.005	0.375	0.2	0.645	0.004	0.1	0.702	0.003	0.1	0.787	0.001	2.1	0.153	0.039	0.0	0.920	0.000	5.7	0.021	0.098	0.3	0.610	0.005	0.7	0.552				
Salience Ventral Attention B: Lateral Prefrontal Cort	5.4	0.000	0.511	1.4	0.243	0.026	0.1	0.763	0.002	2.1	0.153	0.039	4.6	0.037	0.081	0.0	0.965	0.000	18.6	0.000	0.264	2.9	0.095	0.053	0.8	0.503				
Salience Ventral Attention B: Medial Posterior Prefro	4.7	0.000	0.473	0.4	0.518	0.008	0.0	0.881	0.000	0.6	0.441	0.011	2.7	0.108	0.049	0.2	0.655	0.004	17.0	0.000	0.246	0.0	0.868	0.001	2.4	0.079				
LimbiC: B: Orbital Frontal Cortex 1	5.3	0.000	0.504	7.2	0.010	0.121	0.6	0.430	0.012	0.0	0.968	0.000	2.9	0.095	0.053	2.3	0.139	0.042	1.4	0.247	0.026	1.8	0.185	0.034	1.8	0.151				
LimbiC: A: Temporal Pole 1	7.1	0.000	0.578	0.2	0.660	0.004	0.5	0.482	0.010	0.0	0.984	0.000	0.4	0.510	0.008	1.2	0.271	0.023	12.2	0.001	0.190	6.1	0.017	0.104	2.8	0.050				
Control A: Intraparietal Sulcus 1	4.3	0.001	0.453	0.7	0.419	0.013	0.3	0.560	0.007	0.2	0.679	0.003	2.4	0.131	0.043	0.0	0.962	0.000	16.5	0.000	0.241	0.0	0.855	0.001	1.2	0.321				
Control A: Lateral Prefrontal Cortex 1	4.1	0.001	0.442	2.0	0.167	0.036	0.2	0.685	0.003	0.5	0.500	0.009	3.0	0.090	0.054	0.0	0.883	0.000	10.8	0.002	0.172	0.0	0.824	0.001	0.7	0.556				
Control A: Lateral Prefrontal Cortex 2	5.2	0.000	0.501	0.2	0.646	0.004	0.1	0.730	0.002	0.1	0.821	0.001	4.9	0.032	0.086	0.0	0.947	0.000	9.7	0.003	0.157	5.4	0.024	0.094	0.5	0.718				
Control B: Temporal 1	6.0	0.000	0.535	0.9	0.357	0.016	0.1	0.716	0.003	0.1	0.759	0.002	2.9	0.096	0.052	0.1	0.762	0.002	18.3	0.000	0.260	1.4	0.247	0.026	0.9	0.463				
Control B: inferior parietal lobule 1	3.3	0.003	0.385	0.2	0.686	0.003	0.6	0.424	0.012	0.0	0.842	0.001	1.8	0.181	0.034	0.0	0.987	0.000	11.4	0.001	0.179	0.0	0.904	0.000	0.7	0.577				
Control B: Lateral Prefrontal Cortexd 1	3.9	0.001	0.426	0.1	0.703	0.003	0.0	0.915	0.000	1.2	0.269	0.023	3.0	0.087	0.055	0.0	0.947	0.000	13.1	0.001	0.201	0.8	0.362	0.016	1.3	0.287				
Control B: Lateral Prefrontal Cortexv 1	6.6	0.000	0.558	7.4	0.009	0.125	0.0	0.874	0.000	0.0	0.934	0.000	2.2	0.143	0.041	1.9	0.169	0.036	12.4	0.001	0.192	0.0	0.997	0.000	1.3	0.300				
Control C: Cingulate Posterior 1	4.8	0.000	0.482	0.4	0.520	0.008	0.6	0.427	0.012	0.1	0.740	0.002	3.2	0.081	0.057	0.0	0.863	0.001	16.1	0.000	0.237	2.8	0.101	0.051	2.2	0.104				
Control C: Precuneus 1	2.8	0.009	0.350	0.5	0.489	0.009	0.1	0.741	0.002	1.0	0.321	0.019	3.5	0.068	0.063	0.4	0.528	0.008	8.3	0.006	0.137	0.0	0.867	0.001	1.5	0.235				
Default A: Inferior Parietal Lobule 1	4.0	0.001	0.432	0.7	0.416	0.013	0.1	0.747	0.002	0.2	0.653	0.004	4.8	0.032	0.085	0.0	0.908	0.000	12.2	0.001	0.190	0.5	0.465	0.010	0.2	0.873				
Default A: Dorsal Prefrontal Cortex 1	4.4	0.001	0.457	0.0	0.986	0.000	0.1	0.735	0.002	1.0	0.327	0.018	1.4	0.247	0.026	0.0	0.855	0.001	16.6	0.000	0.242	0.1	0.771	0.002	2.4	0.082				
Default A: Precuneus Posterior Cingulate Cortex 1	2.6	0.013	0.336	0.6	0.426	0.012	0.0	0.903	0.000	1.0	0.311	0.020	6.0	0.018	0.103	0.0	0.872	0.001	7.3	0.009	0.123	0.6	0.450	0.011	1.1	0.342				
Default A: Medial Prefrontal Cortex 1	4.9	0.000	0.484	3.0	0.091	0.054	0.4	0.517	0.008	0.4	0.512	0.008	2.4	0.125	0.045	0.4	0.516	0.008	13.5	0.001	0.206	0.5	0.482	0.010	1.4	0.251				
Default B: Dorsal Prefrontal Cortex 1	5.3	0.000	0.503	0.3	0.596	0.005	0.1	0.722	0.002	2.5	0.118	0.046	3.8	0.058	0.068	0.3	0.590	0.006	15.1	0.000	0.225	2.1	0.153	0.039	3.0	0.039	0.147	0.064	0.553	0.806
Default B: Ventral Prefrontal Cortex 1	4.2	0.001	0.444	2.5	0.123	0.045	0.0	0.849	0.001	0.3	0.584	0.006	2.6	0.111	0.048	2.1	0.158	0.038	3.8	0.057	0.068	0.1	0.701	0.003	2.3	0.087				
Default B: Ventral Prefrontal Cortex 2	4.2	0.001	0.445	1.4	0.248	0.026	0.0	0.987	0.000	0.1	0.745	0.002	2.6	0.112	0.048	0.9	0.343	0.017	3.8	0.056	0.069	2.3	0.139	0.042	1.0	0.397				
Default C: Retro Superior Parietal Lobuleenial 1	2.2	0.035	0.295	0.2	0.635	0.004	0.1	0.769	0.002	1.4	0.249	0.025	3.8	0.058	0.067	0.1	0.765	0.002	7.9	0.007	0.132	0.1	0.760	0.002	0.9	0.456				
Default C: Parahippocampal Cortex 1	2.3	0.025	0.309	0.1	0.800	0.001	0.1	0.768	0.002	0.2	0.657	0.004	2.4	0.131	0.043	0.0	0.850	0.001	7.0	0.011	0.118	0.4	0.538	0.007	1.4	0.261				
Temporal Parietal 1	3.8	0.001	0.422	0.0	1.000	0.000	0.1	0.704	0.003	0.3	0.595	0.005	1.6	0.212	0.030	0.1	0.762	0.002	13.9	0.000	0.211	0.1	0.814	0.001	2.1	0.114				
Temporal Parietal 2	4.3	0.001	0.452	1.2	0.287	0.022	0.0	0.839	0.001	0.0	0.958	0.000	5.1	0.027	0.090	0.0	0.986	0.000	15.5	0.000	0.229	0.7	0.395	0.014	0.3	0.817				
Temporal Parietal 3	3.0	0.006	0.365	0.5	0.505	0.009	0.2	0.648	0.004	0.0	0.969	0.000	3.9	0.054	0.069	0.1	0.797	0.001	12.2	0.001	0.190	0.0	0.896	0.000	0.4	0.736				

YIS = younger insulin sensitive; YIR = younger insulin resistant; OIS = older insulin sensitive; OIR = older insulin resistant

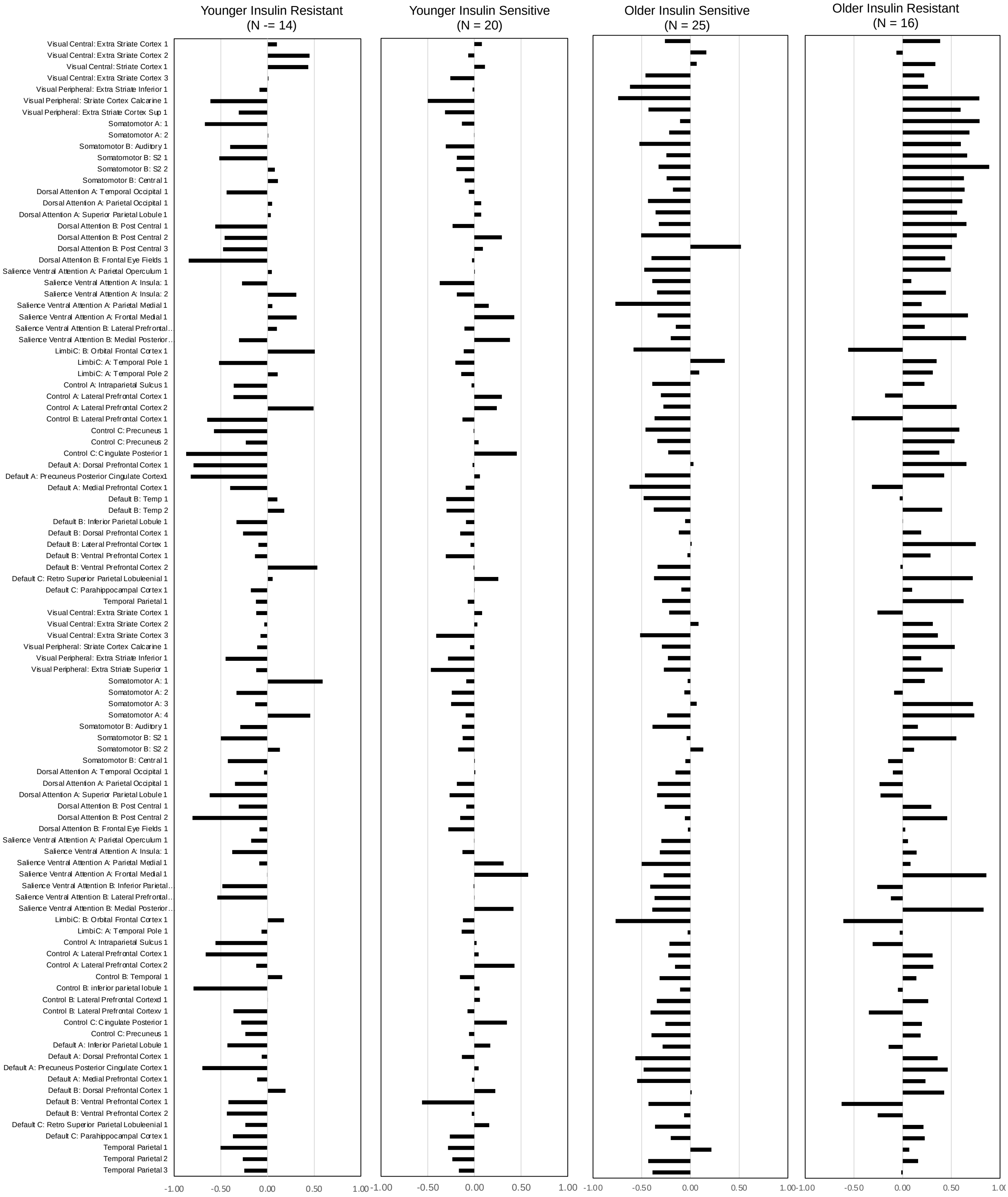

Figure S5. Partial correlation of regional CBF and CMR_{GLC} controlling for cortical thickness blood pressure, resting heart rate, BMI, sex and years of education. Four groups based on age category and HOMA-IR levels: younger insulin sensitive; younger insulin resistant; older insulin sensitive; and older insulin resistant

3. Supplementary References

1. Weschler, D., *Wechsler Abbreviated Scale of Intelligence--Second Edition.* APA PsycTests. , 2011.
2. Shapiro, A.M., et al., *Construct and concurrent validity of the Hopkins Verbal Learning Test-revised.* Clin Neuropsychol, 1999. **13**(3): p. 348-58.
3. Blackburn, H.L. and A.L. Benton, *Revised administration and scoring of the digit span test.* J Consult Psychol, 1957. **21**(2): p. 139-43.
4. Friedman, D., et al., *Age-related changes in executive function: an event-related potential (ERP) investigation of task-switching.* Neuropsychol Dev Cogn B Aging Neuropsychol Cogn, 2008. **15**(1): p. 95-128.
5. Verbruggen, F., G.D. Logan, and M.A. Stevens, *STOP-IT: Windows executable software for the stop-signal paradigm.* Behav Res Methods, 2008. **40**(2): p. 479-83.
6. Thorndike, E.L., *A Standardized group examination of intelligence independent of language.* Journal of Applied Psychology, 1919. **31**: p. 13-32.
